# The response of zircon to the extreme pressures and temperatures of a lightning strike

**DOI:** 10.1038/s41598-021-81043-8

**Published:** 2021-01-15

**Authors:** Gavin G. Kenny, Matthew A. Pasek

**Affiliations:** 1 Department of Geosciences, Swedish Museum of Natural History, 104 05 Stockholm, Sweden; 2 School of Geosciences, University of South Florida, Tampa, FL 33620 USA

**Keywords:** Mineralogy, Petrology, Mineralogy, Petrology

## Abstract

Hypervelocity impacts can produce features in zircon that are not normally produced by endogenic processes. However, lightning can also induce extreme pressure–temperature excursions, and its effect on zircon has not been studied. With the aim to recognise features that form in response to extreme pressure–temperature excursions but are not unique to hypervelocity impacts, we imaged and undertook microstructural characterization of zircon in a fulgurite (a tubular body of glass and fused clasts that formed in response to a lightning strike). We document zircon with granular ZrO_2_ and rims of vermicular ZrO_2_, features which vary in abundance with increasing distance from the fulgurite’s central void. This indicates that these features formed in response to the lightning strike. Zircon dissociation to ZrO_2_ and SiO_2_ is a high-temperature, relatively low-pressure phenomenon, consistent with previous suggestions that lightning strikes involve extreme temperatures as well as pressures greater than those usually generated in Earth’s crust but rarely > 10 GPa. The rims of monoclinic ZrO_2_ record crystallographic evidence for precursor cubic ZrO_2_, demonstrating that cubic ZrO_2_ is not unique to hypervelocity impacts. Given the likelihood that this fulgurite experienced pressures of, at most, a few GPa, evidence for cubic ZrO_2_ indicates peak temperatures > 2000 °C.

## Introduction

Hypervelocity impacts induce extreme pressures and temperatures that are not normally produced by endogenic geological processes. Features created in response to these conditions can thus be used to infer the occurrence of a bolide impact and confirm a suspected impact structure (planar deformation features, PDFs, in quartz and the high-pressure SiO_2_ polymorphs coesite and stishovite are perhaps the best known of these; e.g. ref.^1^). However, lightning strikes are also known to cause extreme pressure–temperature perturbations and are a common phenomenon on Earth and other planetary bodies. Given the increasing use of zircon (ZrSiO_4_) in studies of shock metamorphism and impact cratering (e.g., ref.^2^ and references therein), there is an imperative to understand which features in zircon are unique to hypervelocity impacts and which may form as a result of other extreme pressure–temperature excursions, such as lightning strikes. There has not yet been a systematic investigation of how zircon responds to lightning, but at least two studies of glass produced by a lightning strike have noted zircon grains with rims of ZrO_2_^3,4^ and one study documented a Zr-rich patch of glass that was interpreted to represent a zircon grain that had been entirely melted^3^.

Here we report imaging and microstructural analysis of zircon exposed to a lightning strike, as well as imaging of control samples, i.e. zircon from nearby soil and rock that was not affected by the lightning strike. We document different responses of zircon to different peak pressure–temperature conditions, and present the first crystallographic evidence for the former presence of cubic ZrO_2_ (which, at low pressures, only forms at > 2370 °C) in glass produced by a lightning strike, demonstrating that this phase is not unique to hypervelocity impacts.

### Zircon at extreme pressures and temperatures

Features in zircon that have been proposed as unique to the extreme pressures of a hypervelocity impact include: (a) deformation twinning along {112} crystallographic planes, which has been observed in diamond anvil experiments at 20 GPa^5^ (however, ~ 20 GPa is considered a tentative estimate for the minimum formation pressure under impact conditions and the effects of temperature on twin formation have yet to be evaluated^2^) (b) the presence of reidite (the high-pressure polymorph of zircon), which, experiments indicate, appears to form at pressures in excessive of ~ 30 GPa^5–7^; and (c) systematically misoriented neoblasts in granular zircon that have been interpreted to indicate reversion from reidite^2,8,9^. On the other hand, the dissociation of zircon to zirconia (ZrO_2_) and silica (SiO_2_) is primarily regulated by temperature^2,10,11^. Minor occurrences of ZrO_2_ in granular zircon (e.g., refs.^8,12,13^) is indicative of zircon dissociation, experimentally constrained to occur above 1673 °C at 1 atm^2,10,11^, whereas the formation of rims of vermicular zirconia around zircon grains can indicate temperatures in excess of 2370 °C^14,15^. Confirmation of the latter requires reconstruction of the polymorphic transformation history of this zirconia rim from microstructural data (so-called ‘phase heritage’^14^); a rim of monoclinic zirconia (baddeleyite) can preserve crystallographic relationships (namely, spatially-clustered baddeleyite grains with distinctive patterns of up to twelve orientations) that uniquely identify the former presence of a precursor cubic zirconia polymorph^14,15^.

Age resetting appears to be variably related to pressure and/or temperature. Impact ages are often recorded in partially to completely recrystallized (granular, neoblastic) zircon grains, with recrystallization being thermally driven in many cases (e.g., refs.^13,16^) and interpreted to be related to reversion from reidite in others (e.g., ref.^17^) (although even this reversion is restricted to temperatures above 1200 °C^6,8,9^). Porous textures in zircon from impact melt rocks and impact melt-bearing breccias (e.g., refs.^18,19^) have also been shown to accurately record impact ages. As such, it is important to understand if porosity in zircon is an inherited pre-impact feature of the grains or if it can form in impact environments (for example, in response to extreme pressures and temperatures or in response to post-impact hydrothermal alteration).

### Pressure and temperature conditions of a lightning strike

Lightning is a ubiquitous natural phenomenon whereby an electrostatic discharge dissipates up to 1 GJ (of which ~ 1 MJ may reach the ground)^20^, and instantaneously heats surrounding air to 10^4^–10^5^ K^21,22^. When lightning strikes and travels through sand or soil it vaporizes, melts, and fuses material, producing a tubular body of glass and fused clasts known as a fulgurite. Fulgurites can be divided into four to five main groups on the basis of morphology, which is usually a function of target material^3^. According to the scheme of ref.^3^, type I fulgurites consist of thin, glass walls and form in sand; type II fulgurites consist of thick, melt-rich walls and form in soil; type III fulgurites consist of thick, glass-poor walls and form in caliche; type IV fulgurites consist of glass and form on rock surfaces; additionally, fulgurite droplets, which form when material is ejected from the main fulgurite body and subsequently lands and cools on the ground surface, can be considered a fifth, minor group^3^. Fulgurites that form in soil or sand often have a central void where material was entirely vaporized. The central void is surrounded by glass that, thermal modelling predicts, reached peak temperatures in excess of 3000 K (~ 2730 °C) 0.5–3 s after the lightning strike before cooling to below 1000 K (~ 730 °C) in approximately 2 min^3^. In addition to extreme temperatures, lightning strikes may also result in extreme pressures. Modelling of fulgurites formed on rock surfaces suggested that lightning can induce pressures > 7 GPa^23^, and modelling of fulgurite formation in soil suggested that maximum lightning-induced pressures may occasionally exceed 10 GPa^24^. Indirect evidence provided by neutron diffraction data has been used to suggest that pressures may even reach 25 GPa^25^. Evidence for extreme pressures also comes from quartz in fulgurites that appears shocked^26,27^, including grains that display lamellae similar to those produced during hypervelocity impacts^27^. Together, these studies suggest that lightning strikes may induce pressures far in excess of that usually generated in the Earth’s crust (generally < 1 GPa) but rarely in excess of 10 GPa.

### The York County fulgurite

**Figure 1:**
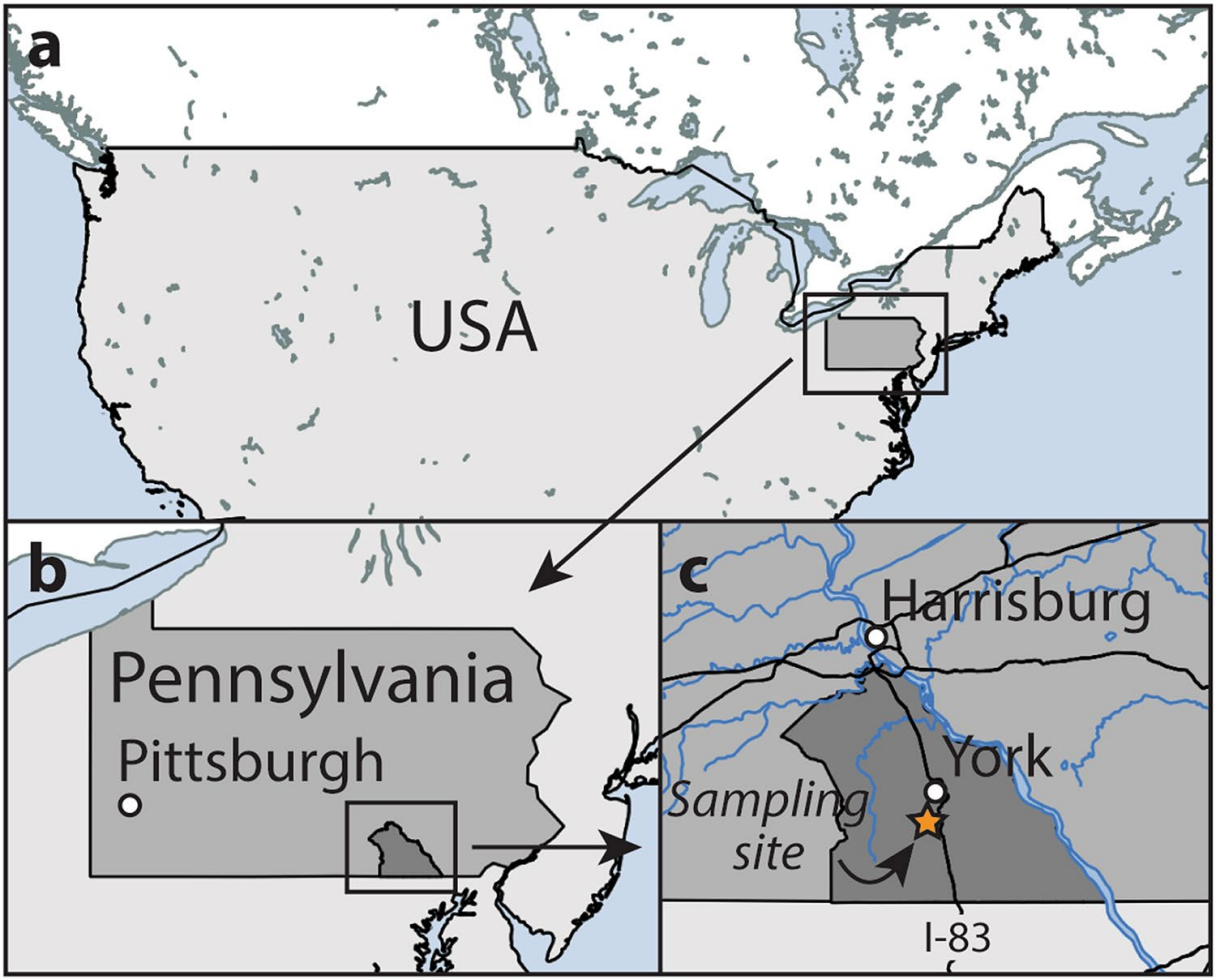
Map showing the location of sampling site for the York County fulgurite. In (**c**), black lines denote major roads (including Interstate-83) and blue lines denote major waterways. Maps created in QGIS version 2.0.1-Dufour (https://qgis.org), using copyright-free data, i.e. shapefiles, from the US Census Bureau (https://catalog.data.gov/dataset).

The York County fulgurite formed after an observed lightning strike in York County, Pennsylvania, USA (Fig. 1) in 2004 (coordinates of sampling site: 39.8904° N, 76.7371° W). The target material was vegetated soil containing small clasts from the underlying bedrock. The bedrock is mapped as the Harpers Formation^28^, a metasedimentary phyllite of late Proterozoic or early Cambrian age, and part of the Chilhowee Group^29^. Zircon is the most common heavy mineral in correlated formations^30^ and most detrital zircon grains of this group date to about 1 Ga^31^. In the field, the fulgurite was estimated to have a total mass of approximately 1 kg, distributed across a branched structure of ~ 1 m in total length. Approximately half of this was acquired by one of us (M. P.). For the purposes of this study, a small portion (~ 15 g) of the fulgurite was mounted in epoxy and cut perpendicular to its exterior wall. This exposed an approximately circular cross-section 20 mm in diameter and an approximately elliptical central void varying between 5 and 10 mm wide. The York County fulgurite has relatively thick, melt-rich walls and, as such, was classified as a type II fulgurite in ref.^3^. Previous work has indicated that approximately 10% of the York County fulgurite consists of lechatelierite (SiO_2_ glass) whereas the remaining glass has an average composition of approximately 50% SiO_2_, 29% Al_2_O_3_, 9% Fe_2_O_3_, 4% K_2_O, 3% TiO_2_, 2% MgO, and other components, such as CaO, Na_2_O, and P_2_O_5_, each comprising < 1%^3,32^.

Our study comprised two strands: (1) imaging and microstructural analysis of Zr-rich grains in a cross-section of the York County fulgurite, and (2) imaging of control zircon grains that were separated from nearby soil and bedrock that were not exposed to the lightning strike. An automated backscatter electron (BSE) map of the mount was acquired on an FEI Quanta FEG 650 scanning electron microscope (SEM) at the Swedish Museum of Natural History, Stockholm. Ninety-one Zr-rich grains (86 zircon grains and five grains composed entirely of vermicular ZrO_2_) were identified from this map and imaged at higher resolution in BSE and cathodoluminescence (CL) modes. Electron backscatter diffraction (EBSD) analysis was then performed on three grains of interest. Analysis of crystallographic orientation data for zirconia was undertaken in the ARPGE software^15,33^. The sample of soil collected from near the fulgurite (sample YPAcon1) was cleaned in water and dried before undergoing mineral separation with a Frantz magnetic separator and heavy liquids (methylene iodide with a density of ~ 3.3 g/cm^3^). Zircon grains were picked from the heavy liquid separates, mounted in epoxy, polished in order to expose their mid-sections, and imaged in BSE and CL modes. The rock sample collected from near the fulgurite (YPAcon3) was first disaggregated in a puck-and-ring-style mill before undergoing the same separation process as the soil sample. In order to ensure that we did not study only the most pristine zircon in the control samples, three fractions from the magnetic separation process underwent heavy liquid separation. These were (1) the fraction that was non-magnetic at 1.7 A, the highest current used (this fraction should contain the most pristine zircon grains), (2) the fraction that was non-magnetic at 1.5 A and magnetic at 1.7 A, and (3) the fraction that was non-magnetic at 1.0 A and magnetic at 1.5 A. The fractions that were magnetic at currents < 1.0 A contained many minerals and so zircon could not be identified in, or separated from, these fractions. It is important to note that some zircon may have been magnetic at < 1.0 A and, therefore, may have escaped our study. Consequently, our comparison of zircon in the two control samples with zircon in the fulgurite was purely qualitative, i.e. we noted what features occur in zircon in the control samples but did not place significance on their relative abundances.

## Results

### Imaging of zircon in soil and rock from near the fulgurite

**Figure 2:**
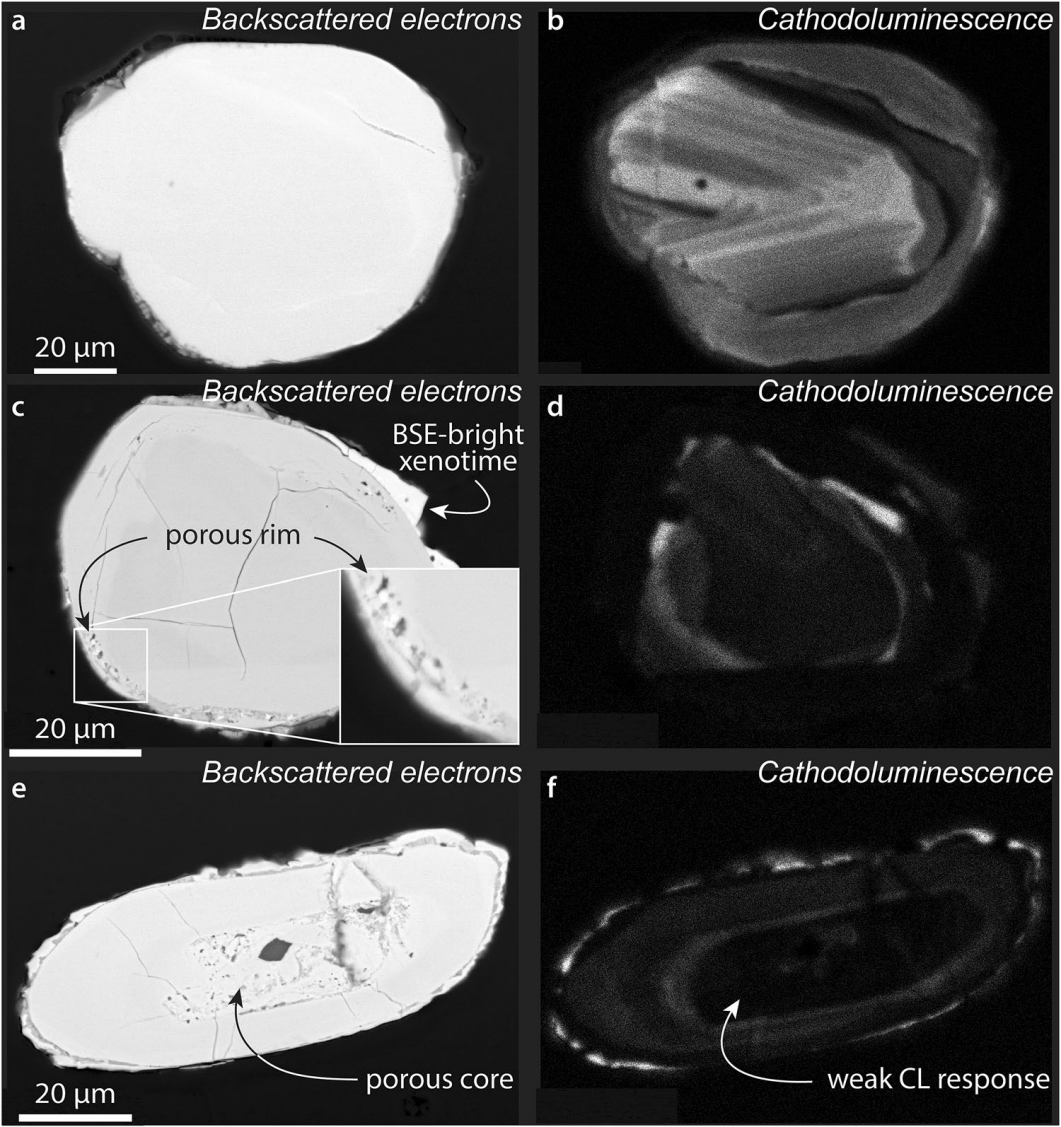
Imaging of zircon grains from soil and rock collected near the fulgurite but not affected by the lightning strike. These grains act as a control, showing how zircon in the fulgurite may have appeared if not subjected to the extreme conditions of the lightning strike. (**a**,**b**) and (**e**,**f**) from soil sample YPAcon3; (**c**,**d**) from rock sample YPAcon1. All images of zircon in samples YPAcon1 and YPAcon3 are available in Supplementary Figs. S1–S6.

We imaged over 200 zircon grains from each of rock sample YPAcon1 (Supplementary Figs. S1–S3) and soil sample YPAcon3 (Supplementary Figs. S4–S6) in BSE and CL modes. The vast majority of zircon in the control samples have homogeneous appearances in BSE images but the population displays a range of textures in CL (e.g., concentric growth zoning, and core–rim relationships; e.g., Fig. 2a,b) that are typical of terrestrial zircon (e.g., ref.^34^). Approximately 50% of zircon grains in rock sample YPAcon1, and a smaller proportion of grains in soil sample YPAcon3, have overgrowths that are up to ~ 10 µm thick and brighter than zircon in BSE images (e.g., Fig. 2c,d). Energy-dispersive X-ray (EDS) analysis indicated that these overgrowths are xenotime, a mineral known to commonly precipitate on zircon (e.g., ref.^35^ and references therein). A few grains in both control samples display porous textures in BSE that correspond to areas that are dark in CL. The porous areas are usually distinct growth zones, such as cores (e.g., Fig. 2e,f) or rims (e.g., Fig. 2c,d), and presumably resulted from damage to the crystal lattice, i.e., metamictization, from radioactive decay in relatively U- and Th-rich growth zones and/or hydrothermal or metamorphic alteration (e.g., ref.^36^).

### Imaging of the fulgurite

**Figure 3:**
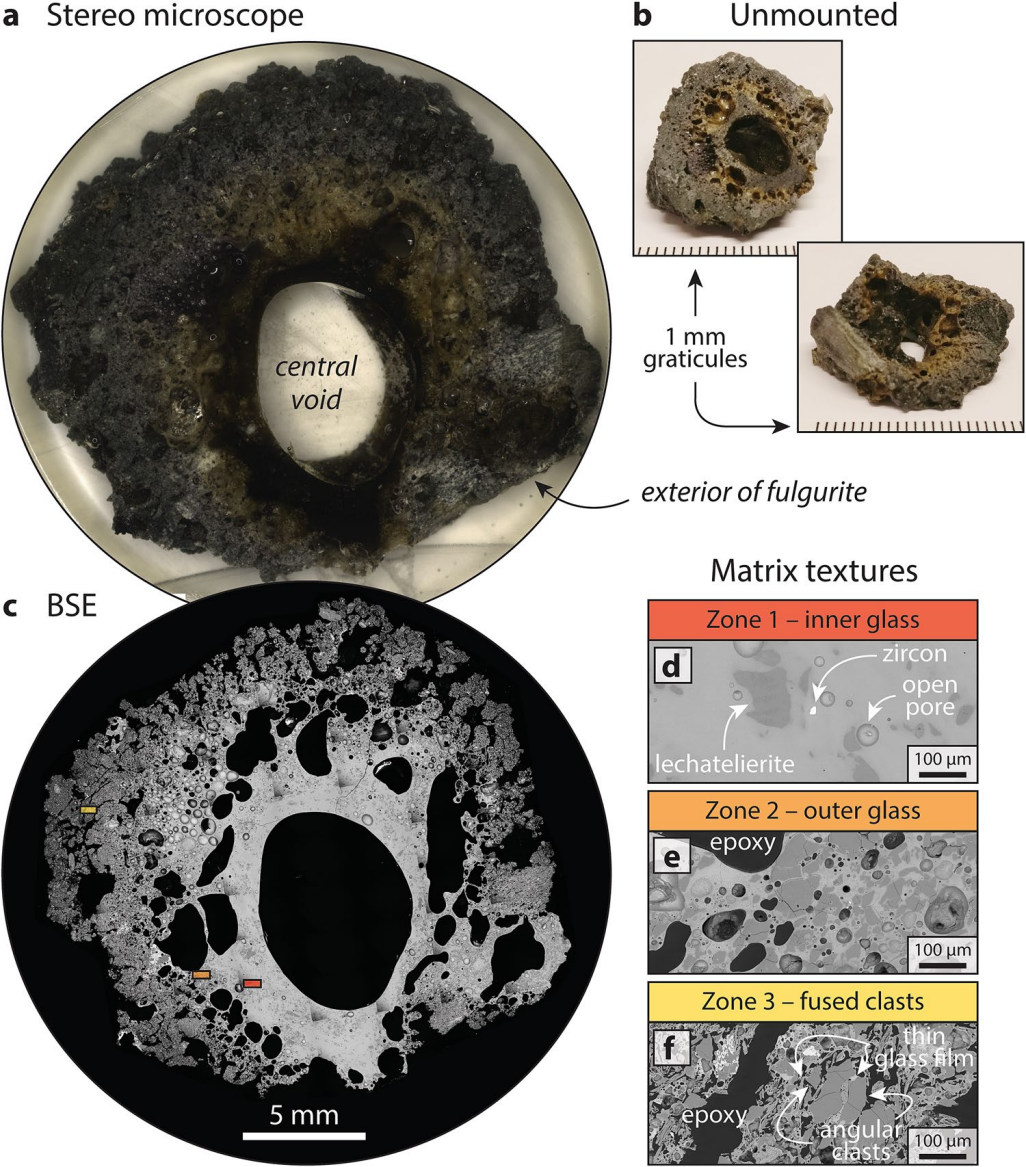
Imaging of York County fulgurite. (**a**) Image of the studied surface obtained on a stereo microscope. (**b**) Photographs of the specimen prior to mounting in epoxy. (**c**) Backscattered electron (BSE) mosaic of the studied surface. (**d**–**f**) High-resolution BSE images the three zones. Coloured boxes in (**c**) denote locations at which the images in (**d**–**f**) were acquired.

The studied cross-section of the York County fulgurite displays three distinct zones, two of which are predominantly composed of glass (Fig. 3). The central void is invariably surrounded by a 1–3 mm-thick region of black glass (Fig. 3a,b). This typically has a mafic to intermediate composition^18^ but also contains < 5% amorphous globules of lechatelierite and < 1% clasts (predominantly single crystals of zircon) (Fig. 3c,d). It is hereafter referred to as zone one. Zone one is surrounded by a 2–6 mm-thick mantle of green glass (Fig. 3a,b) that contains much more lechatelierite (~ 50%) and a similar amount of clasts (predominantly single crystals) (Fig. 3e). This is referred to as zone two. The outermost portion of the fulgurite is 3–4 mm thick and composed of > 90% clasts (both single crystals and polymineralic clasts) that are held together by a thin film of glass (Fig. 3c,f). This is referred to as zone three. Pores, ranging from the micrometre to millimetre scale, are present throughout the fulgurite, being most abundant in zone two (Fig. 3c).

### Imaging of zircon in the fulgurite

**Figure 4:**
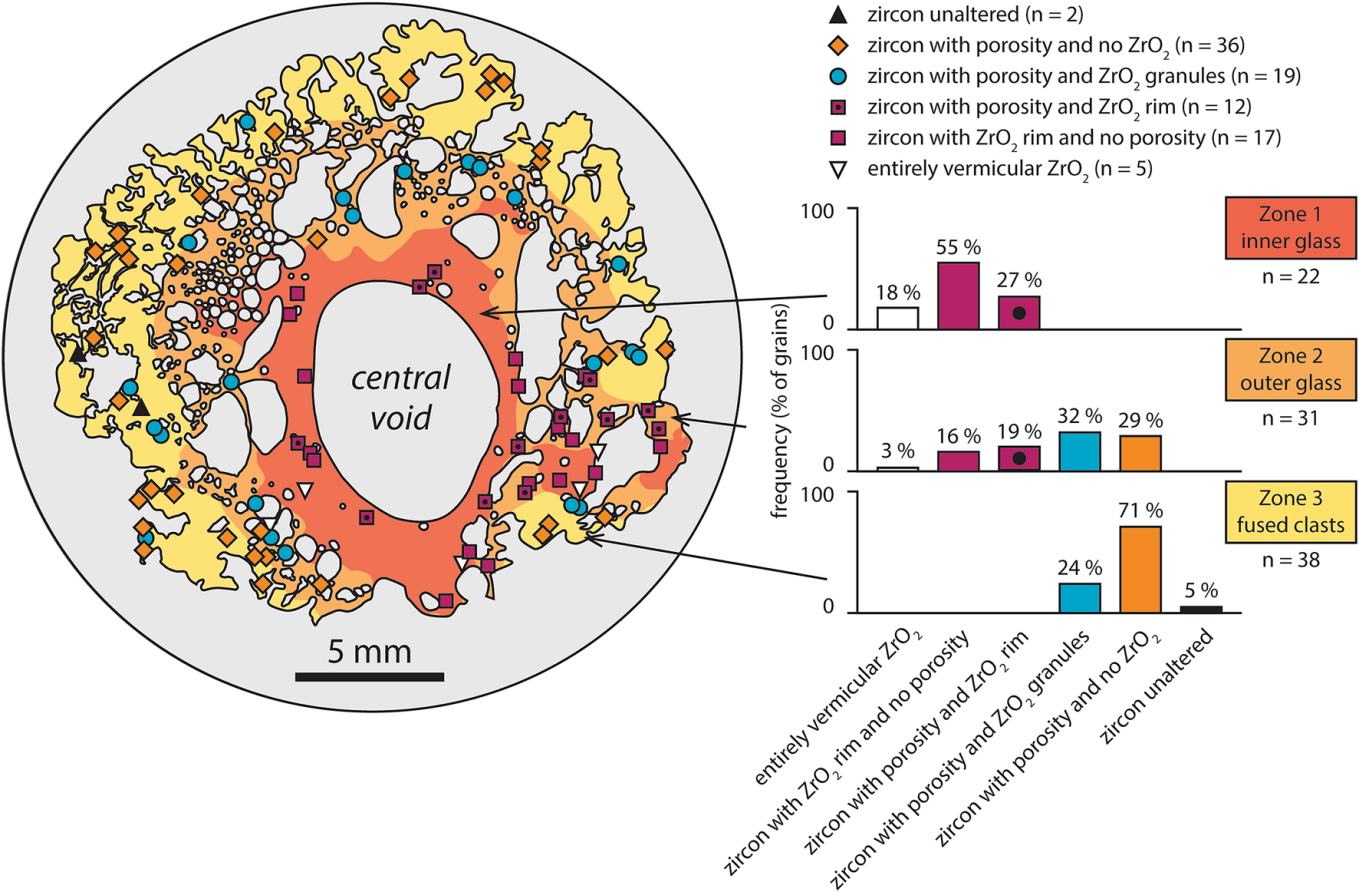
Schematic diagram of the York County fulgurite showing the location of Zr-rich grains and the distribution of zircon grains with porosity, ZrO_2_ granules, and ZrO_2_ rims. Percentages for zone two add up to 99% due to rounding.

**Figure 5:**
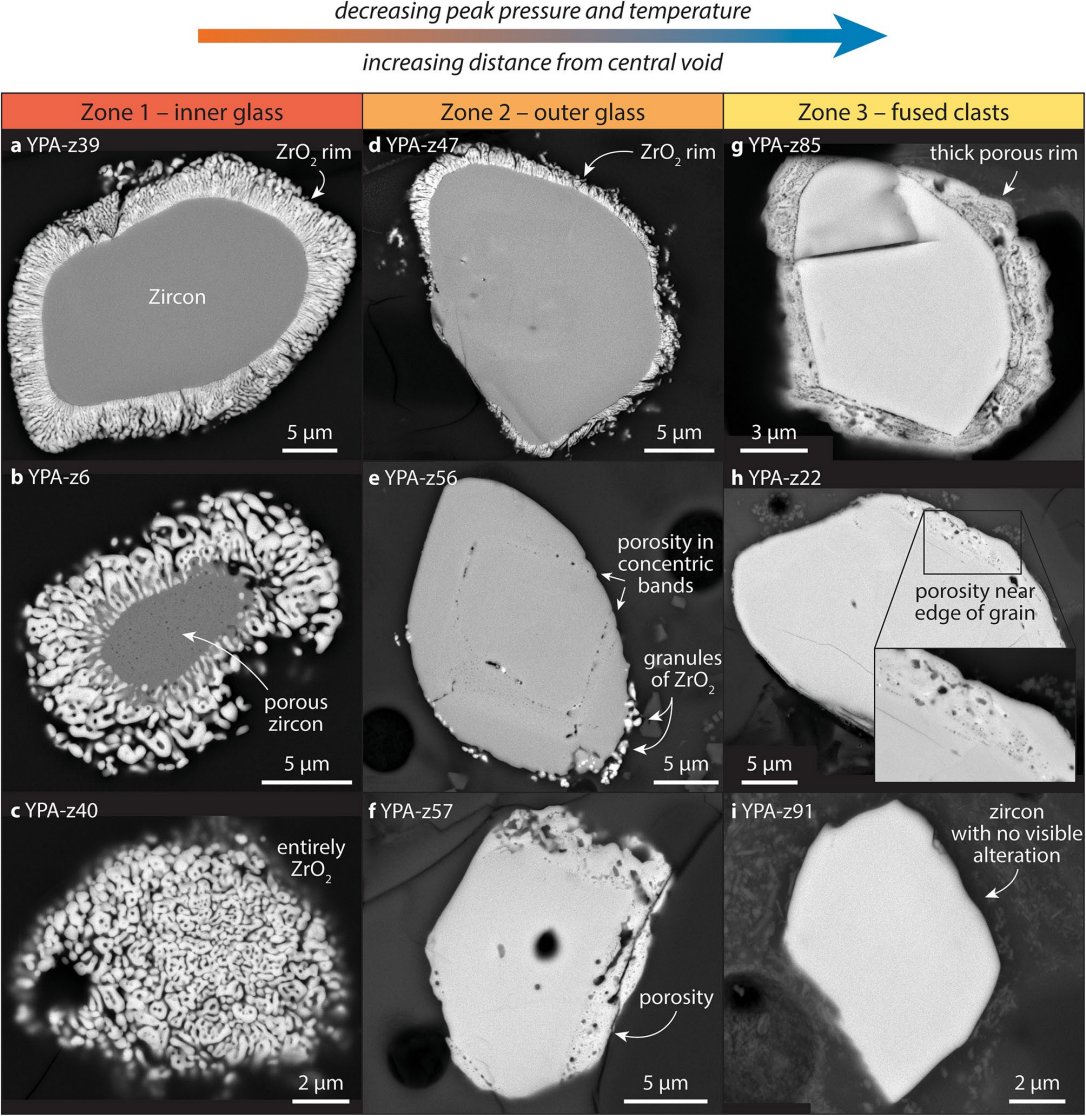
Backscattered electron images of Zr-rich grains in the York County fulgurite. Most grains are predominantly composed of zircon and one grain (YPA-z40 in **c**) is entirely composed of vermicular ZrO_2_ on the exposed surface. See Supplementary Figs. S7–S9 for backscattered electron and cathodoluminescence images of all 91 grains documented in the fulgurite.

**Figure 6:**
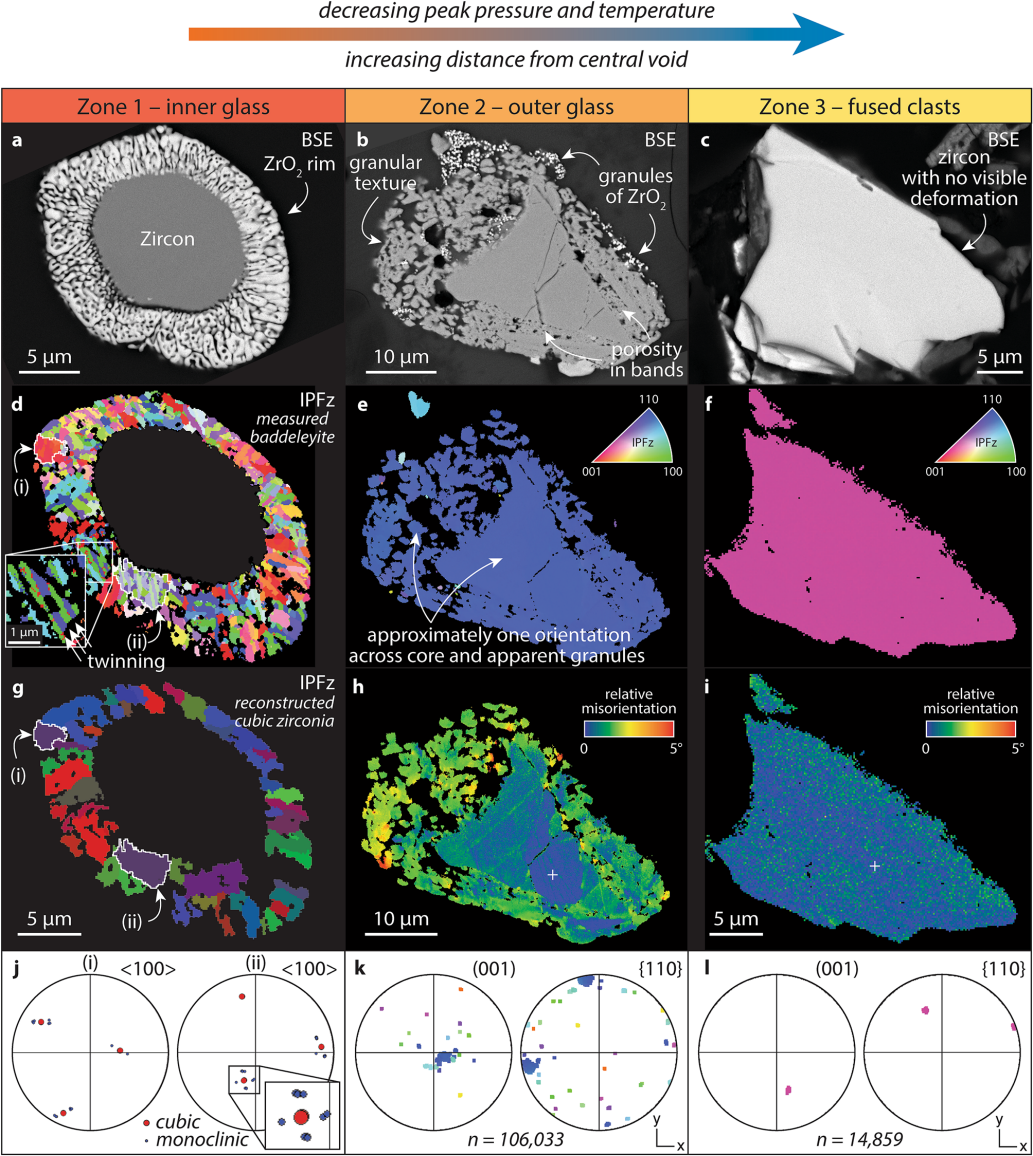
Microstructural characterization of zircon in the York County fulgurite. (**a**–**c**) Backscattered electron (BSE) imaging of grains YPA-z5, -z17, and -z87, respectively. (**d**) Electron backscatter diffraction map coloured for measured crystallographic orientation of baddeleyite. For clarity, zircon is not shown. Inverse pole figure (IPF) colour scheme. (**e**–**f**) Electron backscatter diffraction maps coloured according to IPF colour scheme. For clarity, baddeleyite in grain YPA-z17 is not shown. (**g**) Map showing crystallographic orientations of reconstructed ‘parent’ cubic zirconia grains. (**j**) Pole figures showing measured ‘daughter’ baddeleyite orientations (**d**) and reconstructed ‘parent’ cubic zirconia orientations (**g**) from two ‘parent’ grains, (i) and (ii). (**h**–**i**) Maps showing misorientation relative to a point (white cross). (**k**,**l**) Pole figures showing orientation data for grains in YPA-z17 and -z87, respectively; coloured according IPF colour scheme. Pole figures are lower hemisphere, equal area plots. Electron backscatter diffraction maps were acquired at step sizes of 75 nm (**d**), 50 nm (inset in **d**), 80 nm (**e**,**h**), and 150 nm (**f**,**i**).

Zircon grains in the York County fulgurite display a number of distinct textures, the occurrence and abundance of which vary with distance from the central void (Fig. 4; Supplementary Table S1). Twenty-two Zr-rich grains are exposed in zone one (Supplementary Fig. S7), with 18 of these being zircon grains with rims of vermicular ZrO_2_ (e.g., Figs. 5a,b, 6a). Four grains in zone one are entirely composed of vermicular ZrO_2_ (e.g., Fig. 5c). Six of the zircon grains in zone one also display sub-micrometre-scale pores (e.g., Fig. 5b). Of the 32 Zr-bearing grains in zone two (Supplementary Fig. S8), 11 display rims of vermicular ZrO_2_ (e.g., Fig. 5d) and just a single grain is composed entirely of vermicular ZrO_2_. In zircon grains in zone two that do not have rims of vermicular ZrO_2_, clusters or single occurrences of equant, sub-micrometre-sized granules of ZrO_2_ (e.g., Fig. 5e) are common (12 grains). Twenty-three zircon grains in zone two display micrometre-scale porosity, with porosity generally restricted to certain growth zones or close to grain edges (e.g., Fig. 5e,f). Some grains also display granular textures (e.g., Fig. 6b). Although porosity and granular textures are generally developed close to the edges of the grains, a few grains display an inverted relationship, whereby the centre of the grain has a granular texture and the surrounding area appears unaffected (Supplementary Fig. S8). Of the 38 Zr-rich grains observed in zone three (Supplementary Fig. S9), none is composed of vermicular ZrO_2_ or contains a rim of vermicular ZrO_2_. Zone three also contains fewer grains that display granules of ZrO_2_ (10) and the vast majority of grains display some form of porosity (35; e.g., Fig. 5g,h). Two grains in zone three appear unaltered (e.g., Figs. 5i, 6c). Approximately 10 zircon grains in zones two and three display xenotime overgrowths (Supplementary Figs. S8, S9) like those observed in the control samples (e.g., Fig. 2c,d).

### Electron backscatter diffraction analysis

Three zircon grains with textures of interest were selected for microstructural characterization by EBSD analysis. Electron backscatter diffraction analysis reveals that the ZrO_2_ rim on a zircon grain in zone one is composed of monoclinic zirconia, baddeleyite. The baddeleyite grains display a wide range of crystallographic orientations and are commonly twinned (e.g., Fig. 6d). Additionally, they form distinct clusters that are up to 5 μm in diameter (Fig. 6d,g), with each of these clusters preserving up to twelve (but usually less) unique but systematically related crystallographic orientation variants. Groups of grains are approximately orthogonal to each other, with systematic deviations from orthogonality resulting in distinct cross-shaped, or flower-petal, patterns on pole figures (Fig. 6j). This distinctive pattern of clustered baddeleyite grains with up to twelve orientations is indicative of the two-stage transformation from cubic to tetragonal to monoclinic zirconia^15^. Additionally, peaks in disorientation angles between ‘daughter’ grains of monoclinic zirconia occur at 90°, 115°, and 180° (Supplementary Fig. S10). This is consistent with cubic–monoclinic transformation twinning^15^ and provides further evidence for the former presence of cubic and tetragonal zirconia in this rim now composed solely of monoclinic zirconia.

Electron backscatter diffraction analysis of a zircon grain in zone two confirmed that sub-micrometre granules of ZrO_2_ observed to rim a minority of grains are also baddeleyite. However, the extremely fine nature of these granules precluded analysis in ARPGE and thus no crystallographic evidence for the former presence for cubic zirconia could be collected or inferred. Microstructural characterization of a zircon grain with sub-micrometre scale porosity in certain growth zones and a coarser, apparently granular texture visible in BSE imaging (Fig. 6b) revealed that the micrometre-scale ‘granules’ of zircon share the same approximate crystallographic orientation as the rest of the grain (Fig. 6e,k) and that there is a maximum relative misorientation across the grain of approximately 5° (Fig. 6h).

Zircon grains from the outermost zone of the fulgurite show little deformation in EBSD maps, with one example, which is free of ZrO_2_ and porosity, displaying relative misorientation across the grain of less than 3° (Fig. 6f,i,l).

## Discussion

**Figure 7:**
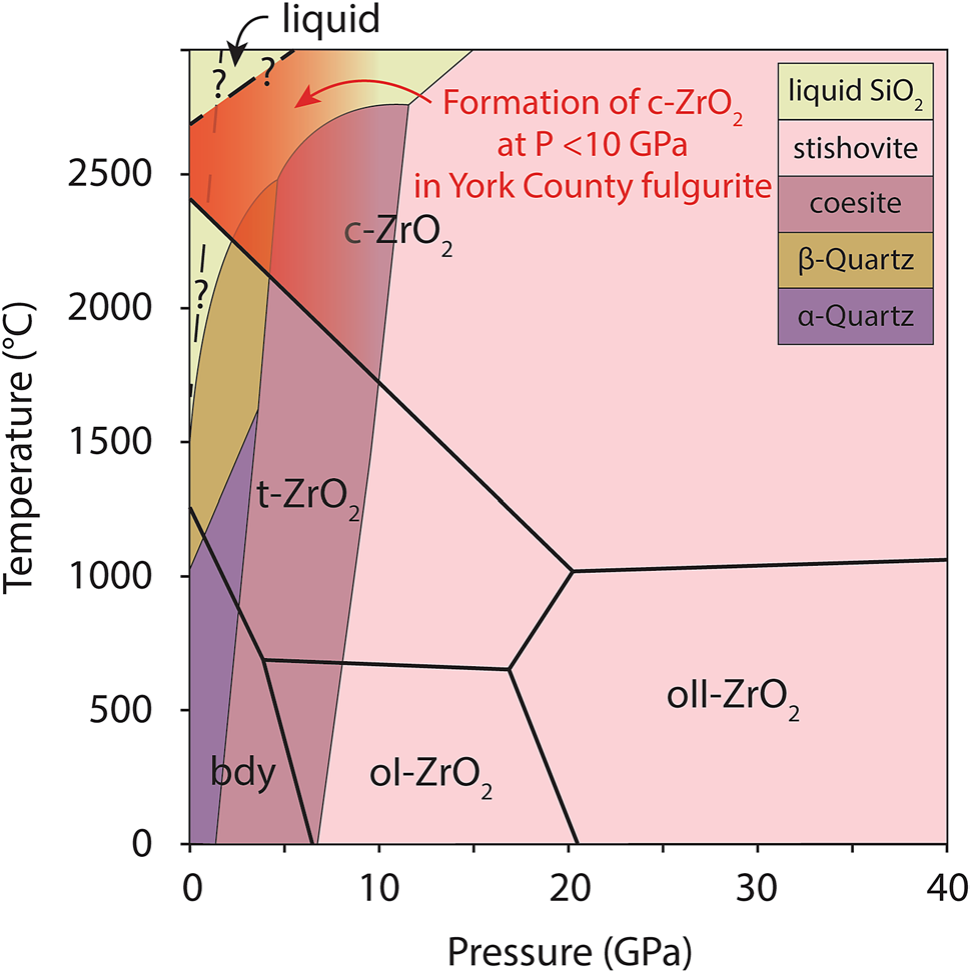
Stability fields for polymorphs of zircon dissociation products, SiO_2_ and ZrO_2_ (modified after ref.^2^, which built on diagrams of, most notably, refs.^10,11,38,43^). Polymorphs of SiO_2_ are shown with thin lines and coloured fields and polymorphs of ZrO_2_ are shown with thicker lines and annotated fields. *Bdy* baddeleyite, *c* cubic, *o* orthorhombic, *t* tetragonal. Dashed lines and question marks reflect (1) uncertainty in the up-temperature continuation of the zircon dissociation reaction line due to unknown effects of liquid silica on phase stability^2^, and (2) uncertainty in the temperatures at which cubic ZrO_2_ melts at high pressures^38^. Red shading indicates the likely formation conditions of cubic ZrO_2_ in the York County fulgurite given estimated pressures of up to a few GPa (see text).

Granular ZrO_2_ and rims of vermicular ZrO_2_ occur in zircon in the York County fulgurite but not the control sample. Additionally, the abundance of these features varies systemically with increasing distance from the fulgurite’s central void. We consider this unequivocal evidence that granular and vermicular ZrO_2_ in zircon formed in response to the lightning strike and we use these features to constrain pressure–temperature conditions reached in the fulgurite. Zircon dissociates to ZrO_2_ and SiO_2_ at ~ 1675 °C at zero pressure (note that SiO_2_ melts with just ~ 10 °C further heating and so it is expected to almost always move into the surrounding glass and not be preserved as a crystalline phase)^2,10,11^. Although the reaction line is not well constrained in P–T space (Fig. 7), zircon dissociation appears to be a high-T, low-P process^2,10,11^. Crystallographic evidence for the former presence of cubic ZrO_2_ in monoclinic ZrO_2_ rims can indicate even higher temperatures than simple zircon dissociation to ZrO_2_ and SiO_2_. Cubic ZrO_2_ forms at temperatures in excess of 2370 °C at low pressures, decreasing to ~ 1750 °C at 10 GPa^2,38^ (Fig. 7). Timms et al.^14^ documented evidence for naturally occurring cubic ZrO_2_ at a terrestrial impact structure and argued that evidence for low pressures at the time of formation (including the absence of high-pressure microstructures and polymorphs in zircon) means that the grain, and thus surrounding impact melt, reached temperatures > 2370 °C. Similarly, zircon in the York County fulgurite does not display any evidence for shock pressures, such as the high-pressure ZrSiO_4_ polymorph reidite or shock microtwins. However, it has been suggested that fulgurites can experience pressures of a few GPa, perhaps occasionally exceeding 10 GPa^23,24^, and consequently, we cannot assume that zircon grains in fulgurites only experienced atmospheric pressures. Given that much of the energy of a lightning strike goes into vaporizing material, the diameter of a fulgurite’s central void serves as a proxy for the energy per unit length deposited by the lightning strike, and thus likely pressures reached^37^. The York County fulgurite’s relatively small internal diameter of approximately 0.5 cm indicates that it was formed by a relatively low energy event (~ 0.5 MJ/m)^3,37^. Thus, using the relationship between lightning energy and peak shock pressures from ref.^24^, we suggest that it may have experienced, at most, a few GPa. The likelihood of such relatively low pressures means that cubic ZrO_2_ in the York County fulgurite likely formed at temperatures in excess of ~ 2000 °C. Cubic zirconia melts at ~ 2700 °C at low pressure, and at higher temperatures at higher pressures (ref.^38^ and references therein; Fig. 7). If cubic ZrO_2_ in the York County fulgurite had melted it would not have preserved crystallographic phase heritage^14^, and therefore, we can conclude that temperatures did not exceed ~ 2700 °C if pressures were close to atmospheric, or ~ 3000 °C if pressures reached ~ 5 GPa (Fig. 7). These pressure–temperature estimates are consistent with thermal modelling, which indicates that material surrounding the central void of a fulgurite such as this (i.e., a type II fulgurite) would have reached peak temperatures in excess of 3000 K (~ 2730 °C) 0.5–3 s after the lightning strike^3^. After reaching peak temperatures, fulgurites such as the York County specimen cooled below 1000 K (727 °C) within approximately 2 min^3^, indicating that vermicular monoclinic ZrO_2_ with crystallographic evidence for the former presence of cubic ZrO_2_ can develop extremely rapidly.

Evidence that cubic ZrO_2_ can occur in fulgurites indicates that this phase, despite only forming under extreme pressure–temperature conditions, is not unique to hypervelocity impacts. Context is, therefore, critical to interpreting whether this high-temperature phase formed in response to a lightning strike, impact event, or other extreme pressure–temperature excursion. For example, petrographic context enabled White et al.^39^ to interpret evidence for precursor cubic ZrO_2_ in a lunar sample as indicative of crystallization from a superheated impact melt^39^. Conversely, documenting precursor cubic ZrO_2_ in glasses of unknown origin would be insufficient to infer an impact origin for such samples.

Porous textures are common in zircon in the York County fulgurite but similar textures were also observed in zircon from the control samples. Although porous textures in zircon are much more common in the fulgurite (~ 70% of grains) than in the control grains imaged (< 5%), the process of magnetically separating zircon from other minerals in the control samples may have preferentially removed porous grains. Furthermore, in contrast to granular and vermicular ZrO_2_, it is not clear that porous textures in zircon vary systematically with increasing distance from the central void of the fulgurite. We documented significantly more zircon grains with porous textures in zones two and three than in the innermost zone, zone one (Fig. 4). However, the apparent rarity of porous textures in zircon in zone one may be due to the formation of rims of vermicular ZrO_2_, and even the transformation of entire grains (or, at least, entire exposed surfaces) to ZrO_2_, erasing evidence of porosity (which most commonly occurs close to zircon grain edges in the control samples and the outer zones of the fulgurite). As such, the degree to which porous textures in zircon in the York County fulgurite are related to the lightning strike is uncertain and further work is required to understand the formation of porous textures in zircon subjected to extreme pressures and temperatures.

Similar to porosity in zircon, xenotime overgrowths on zircon were observed in the control samples and in the fulgurite. Consequently, the overgrowths are considered original features of the grains and unrelated to the lightning strike.

Some grains in the York County fulgurite display granular textures (e.g., Fig. 6b). This texture may have resulted from extensive development of porosity around these relict ‘islands’ of zircon or it may indicate in situ recrystallization of the grain. Microstructural analysis of a zircon grain that displays granular textures on approximately half of its exposed surface (Fig. 6b) reveals that the granules share the same crystallographic orientation as the undeformed core of the grain, with less than 5° relative misorientation across the grain surface (Fig. 6e,h). Zircon exposed to extreme pressure–temperature conditions in the course of the impact cratering process can recrystallize in a number of ways, including recrystallizing with the same orientation as the host grain^2^. Another style of recrystallization in impacted zircon results in systematically misoriented granules that do not share the orientation of the host grain. This has been interpreted to indicate reversion of reidite to zircon at temperatures > 1200 °C and thus as indirect evidence for the shock pressures required to form reidite^2,8,9^. The lack of such systemically misoriented granules in zircon in the York County fulgurite is consistent with the absence of other indicators of shock pressures (reidite itself, and shock microtwins) in zircon in the sample. This, in turn, is consistent with lightning and fulgurite formation being predominantly high-temperature phenomena that may induce pressures in excess of those normally experienced in the Earth’s crust but rarely exceeding 10 GPa (e.g., refs.^23,24^).

## Conclusions

- An observed lightning strike in York County, Pennsylvania, USA, in 2004 resulted in high-temperature, relatively low-pressure dissociation of zircon to ZrO_2_ and SiO_2_.
- Rims of vermicular ZrO_2_ on zircon record crystallographic evidence for the former presence of high-temperature cubic ZrO_2_.
- The presence and distribution of granular and vermicular ZrO_2_ supports modelling that suggested the fulgurite reached peak temperatures in excess of 3000 K (~ 2730 °C) 0.5–3 s after the lightning strike and cooled below 1000 K (727 °C) within approximately 2 min^3^.
- Evidence for cubic ZrO_2_ in a fulgurite, a common product of lightning strikes worldwide, indicates that this phase, despite only forming under extreme pressure–temperature conditions, is not unique to hypervelocity impacts.
- Lightning strikes and fulgurites provide a unique opportunity to study the response of minerals such as zircon to extreme pressure–temperature excursions.

## Methods

Electron backscatter diffraction analysis was performed with an Oxford Instruments Nordlys detector attached to the FEI Quanta FEG 650 SEM. Grains were indexed for zircon, reidite, and monoclinic ZrO_2_ using match units based on crystallographic data from ref.^40^ (1 atm), ref.^41^ (0.69 GPa), and ref.^42^, respectively. Analytical conditions and parameters include: accelerating voltage of 20 kV, working distance ~ 18 mm, stage tilt of 70°, electron backscatter pattern (EBSP) acquisition speed: 40 Hz; EBSP binning of 4 × 4, EBSP gain set to ‘High’, and background defined with collection of 128 frames, Hough resolution set to 60, band detection min/max of 6/8. Maps were collected with step sizes between 50 and 150 nm. Data collection was performed in Oxford Instruments AZtec software and post-acquisition processing in Oxford Instruments Channel 5 software v. 5.12. Data cleaning in Channel 5 comprised a wildspike correction and a level six, iterative, nearest neighbour zero solution extrapolation for all grains. This extrapolation was required so that grains would impinge on one another, enabling analysis of neighbour-pair disorientation relationships. These analyses, as well as reconstructions of ‘parent’ cubic zirconia grains from ‘daughter’ baddeleyite grains, were undertaken in the ARPGE software^15,33^.

## Supplementary Information

Supplementary Information.

## References

[1] French BM, Koeberl C (2010). The convincing identification of terrestrial meteorite impact structures: What works, what doesn’t, and why. Earth-Sci. Rev..

[2] Timms NE (2017). A pressure-temperature phase diagram for zircon at extreme conditions. Earth-Sci. Rev..

[3] Pasek MA, Block K, Pasek V (2012). Fulgurite morphology: A classification scheme and clues to formation. Contrib. Mineral. Petrol..

[4] Essene EJ, Fisher DC (1986). Lightning strike fusion: Extreme reduction and metal-silicate liquid immiscibility. Science.

[5] Morozova I, Shieh SR, Moser D, Barker IR, Hanchar JM, Moser DE, Corfu F, Darling JR, Reddy SM, Tait K (2018). Microstructural Geochronology: Planetary Records Down to Atom Scale.

[6] Kusaba K, Yasuhiko Syono MK, Fukuoka K (1985). Shock behavior of zircon: Phase transition to scheelite structure and decomposition. Earth Planet. Sci. Lett..

[7] Leroux H, Reimold WU, Koeberl C, Hornemann U, Doukhanc J-C (1999). Experimental shock deformation in zircon: A transmission electron microscopic study. Earth Planet. Sci. Lett..

[8] Cavosie AJ, Timms NE, Erickson TM, Hagerty JJ, Hörz F (2016). Transformations to granular zircon revealed: Twinning, reidite, and ZrO_2_ in shocked zircon from Meteor Crater (Arizona, USA). Geology.

[9] Cavosie AJ, Timms NE, Ferrière L, Rochette P (2018). FRIGN zircon—the only terrestrial mineral diagnostic of high-pressure and high-temperature shock deformation. Geology.

[10] Butterman WC, Foster WR (1967). Zircon stability and the ZrO_2_–SiO_2_ phase diagram. Am. Mineral..

[11] Kaiser A, Lobert M, Telle R (2008). Thermal stability of zircon (ZrSiO_4_). J. Eur. Ceram. Soc..

[12] Wittmann A, Kenkmann T, Schmitt RT, Stöffler D (2006). Shock-metamorphosed zircon in terrestrial impact craters. Meteorit. Planet. Sci..

[13] McGregor M, McFarlane CRM, Spray JG (2018). In situ LA-ICP-MS apatite and zircon U-Pb geochronology of the Nicholson Lake impact structure, Canada: Shock and related thermal effects. Earth Planet. Sci. Lett..

[14] Timms NE (2017). Cubic zirconia in>2370 °C impact melt records Earth's hottest crust. Earth Planet. Sci. Lett..

[15] Cayron C, Douillard T, Sibil A, Fantozzi G, Sao-Jao S (2010). Reconstruction of the cubic and tetragonal parent grains from electron backscatter diffraction maps of monoclinic zirconia. J. Am. Ceram. Soc..

[16] Moser DE (2011). New zircon shock phenomena and their use for dating and reconstruction of large impact structures revealed by electron nanobeam (EBSD, CL, EDS) and isotopic U-Pb and (U-Th)/He analysis of the Vredefort Dome. Can. J. Earth Sci..

[17] Kenny GG (2019). A new U-Pb age for shock-recrystallised zircon from the Lappajärvi impact crater, Finland, and implications for the accurate dating of impact events. Geochim. Cosmochim. Acta.

[18] Kenny GG (2020). The age of the Sääksjärvi impact structure (Finland): Reconciling the timing of small impacts in crystalline basement with that of regional basin development. J. Geol. Soc. Lond..

[19] McGregor M, Dence MR, McFarlane CRM, Spray JG (2020). U-Pb geochronology of apatite and zircon from the Brent impact structure, Canada: A Late Ordovician Sandbian-Katian boundary event associated with L-Chondrite parent body disruption. Contrib. Mineral. Petrol..

[20] Borucki WJ, Chameides WL (1984). Lightning: Estimates of the rates of energy dissipation and nitrogen fixation. Rev. Geophys..

[21] Uman MA (1978). An unusual lightning flash at Kennedy Space Center. Science.

[22] Rakov VA, Uman MA (2003). Lightning: Physics and Effects.

[23] Chen J, Elmi C, Goldsby D, Gieré R (2017). Generation of shock lamellae and melting in rocks by lightning-induced shock waves and electrical heating. Geophys. Res. Lett..

[24] Collins, G. S., Melosh, H. J., & Pasek, M. A. Can lightning strikes produce shocked quartz? *43rd Lunar and Planetary Science Conference*, abstract no. 1160 (2012).

[25] Ende M, Schorr S, Kloess G, Franz A, Tovar M (2012). Shocked quartz in Sahara fulgurite. Eur. J. Mineral..

[26] Carter EA (2010). Rapid Raman mapping of a fulgurite. Anal. Bioanal. Chem..

[27] Gieré R (2015). Lightning-induced shock lamellae in quartz. Am. Mineral..

[28] Lloyd, O. B. & Growitz, D. J. Geologic and hydrologic map of central and southern York County and southeastern Adams County, Pennsylvania. *Pennsylvania Bureau of Topographic and Geologic Survey Atlas,* W 42, plate 1 (1977).

[29] Schwab FL (1971). Harpers formation, central Virginia; a sedimentary model. J. Sediment. Res..

[30] Swingle, G. D. Petrography of the Chilhowee Group, Near Walland, Tennessee. Master’s Thesis, University of Tennessee, p 35 (1949).

[31] Eriksson KA, Campbell IH, Palin JM, Allen CM, Bock B (2004). Evidence for multiple recycling in Neoproterozoic through Pennsylvanian sedimentary rocks of the central Appalachian basin. J. Geol..

[32] Pasek M, Block K (2009). Lightning-induced reduction of phosphorus oxidation state. Nat. Geosci..

[33] Cayron C (2007). ARPGE: A computer program to automatically reconstruct the parent grains from electron backscatter diffraction data. J. Appl. Crystallogr..

[34] Corfu F, Hanchar JM, Hoskin PWO, Kinny P (2003). Atlas of zircon textures. Rev. Mineral. Geochem..

[35] Drost K, Wirth R, Košler J, Jørgensen HF, Ntaflos T (2013). Chemical and structural relations of epitaxial xenotime and zircon substratum in sedimentary and hydrothermal environments: A TEM study. Chem. Geol..

[36] Hay DC, Dempster TJ (2009). Zircon behaviour during low-temperature metamorphism. J. Petrol..

[37] Pasek MA, Hurst M (2016). A fossilized energy distribution of lightning. Sci. Rep..

[38] Bouvier P, Djurado E, Lucazeau G, Le Bihan T (2000). High-pressure structural evolution of undoped tetragonal nanocrystalline zirconia. Phys. Rev. B.

[39] White LF (2020). Evidence of extensive lunar crust formation in impact melt sheets 4,330 Myr ago. Nat. Astron..

[40] Hazen RM, Finger LW (1979). Crystal structure and compressibility of zircon at high pressure. Am. Mineral..

[41] Farnan I, Balan E, Pickard CJ, Mauri F (2003). The effect of radiation damage on local structure in the crystalline fraction of ZrSiO_4_: Investigating the ^29^Si NMR response to pressure in zircon and reidite. Am. Mineral..

[42] Howard CJ, Hill RJ, Reichert BE (1988). Structures of ZrO_2_ polymorphs at room temperature by high-resolution neutron powder diffraction. Acta Crystallogr. B.

[43] Swamy V, Saxena SK, Sundman B, Zhang J (1994). A thermodynamic assessment of silica phase diagram. J. Geophys. Res. Solid Earth.

