# Supplementary material for: The response of zircon to the extreme pressures and temperatures of a lightning strike
Source: Sci Rep. 2021 Jan 15;11:1560. doi: 10.1038/s41598-021-81043-8 (PMC7810979; doi:10.1038/s41598-021-81043-8)
Supplement: Supplementary file 1 — Supplementary Information. [file 41598_2021_81043_MOESM1_ESM.pdf]

The response of zircon to the extreme pressures and temperatures of a lightning strike

Gavin G. Kenny[1]* and Matthew A. Pasek[2]

[1]*Department of Geosciences, Swedish Museum of Natural History, SE-104 05 Stockholm, Sweden*

[2]*School of Geosciences, University of South Florida, Tampa, FL 33620, USA*

*gkennyeire@gmail.com

Supplementary materials

- **Supplementary Table 1.** Distribution of textures in zircon in a cross-section of the York County fulgurite.
- **Supplementary Fig. 1.** Backscattered electron (BSE) and cathodoluminescence (CL) imaging of zircon in non-magnetic-at-1.7 A fraction of control sample YPAcon1 (rock).
- **Supplementary Fig. 2.** Backscattered electron (BSE) and cathodoluminescence (CL) imaging of zircon in magnetic-at-1.7 A fraction of control sample YPAcon1 (rock).
- **Supplementary Fig. 3.** Backscattered electron (BSE) and cathodoluminescence (CL) imaging of zircon in magnetic-at-1.5 A fraction of control sample YPAcon1 (rock).
- **Supplementary Fig. 4.** Backscattered electron (BSE) and cathodoluminescence (CL) imaging of zircon in non-magnetic-at-1.7 A fraction of control sample YPAcon3 (soil).
- **Supplementary Fig. 5.** Backscattered electron (BSE) and cathodoluminescence (CL) imaging of zircon in magnetic-at-1.7 A fraction of control sample YPAcon3 (soil).

- **Supplementary Fig. 6.** Backscattered electron (BSE) and cathodoluminescence (CL) imaging of zircon in magnetic-at-1.5 A fraction of control sample YPAcon3 (soil).
- **Supplementary Fig. 7.** Backscattered electron (BSE) and cathodoluminescence (CL) imaging of all zircon grains from zone 1 (inner glass) of the York County fulgurite.
- **Supplementary Fig. 8.** Backscattered electron (BSE) and cathodoluminescence (CL) imaging of all zircon grains from zone 2 (outer glass) of the York County fulgurite.
- **Supplementary Fig. 9.** Backscattered electron (BSE) and cathodoluminescence (CL) imaging of all zircon grains from zone 3 (fused clasts) of the York County fulgurite.
- **Supplementary Fig. 10.** Results from orientation analysis of monoclinic zirconia, baddeleyite, in YPA-z5 (Fig. 3A-D) using ARPGE.

	Zone of fulgurite		
	Zone 1<br>inner glass	Zone 2<br>outer glass	Zone 3<br>fused clasts
Total number of Zr-rich grains	22	31	38
Entirely vermicular ZrO_2	4<br>(18 %)	1<br>(3 %)	0
Zircon with ZrO_2 rim and no porosity	12<br>(55 %)	5<br>(16 %)	0
Zircon with porosity and ZrO_2 rim	6<br>(27 %)	6<br>(19 %)	0
Zircon with porosity and ZrO_2 granules	0	10<br>(32 %)	9<br>(24 %)
Zircon with porosity and no ZrO_2 granules	0	9<br>(29 %)	27<br>(71 %)
Zircon unaltered	0	0	2<br>(5 %)

Supplementary Table 1. Distribution of textures in Zr-rich grains in a cross-section of the York County fulgurite. Percentages for zone 2 add up to 99 % due to rounding.

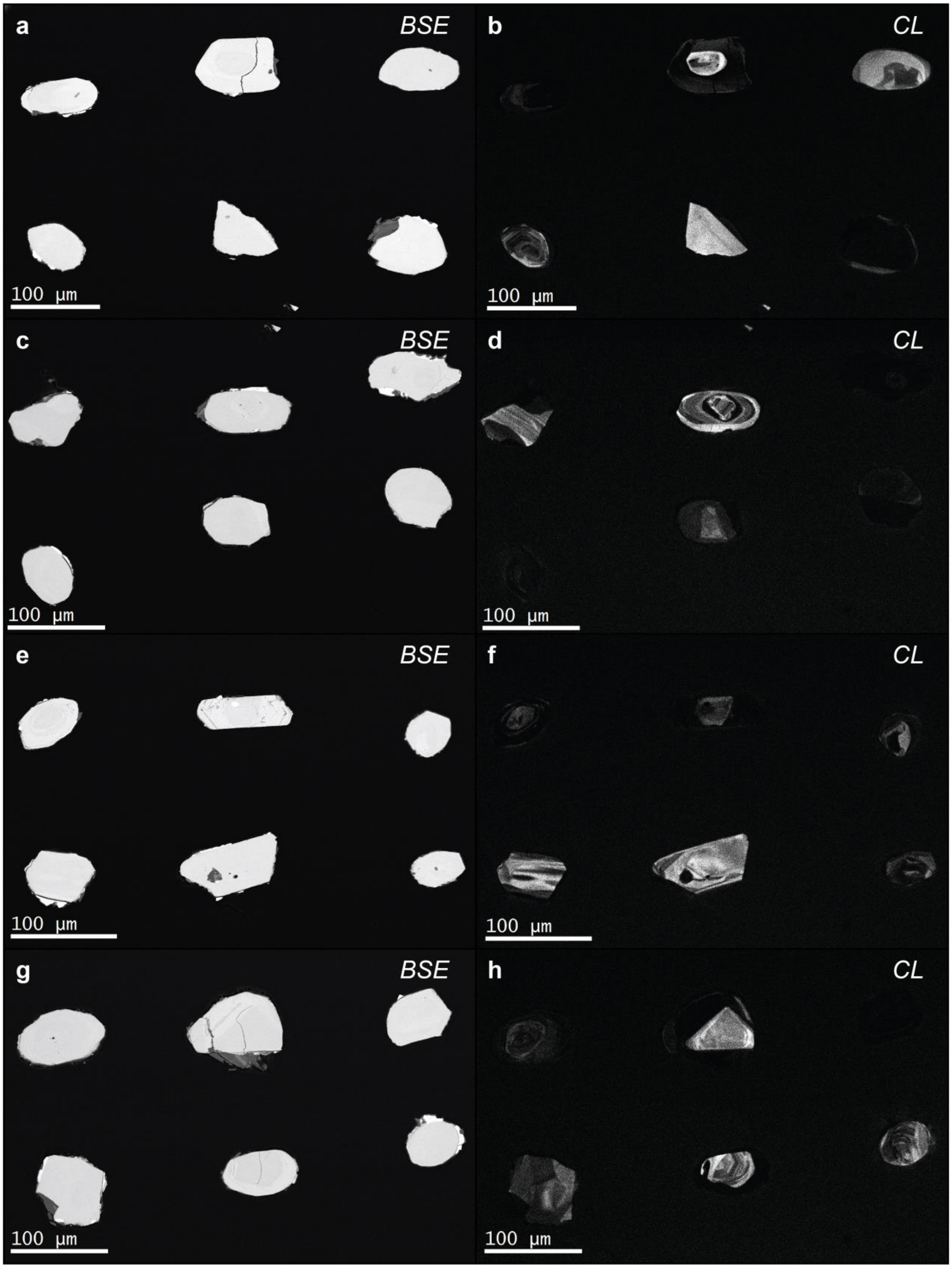

Supplementary Fig. 1. Backscattered electron (BSE), left panel, and cathodoluminescence (CL), right panel, imaging of zircon in non-magnetic-at-1.7 A fraction of control sample YPAcon1 (rock).

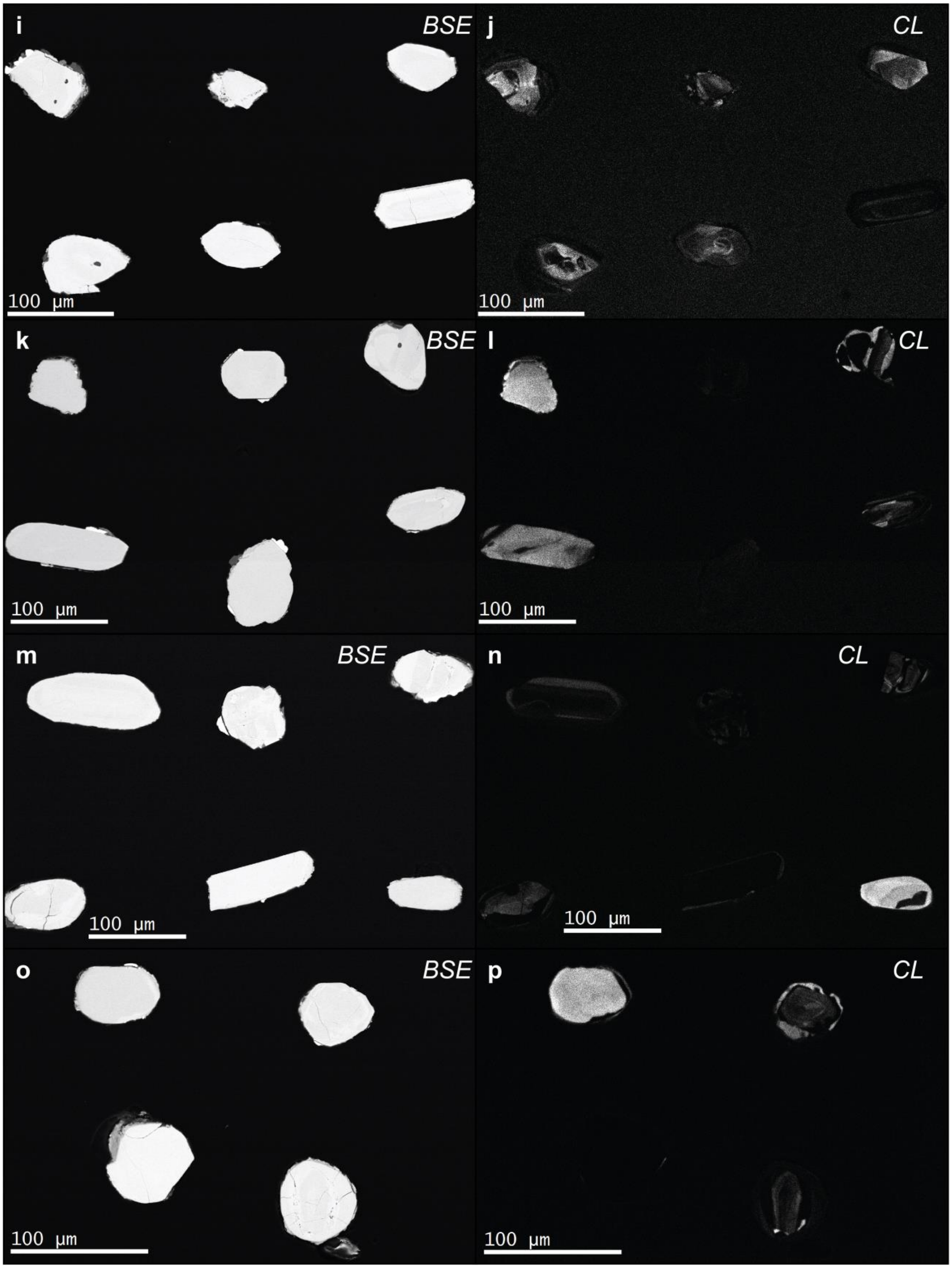

Supplementary Fig. 1 continued.

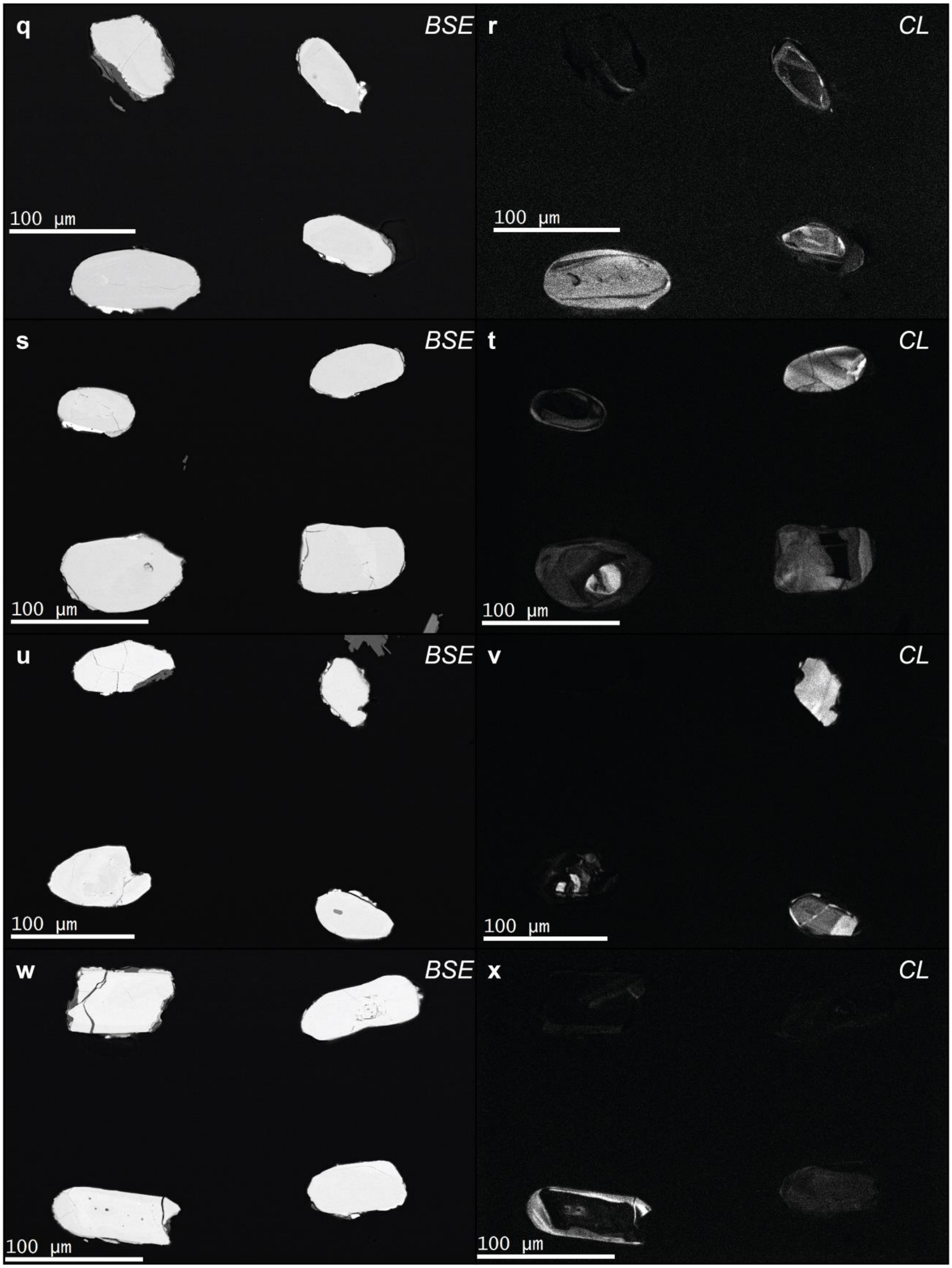

Supplementary Fig. 1 continued.

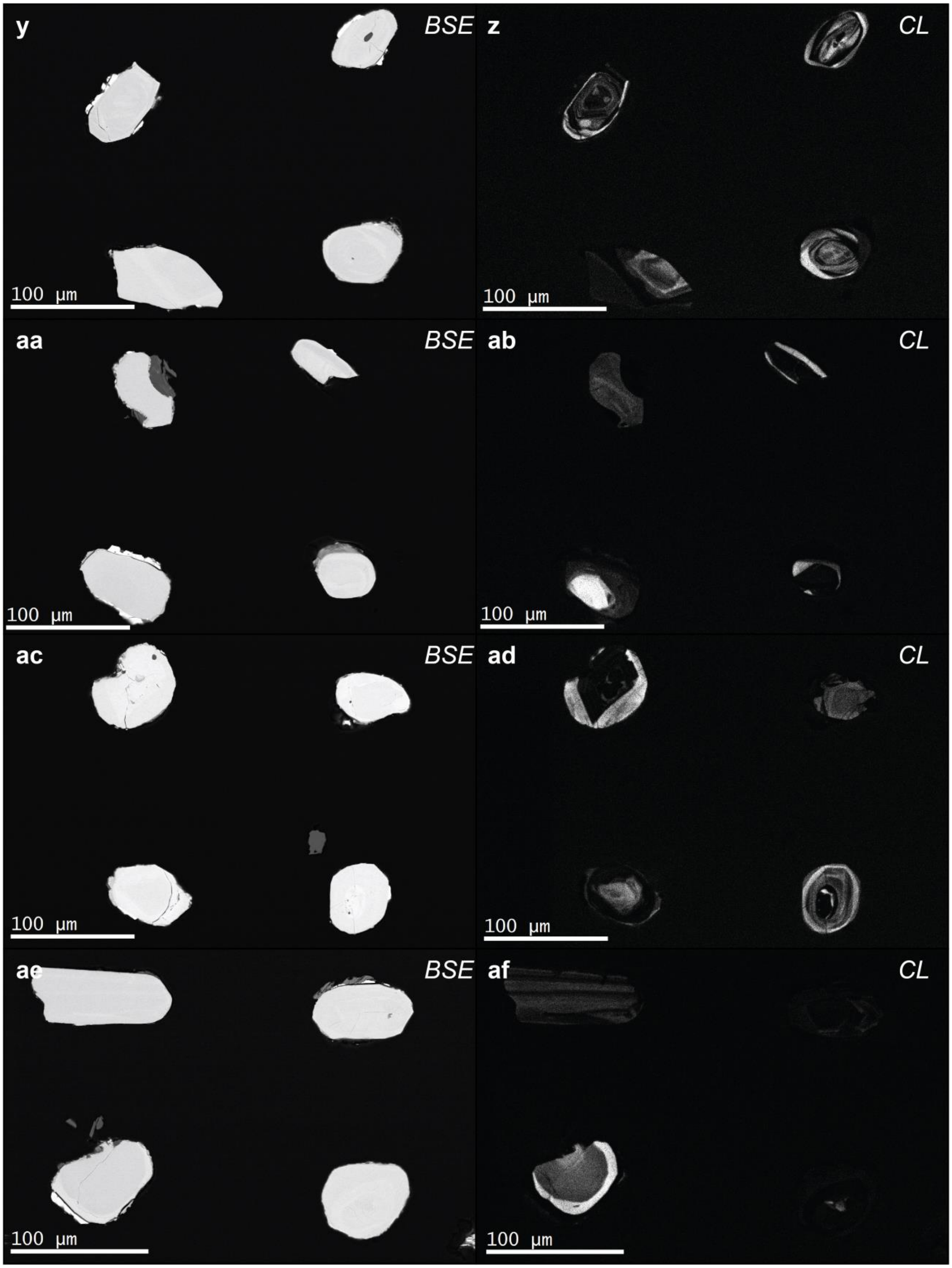

Supplementary Fig. 1 continued.

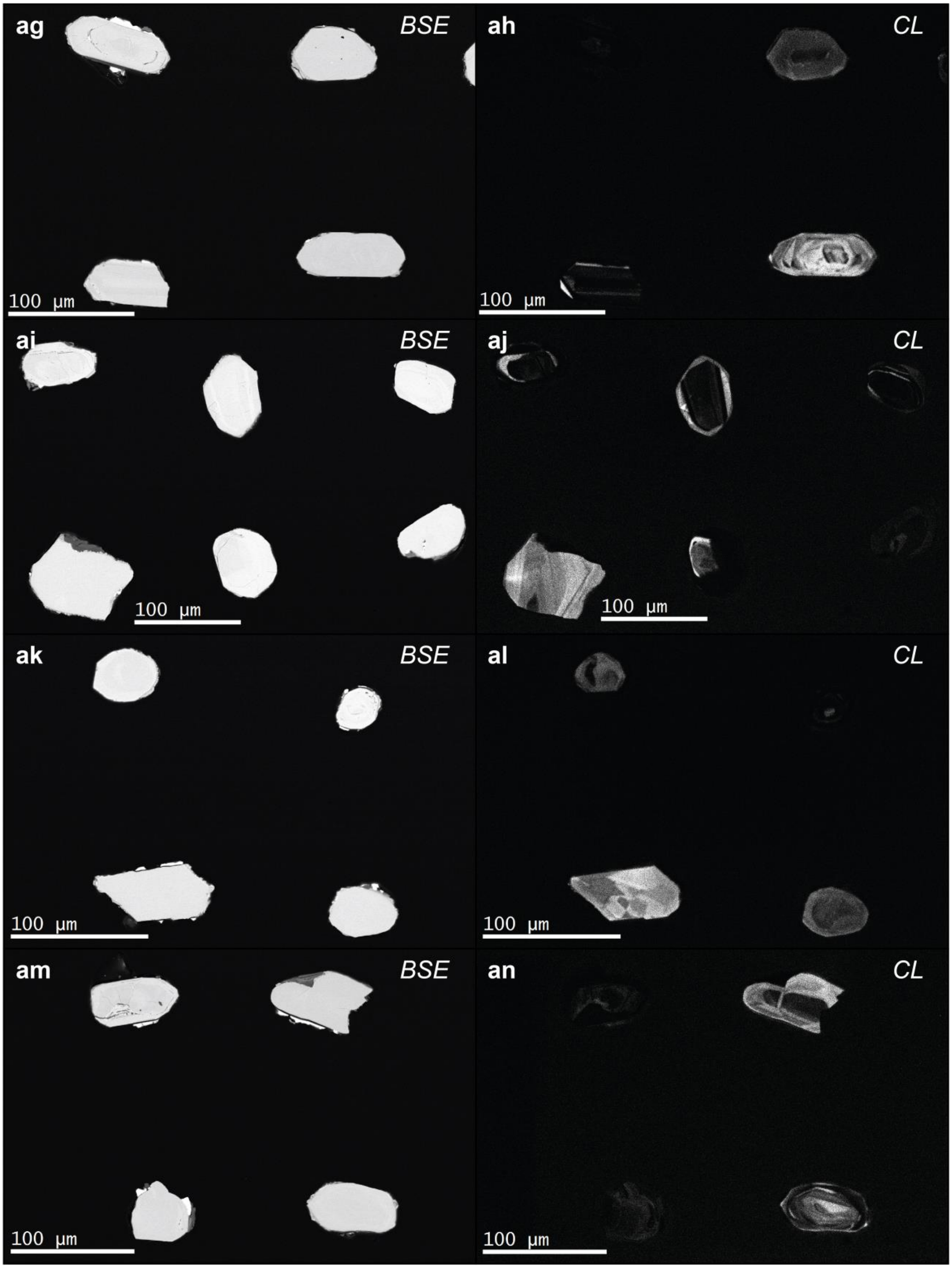

Supplementary Fig. 1 continued.

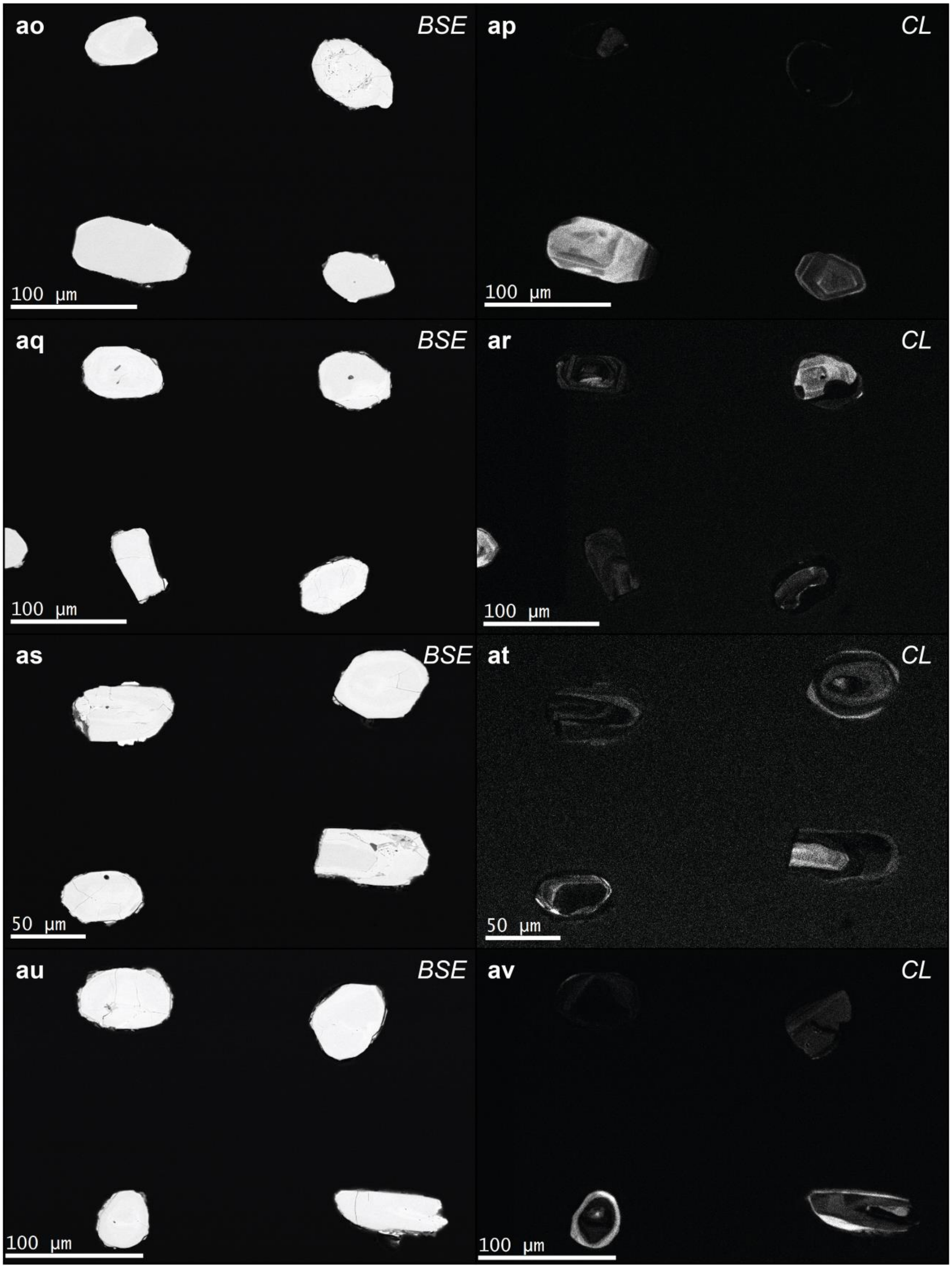

Supplementary Fig. 1 continued.

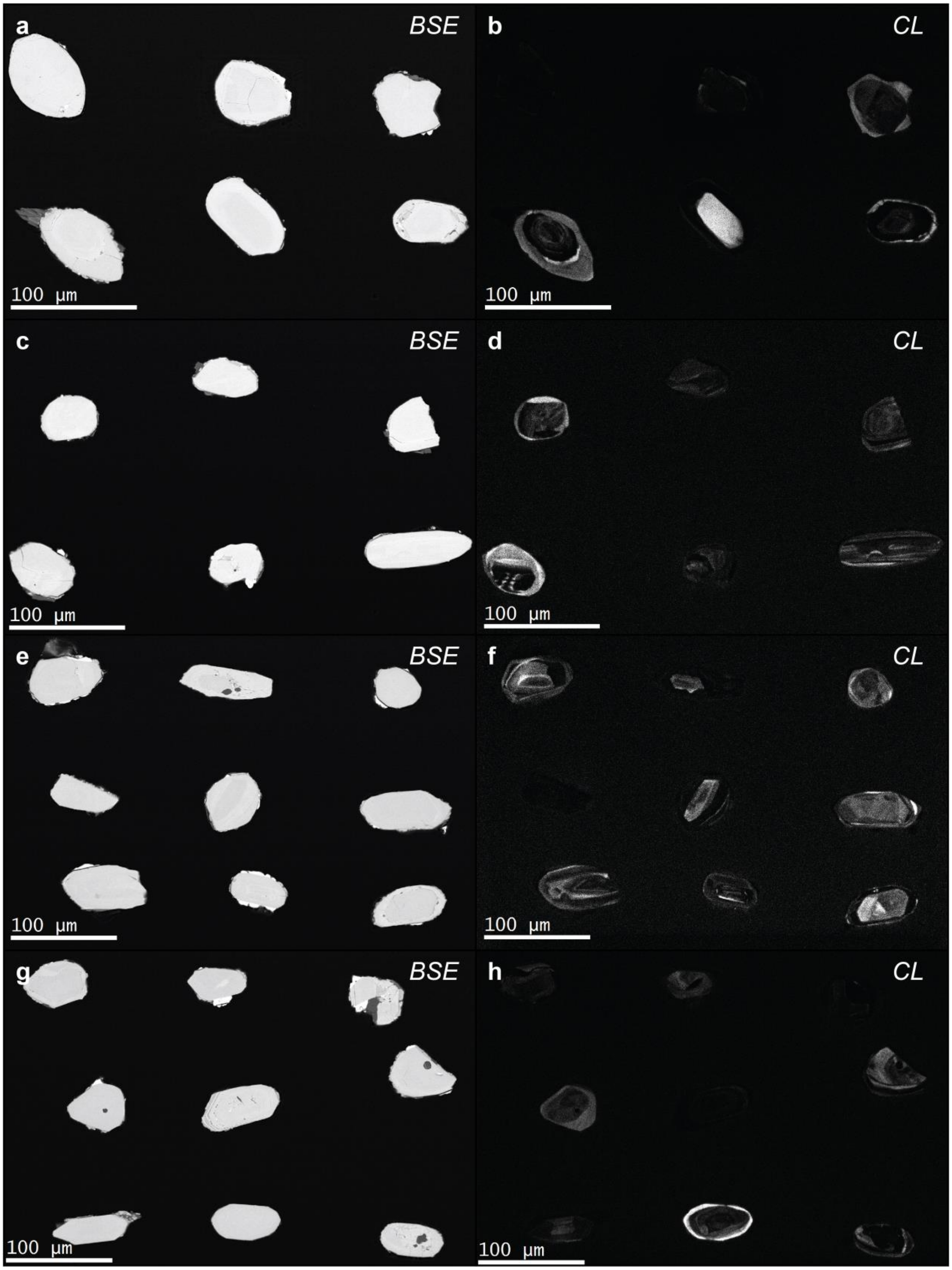

Supplementary Fig. 2. Backscattered electron (BSE), left panel, and cathodoluminescence (CL), right panel, imaging of zircon in magnetic-at-1.7 A fraction of control sample YPAcon1 (rock).

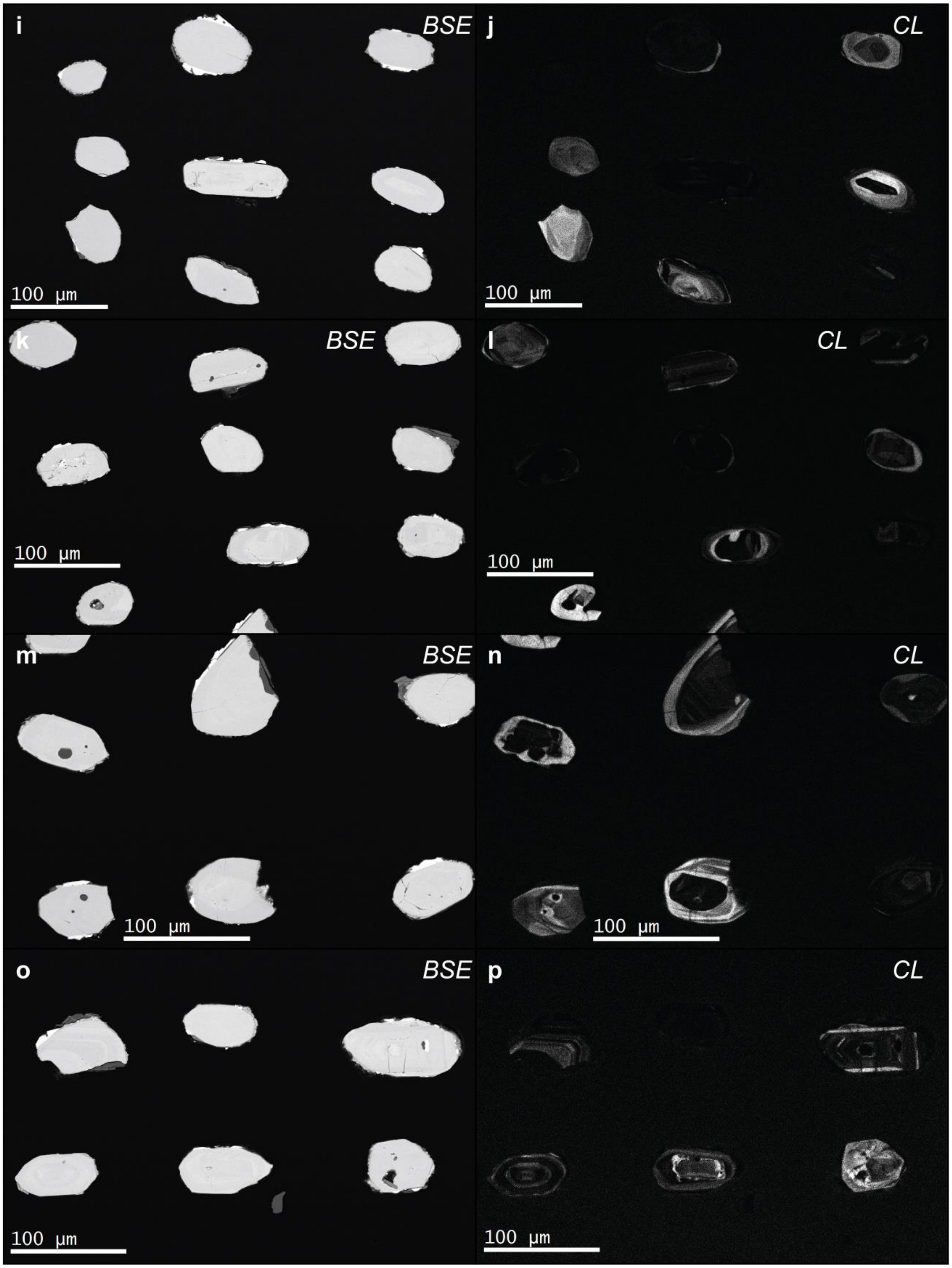

Supplementary Fig. 2 continued.

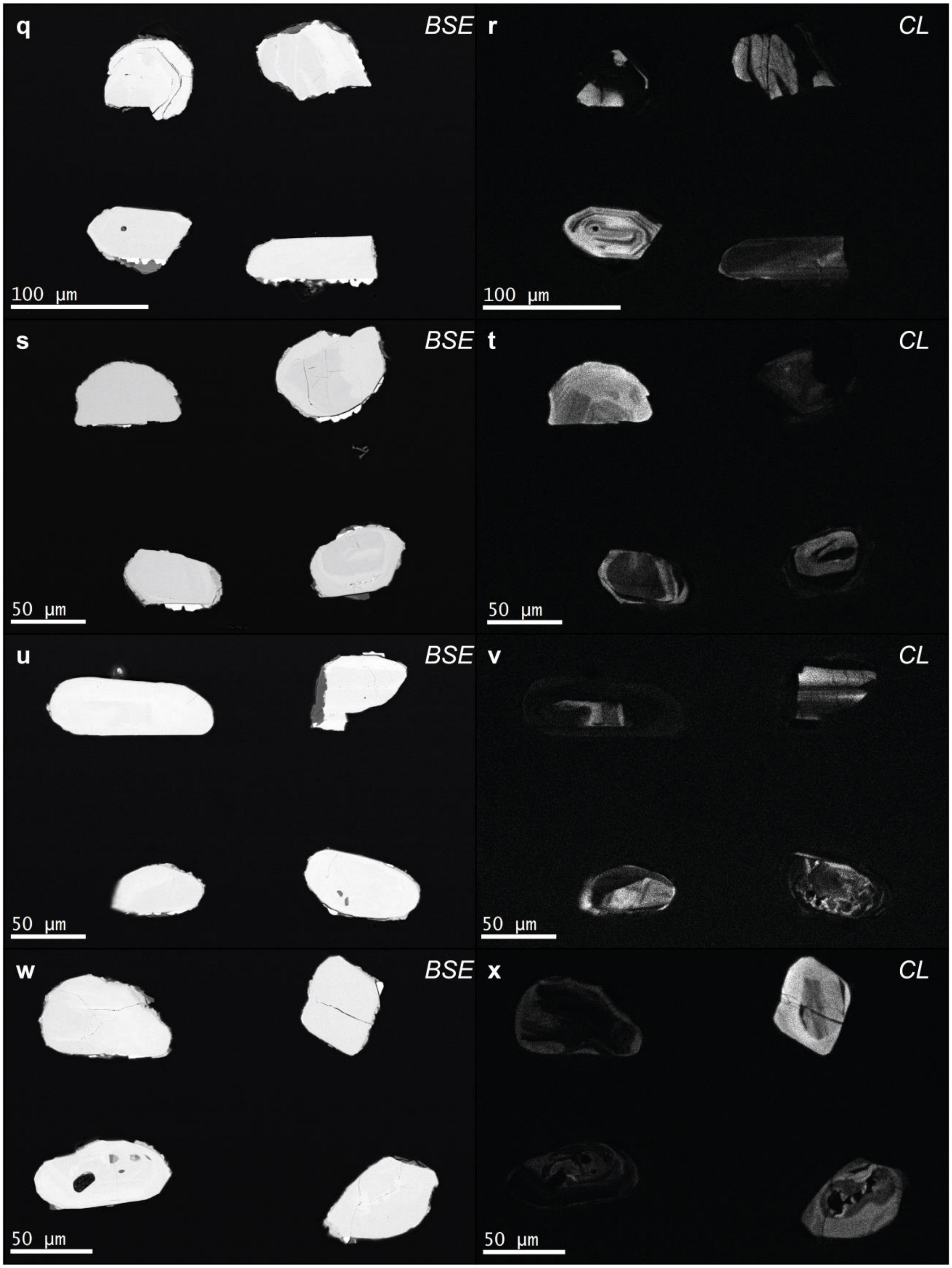

Supplementary Fig. 2 continued.

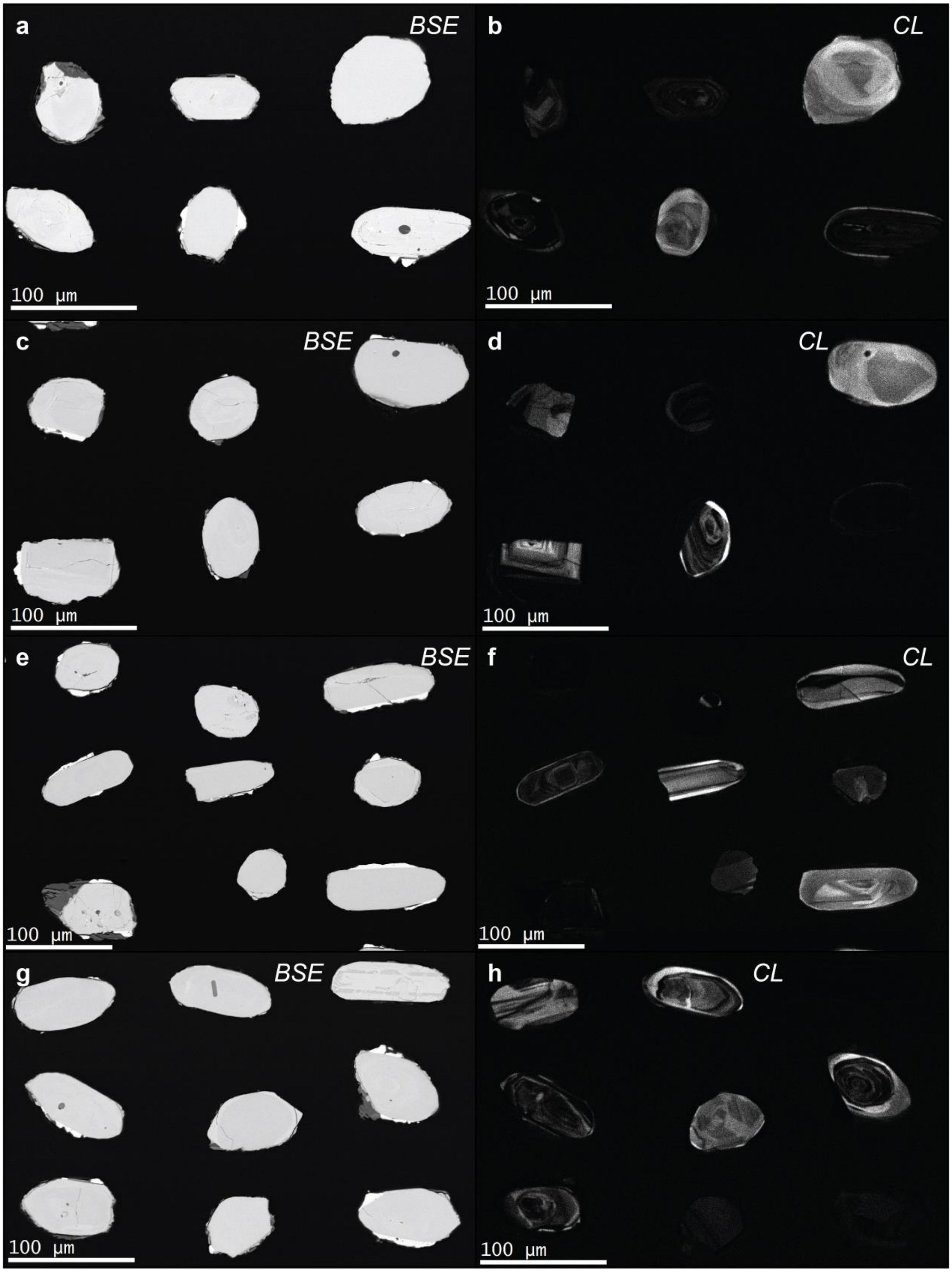

Supplementary Fig. 3. Backscattered electron (BSE), left panel, and cathodoluminescence (CL), right panel, imaging of zircon in magnetic-at-1.5 A fraction of control sample YPAcon1 (rock).

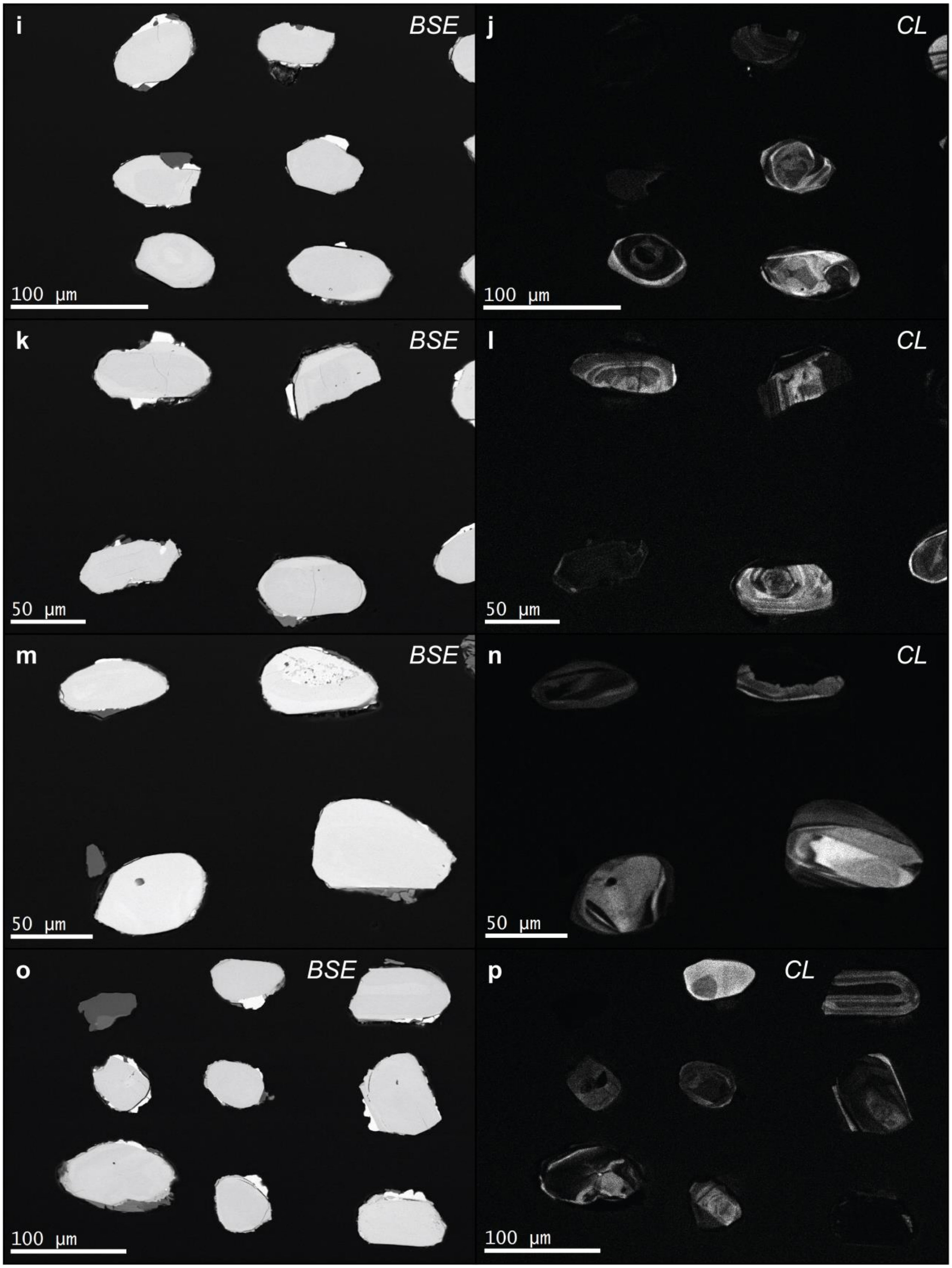

Supplementary Fig. 3 continued.

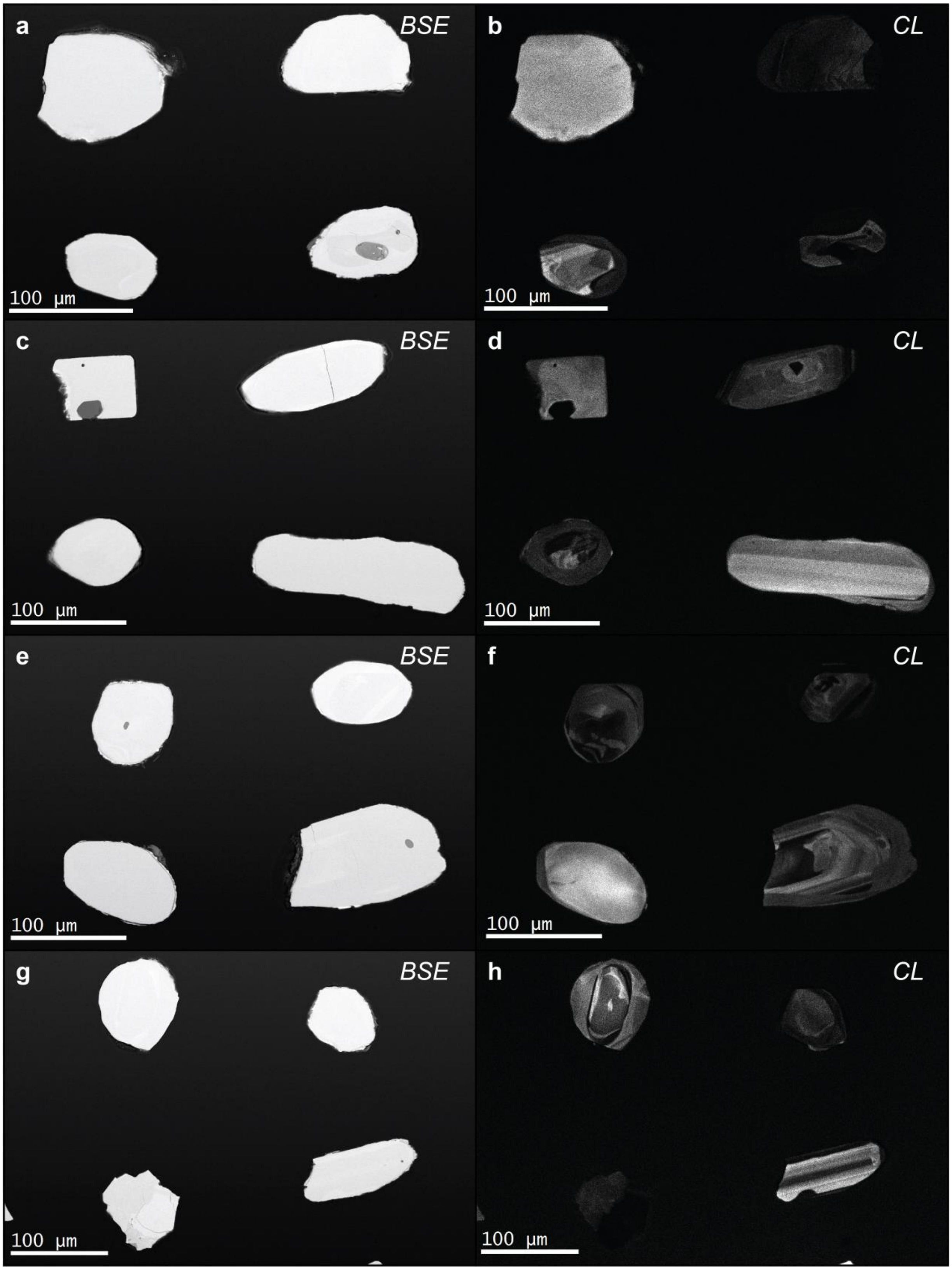

Supplementary Fig. 4. Backscattered electron (BSE), left panel, and cathodoluminescence (CL), right panel, imaging of zircon in non-magnetic-at-1.7 A fraction of control sample YPAcon3 (soil).

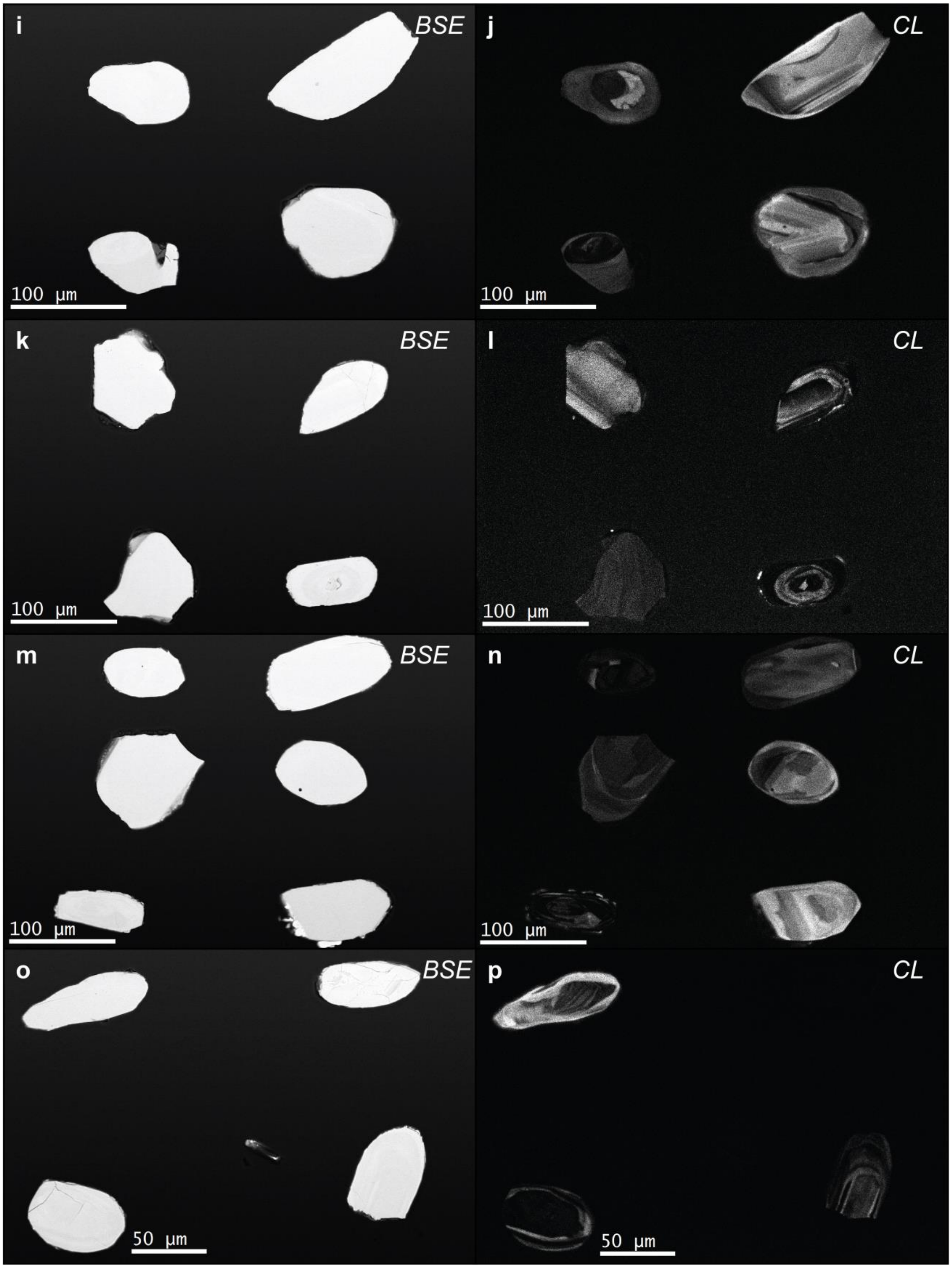

Supplementary Fig. 4 continued.

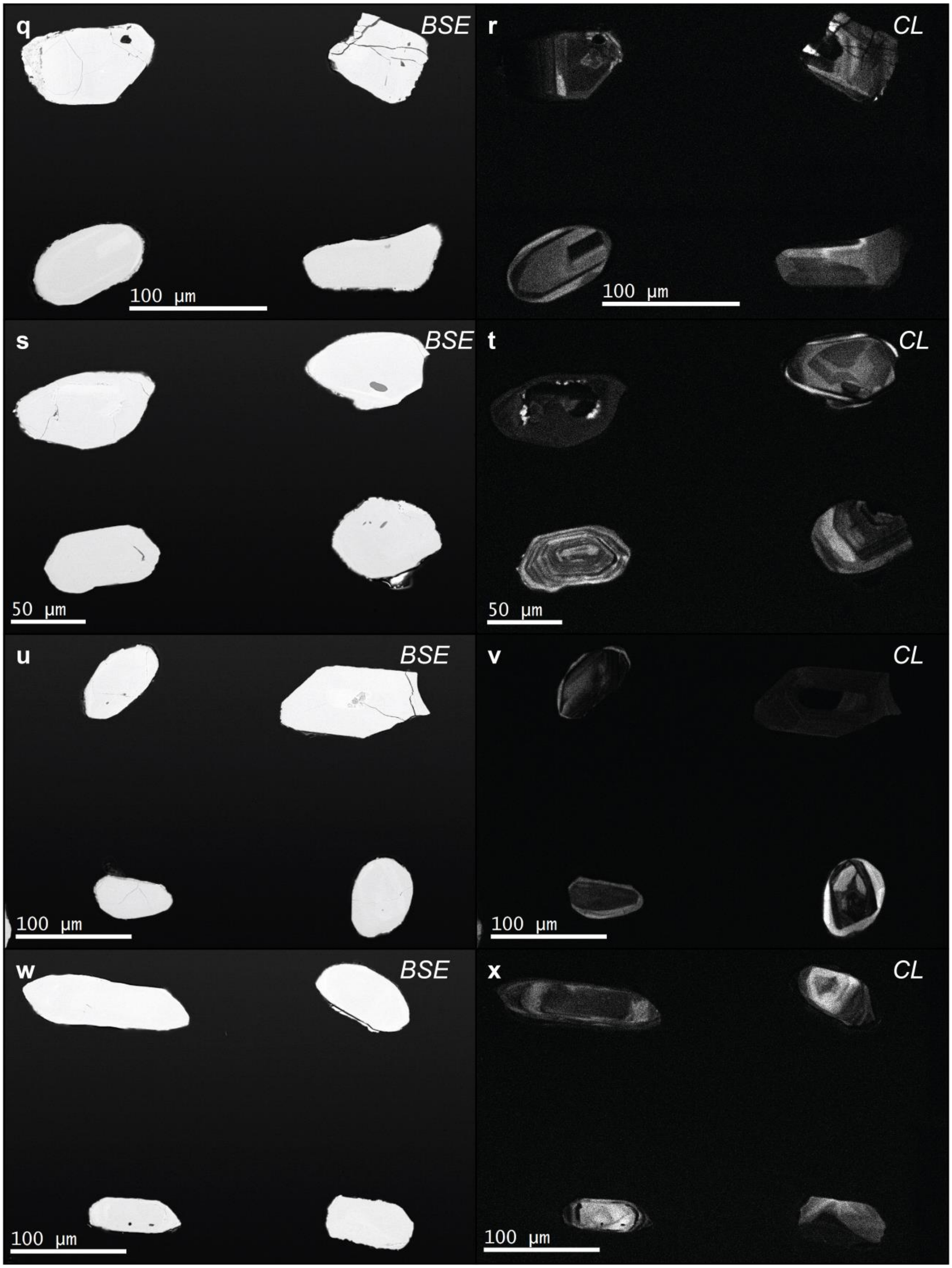

Supplementary Fig. 4 continued.

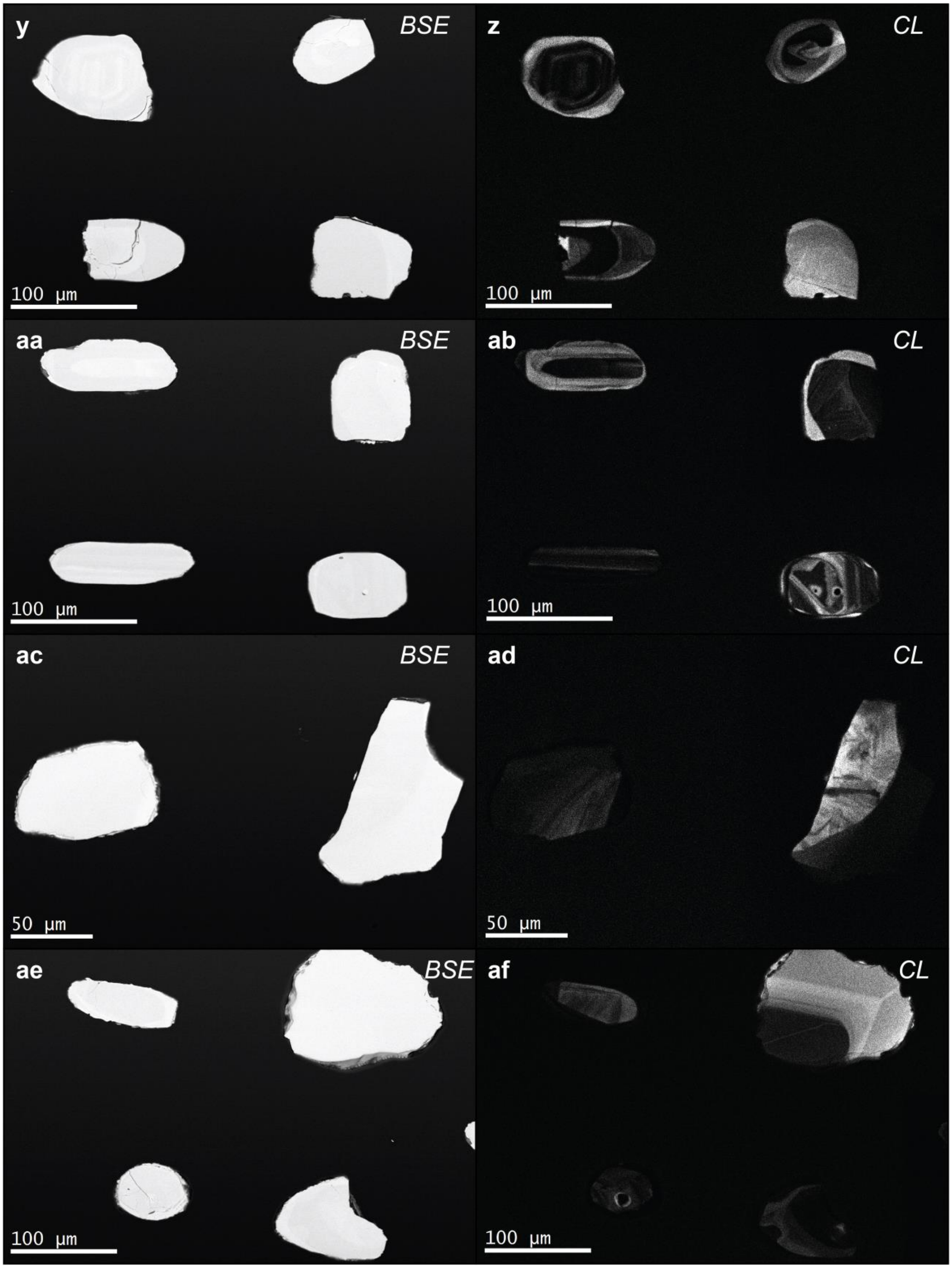

Supplementary Fig. 4 continued.

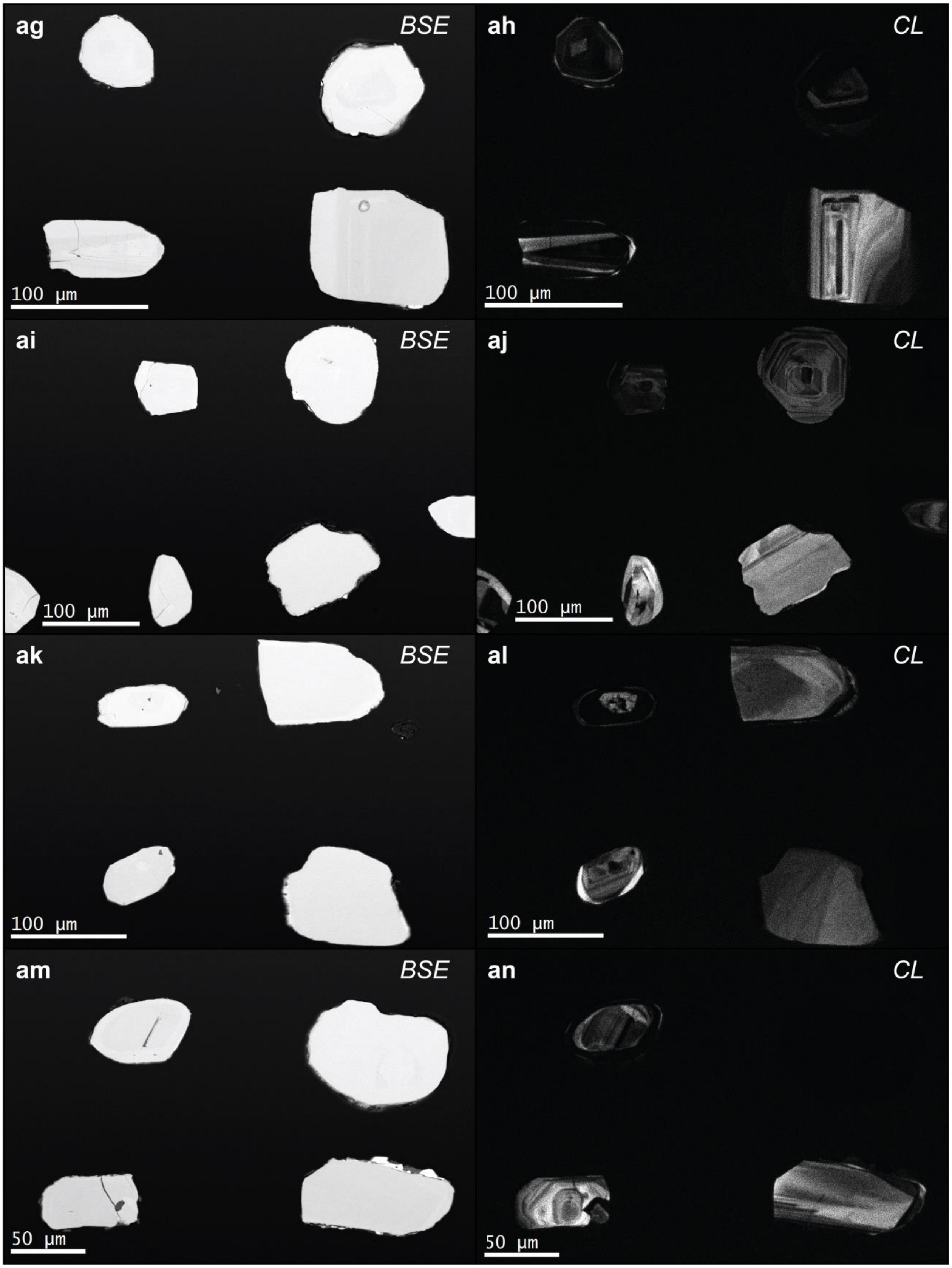

Supplementary Fig. 4 continued.

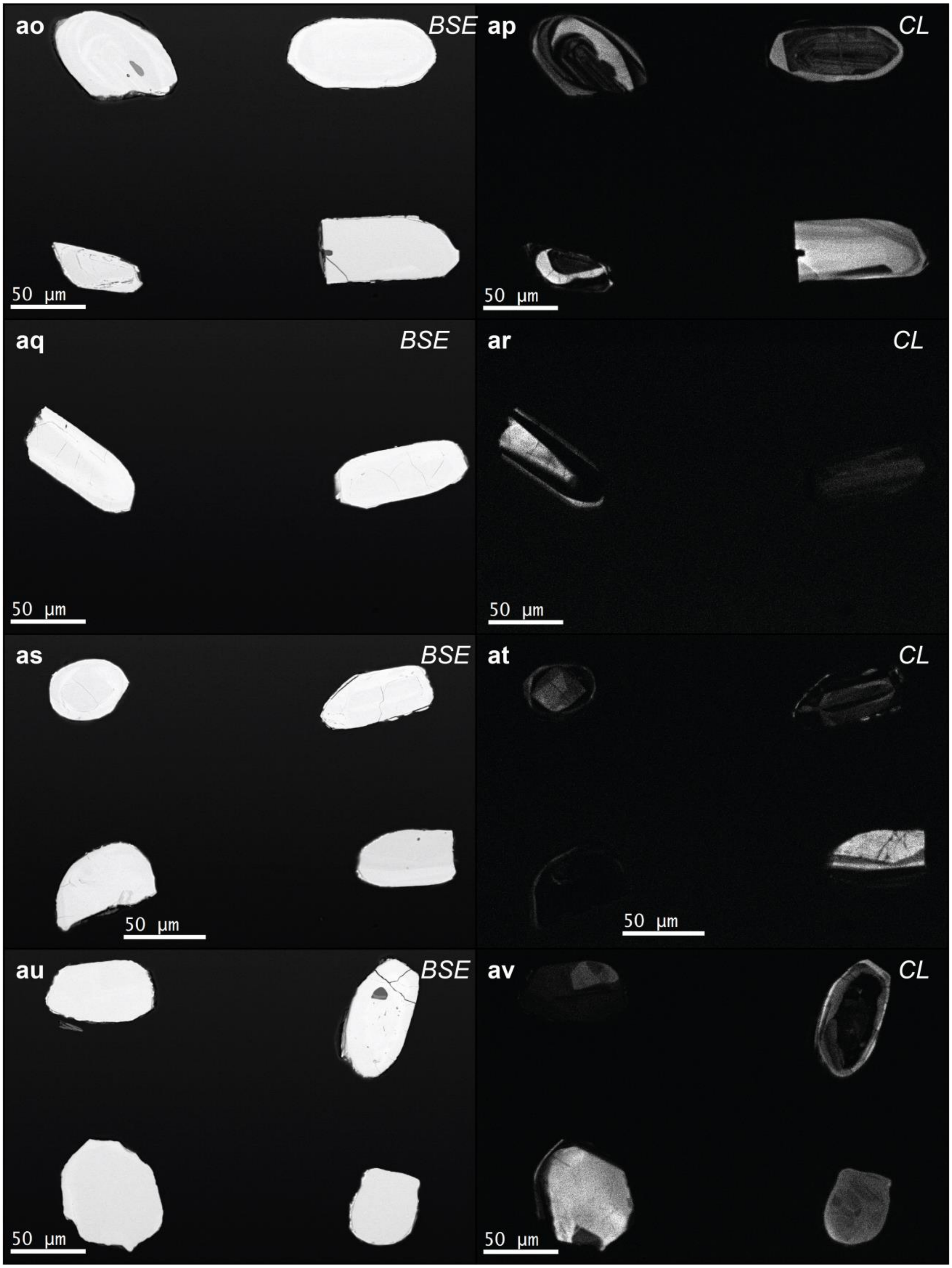

Supplementary Fig. 4 continued.

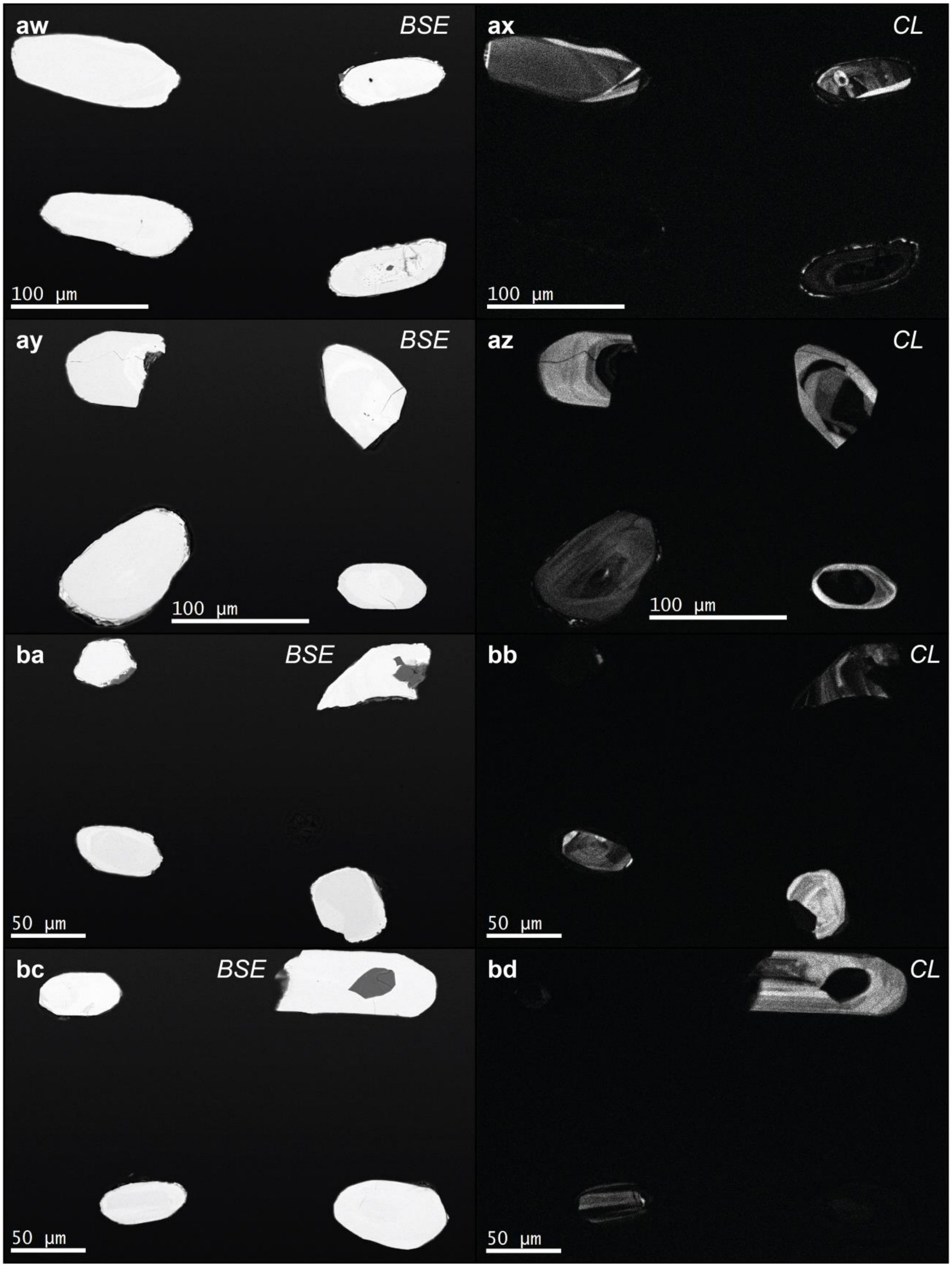

Supplementary Fig. 4 continued.

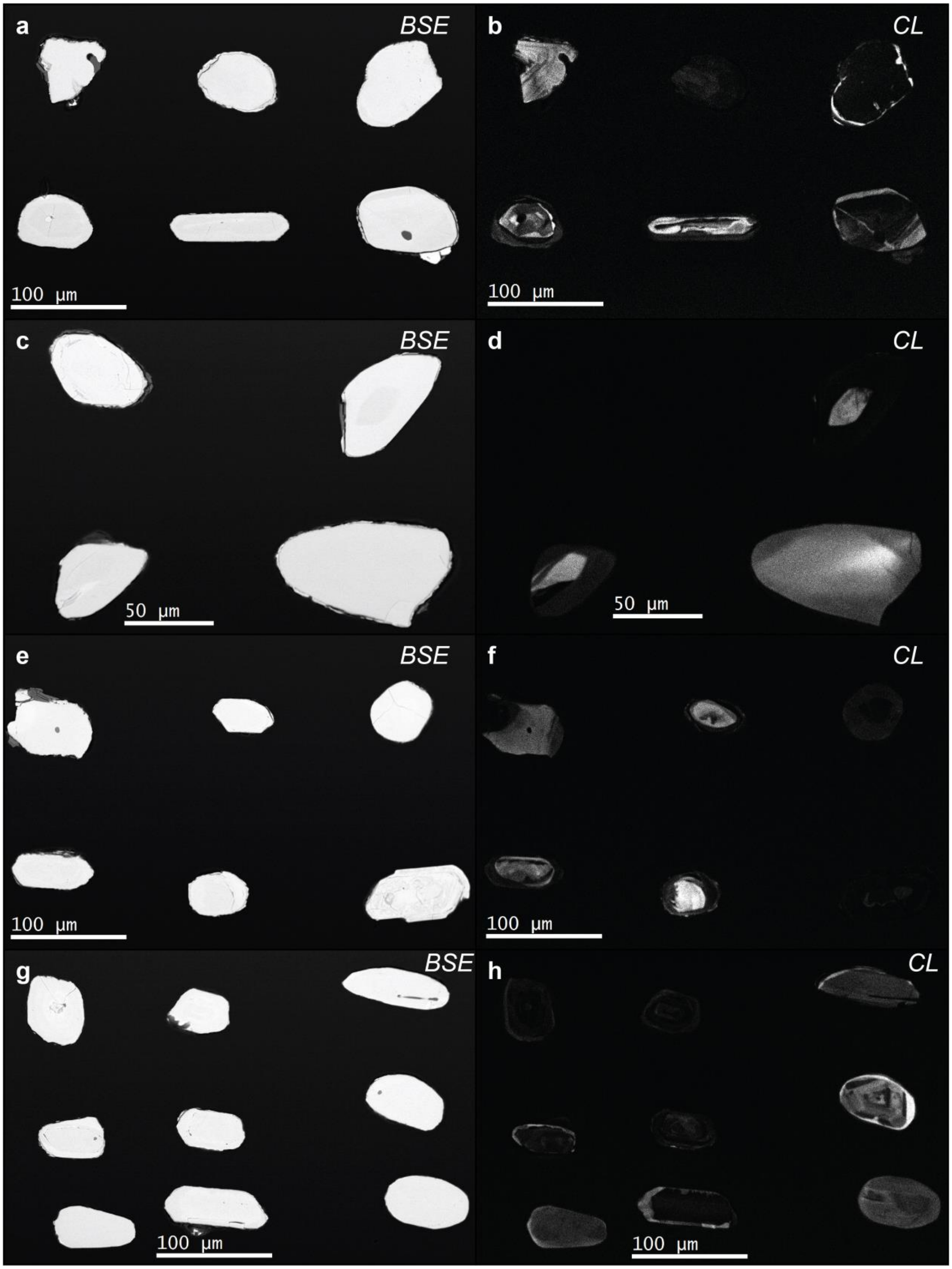

Supplementary Fig. 5. Backscattered electron (BSE), left panel, and cathodoluminescence (CL), right panel, imaging of zircon in magnetic-at-1.7 A fraction of control sample YPAcon3 (soil).

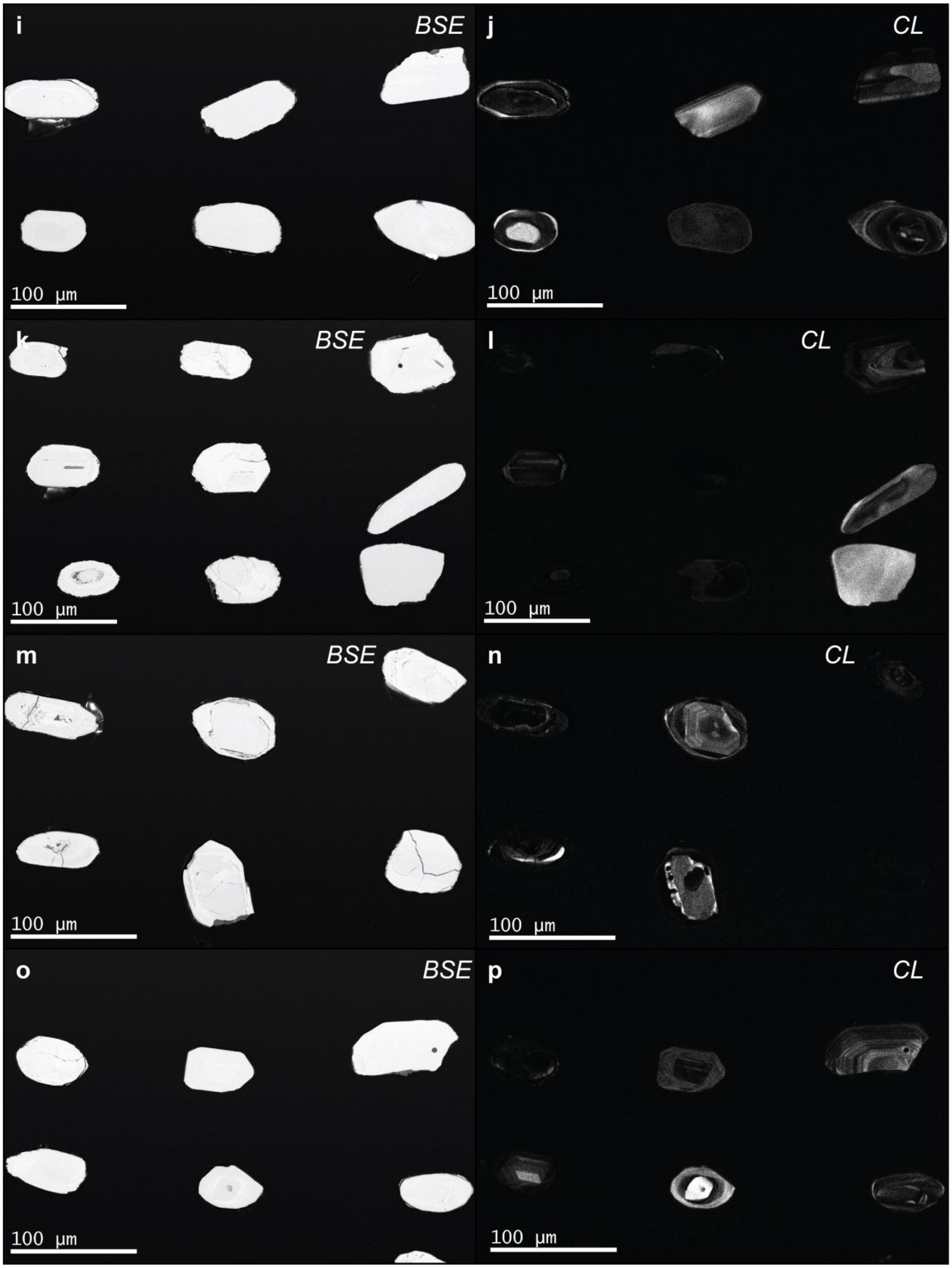

Supplementary Fig. 5 continued.

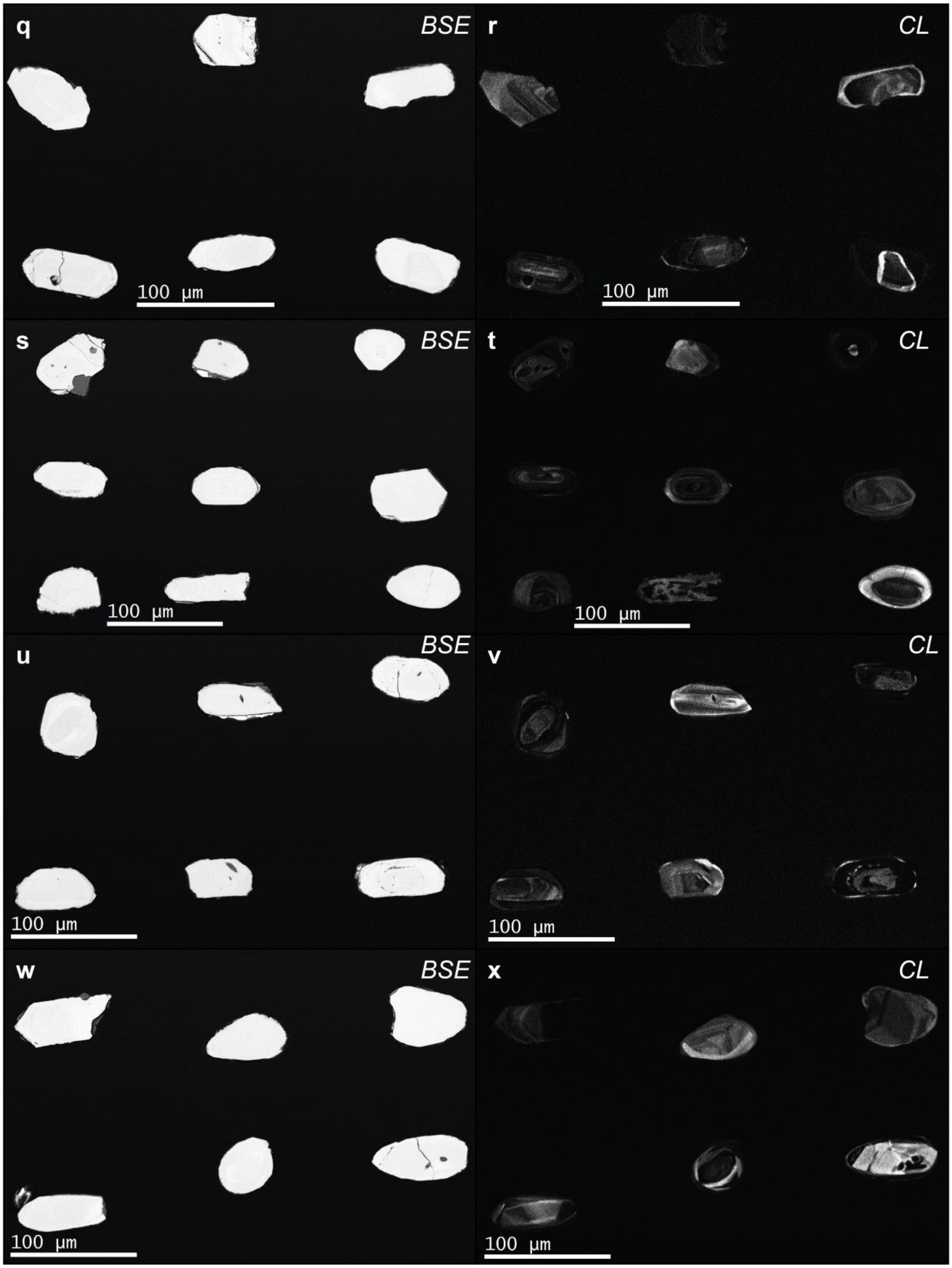

Supplementary Fig. 5 continued.

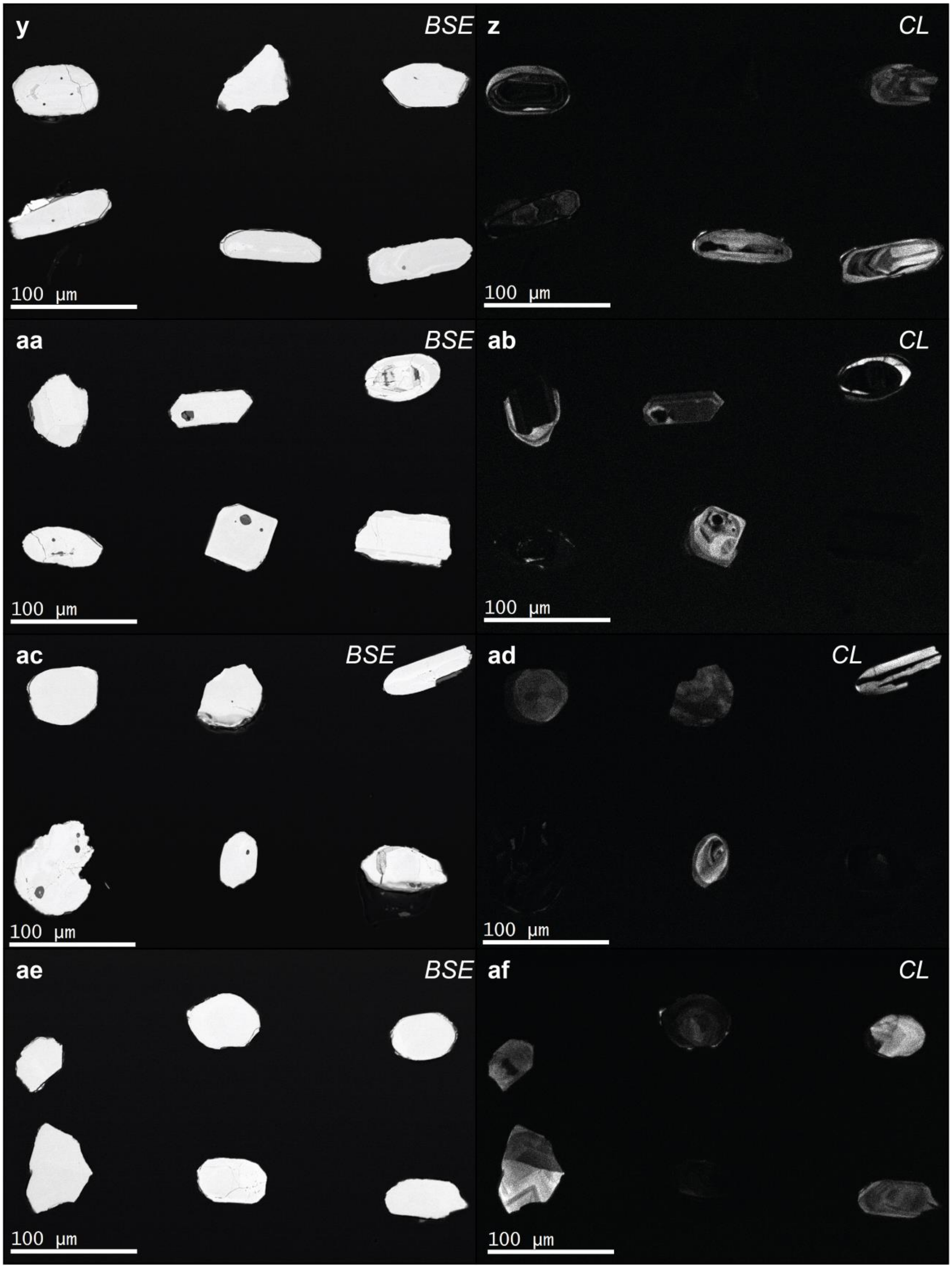

Supplementary Fig. 5 continued.

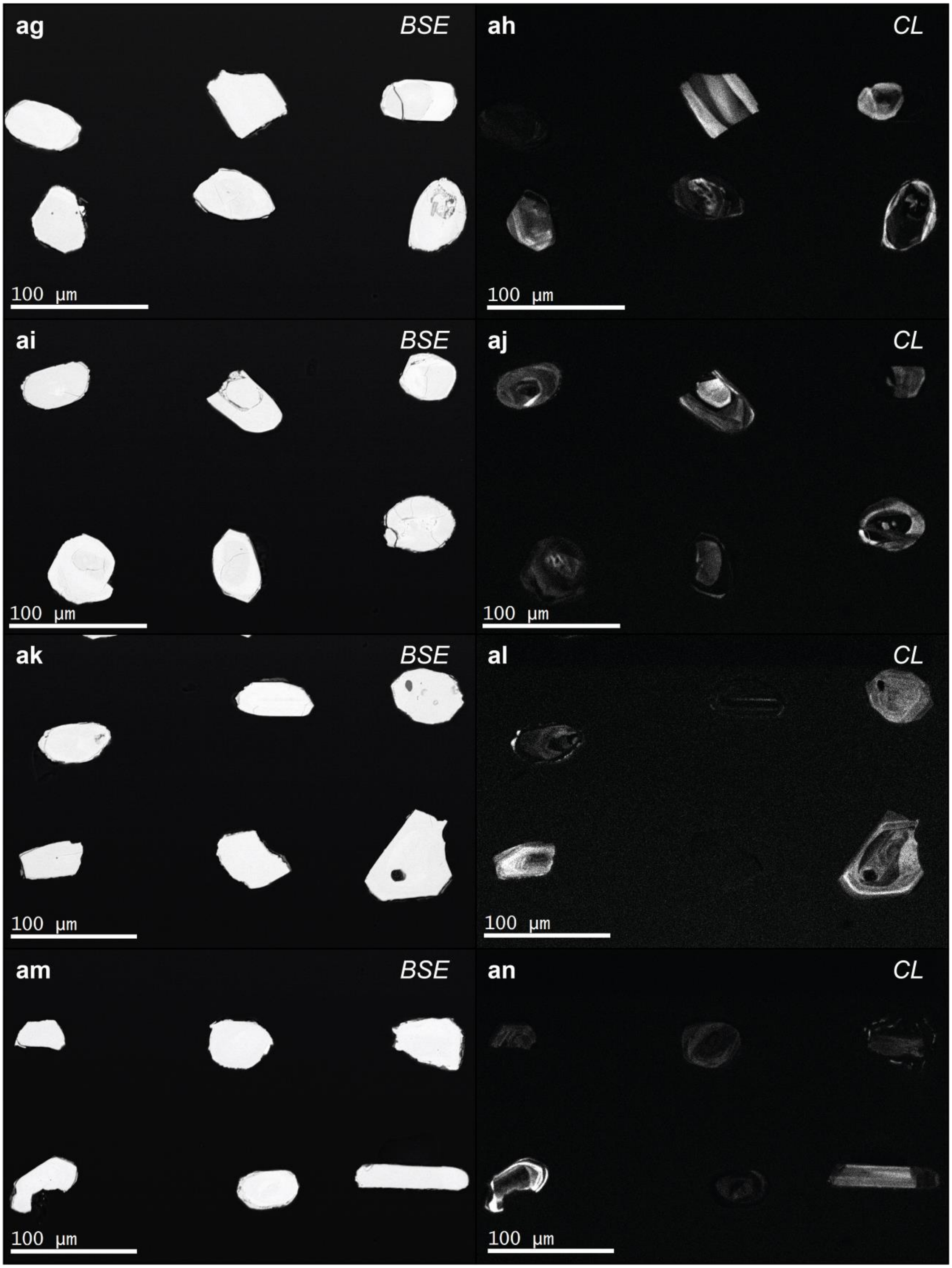

Supplementary Fig. 5 continued.

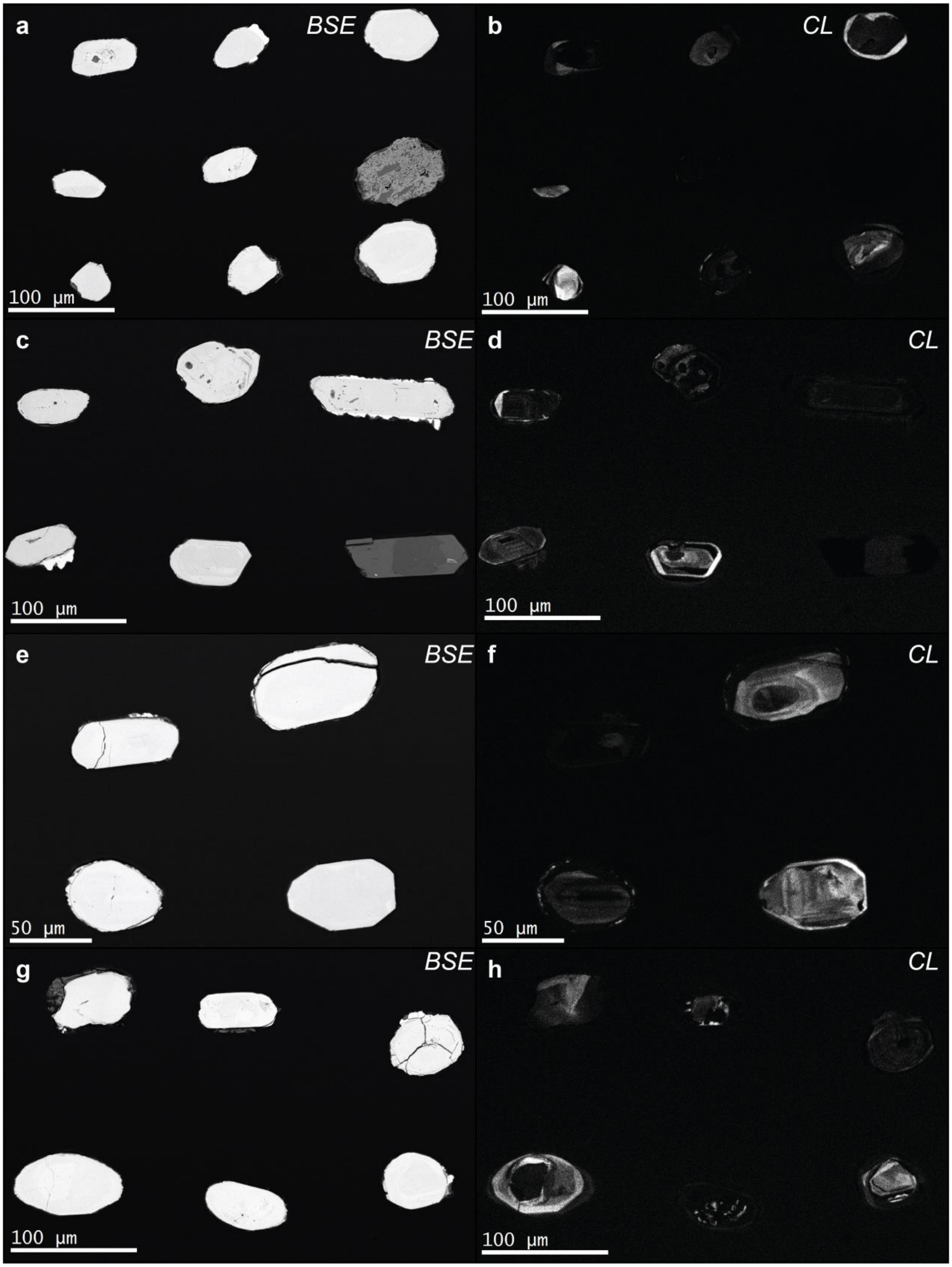

Supplementary Fig. 6. Backscattered electron (BSE), left panel, and cathodoluminescence (CL), right panel, imaging of zircon in magnetic-at-1.5 A fraction of control sample YPAcon3 (soil).

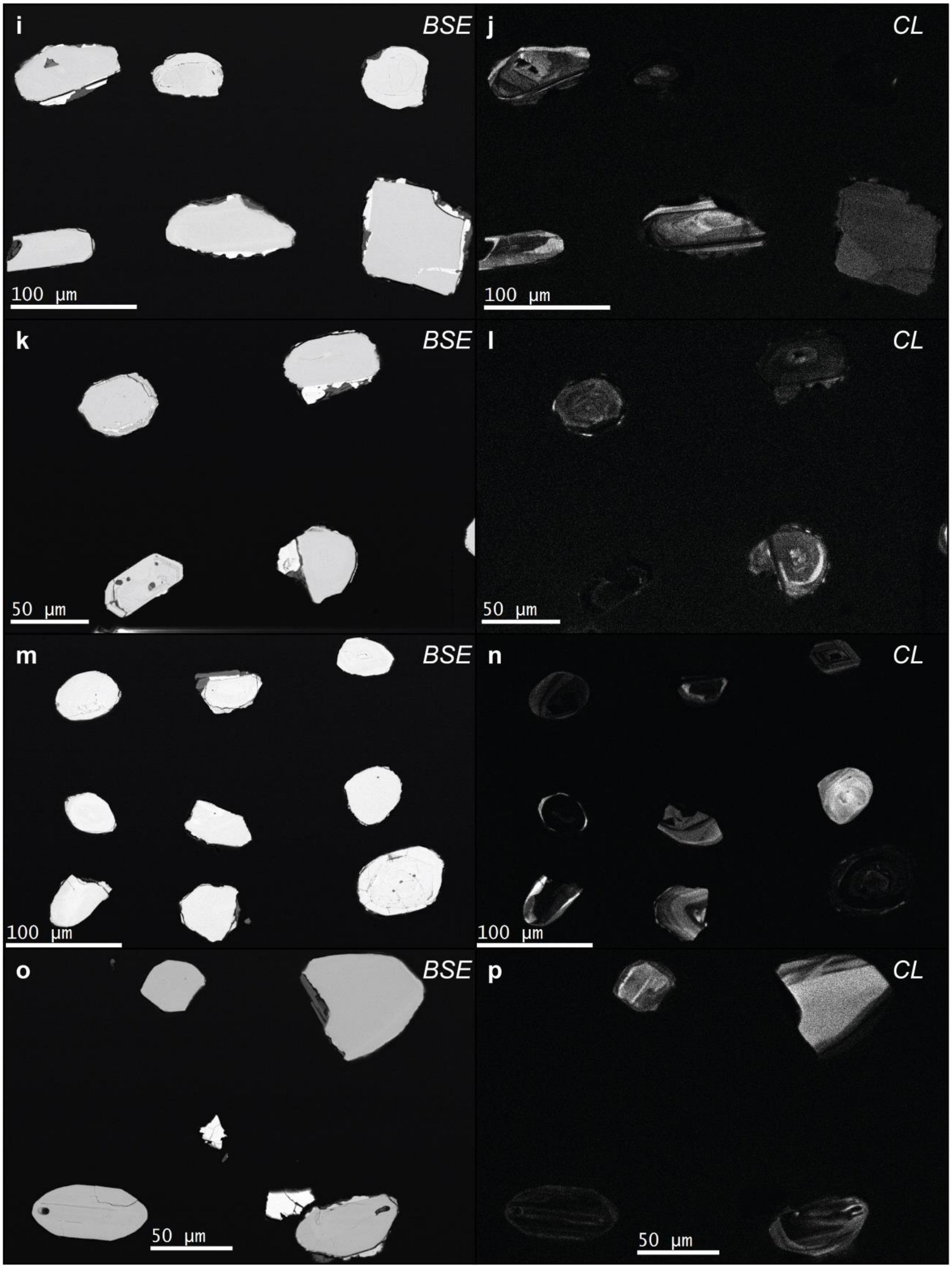

Supplementary Fig. 6 continued.

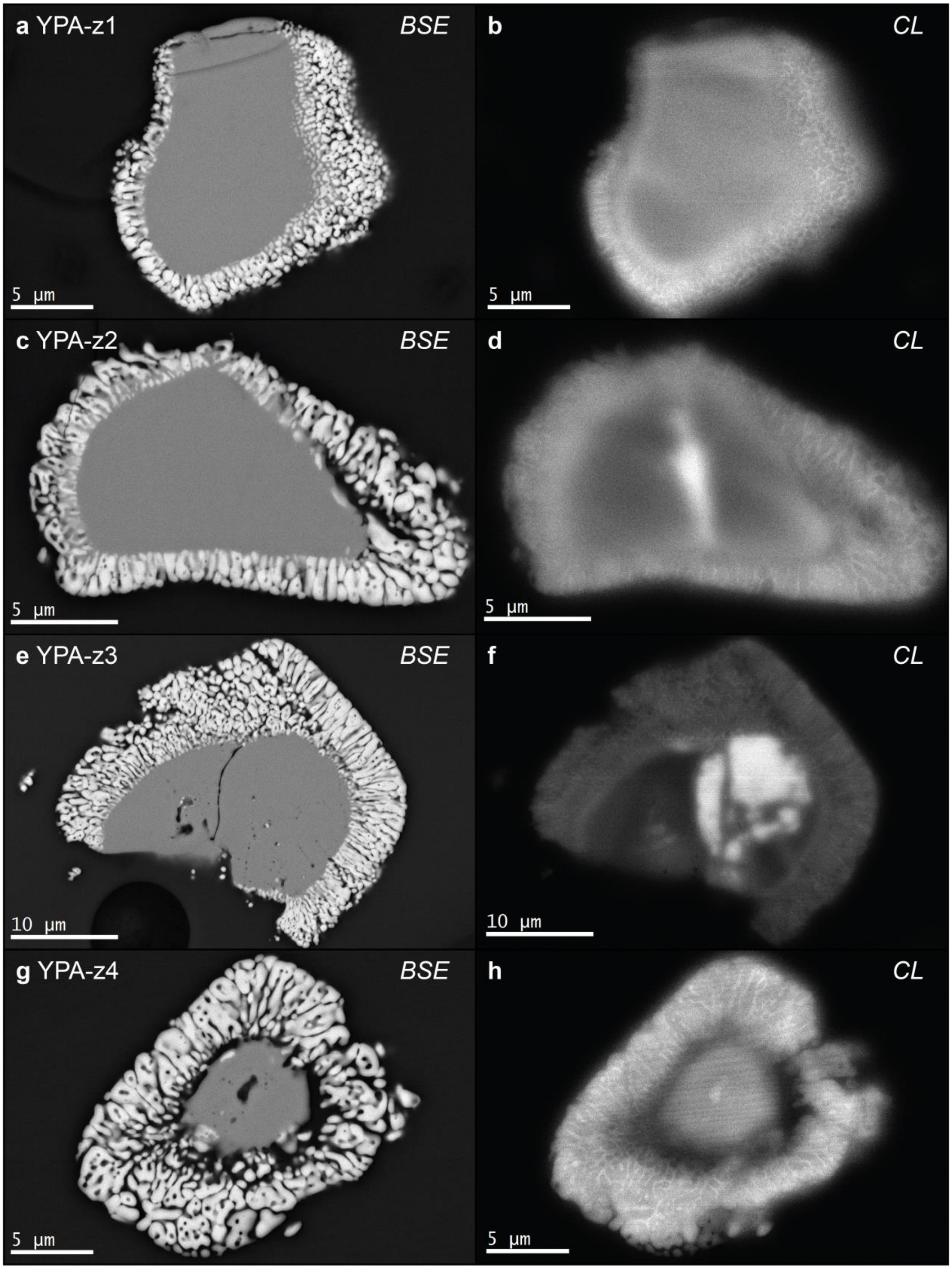

Supplementary Fig. 7. Backscattered electron (BSE), left panel, and cathodoluminescence (CL), right panel, imaging of all zircon grains from domain 1 (inner glass) of the York County fulgurite.

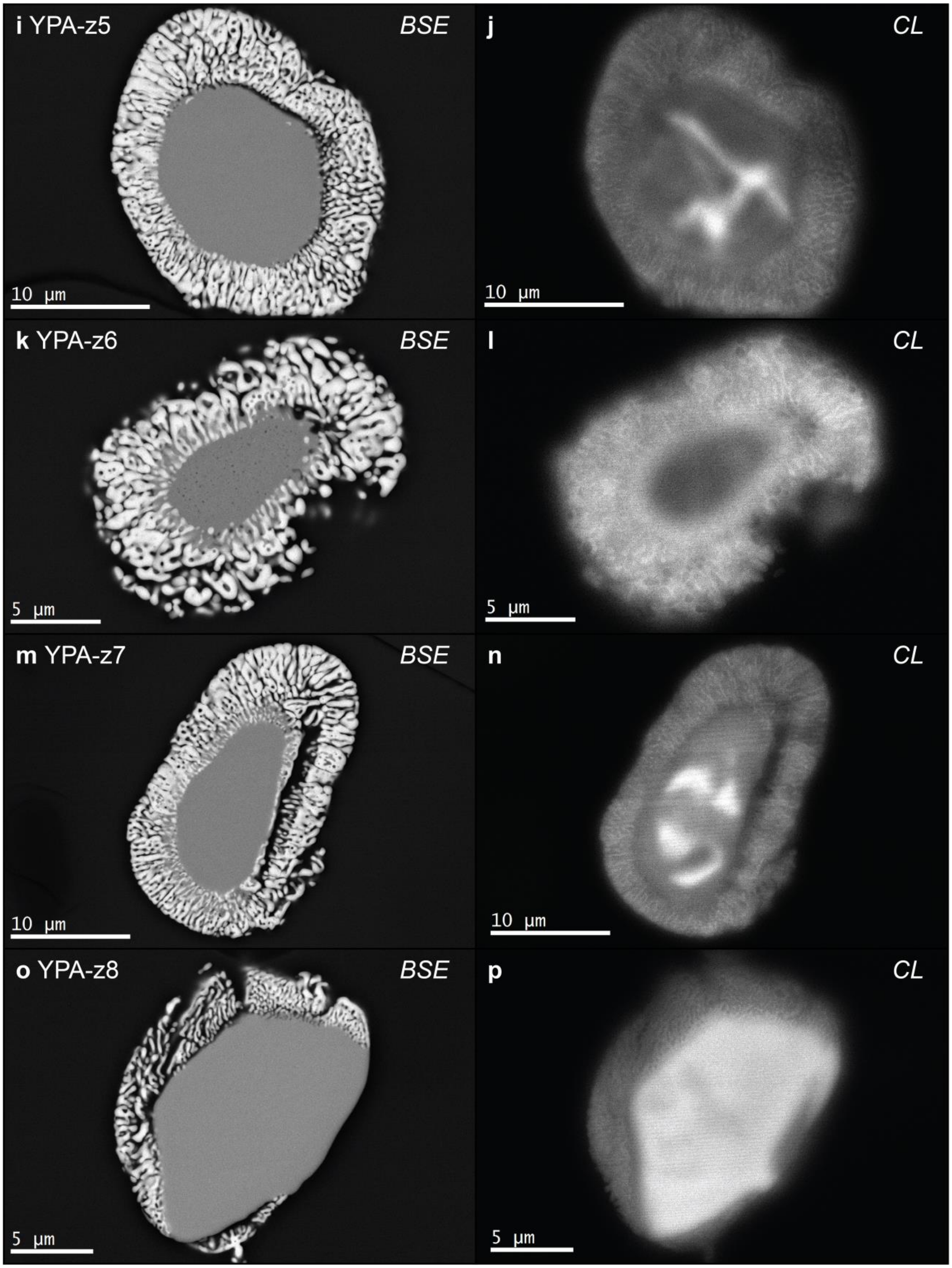

Supplementary Fig. 7 continued.

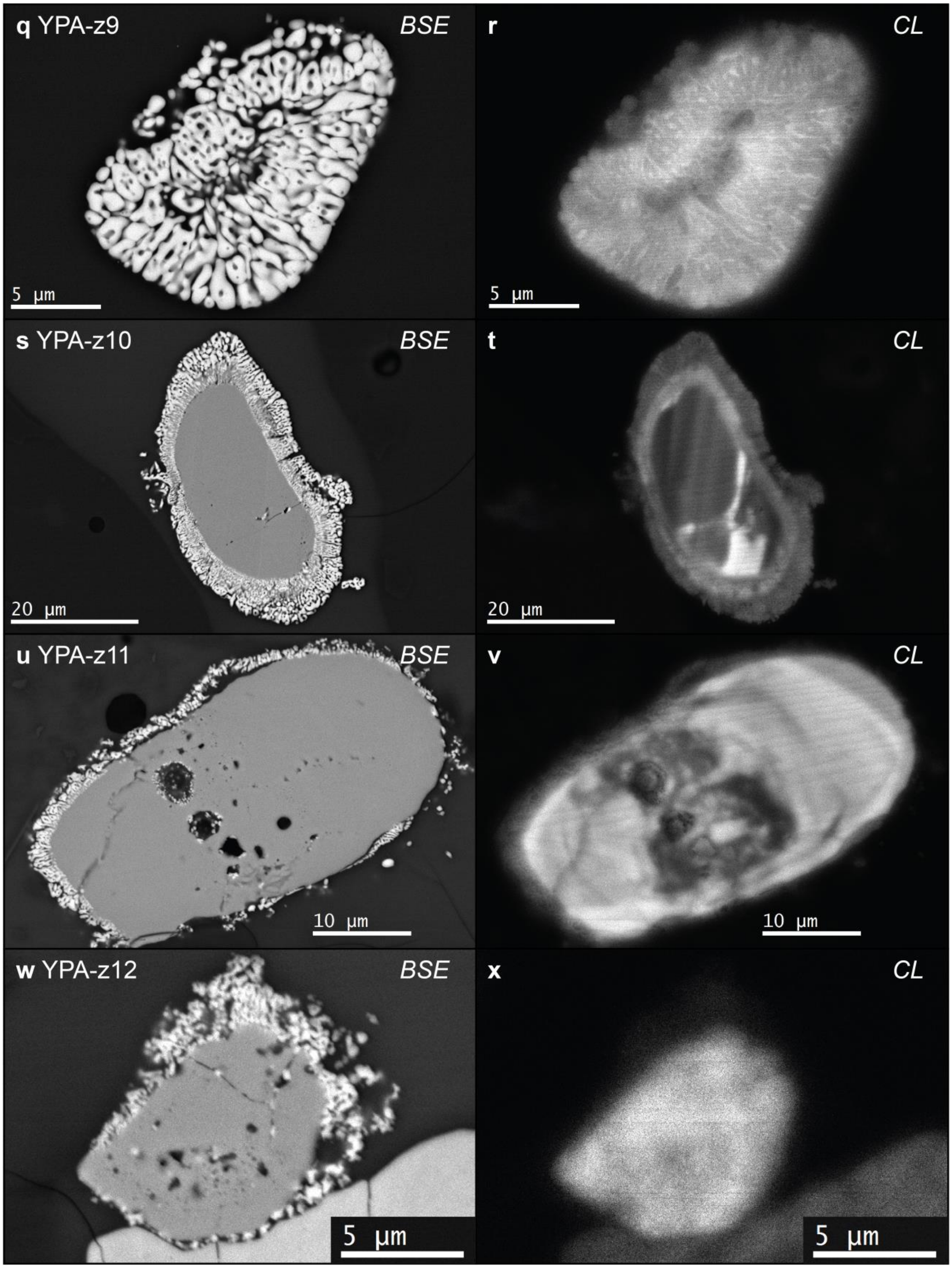

Supplementary Fig. 7 continued.

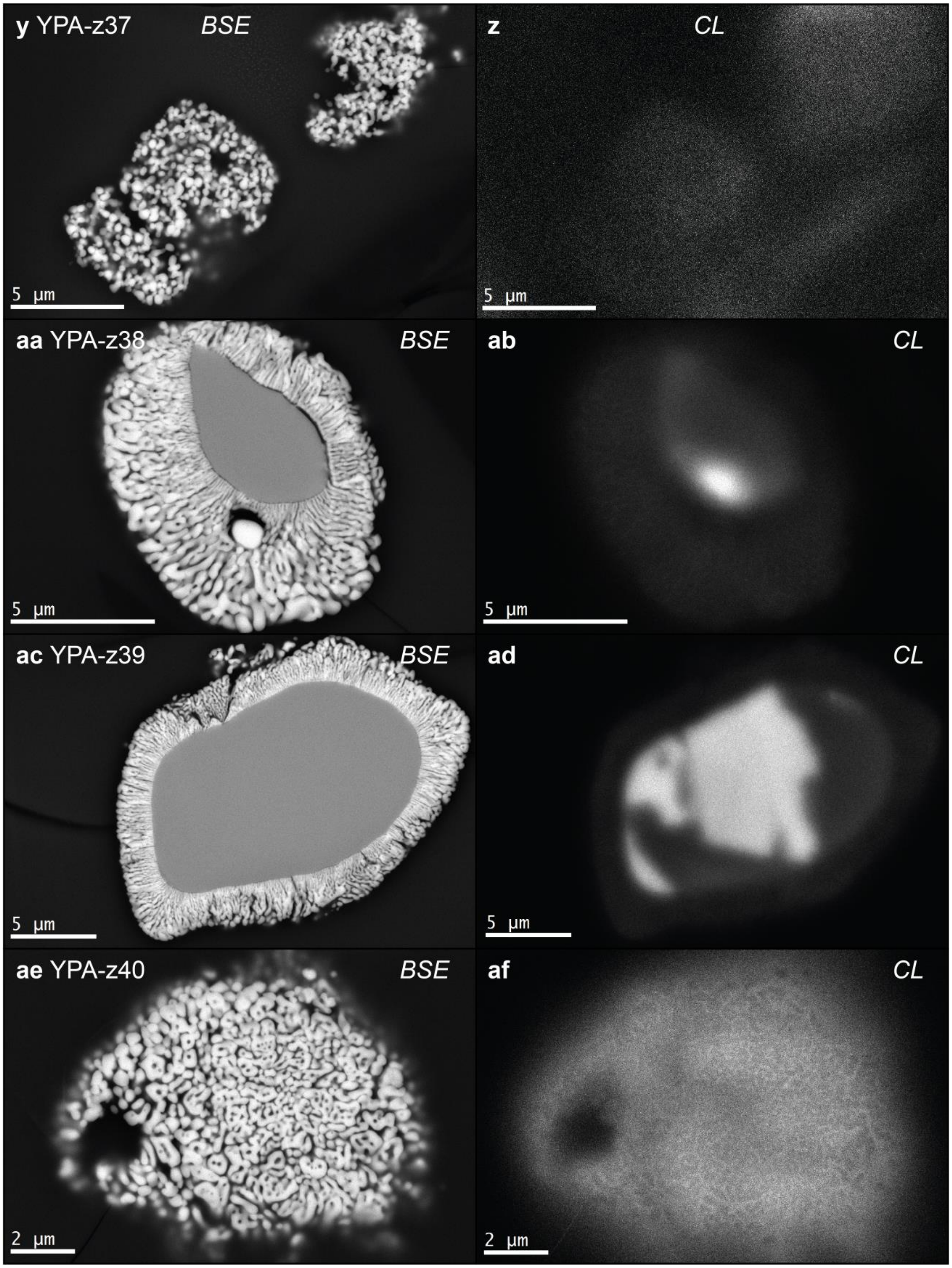

Supplementary Fig. 7 continued.

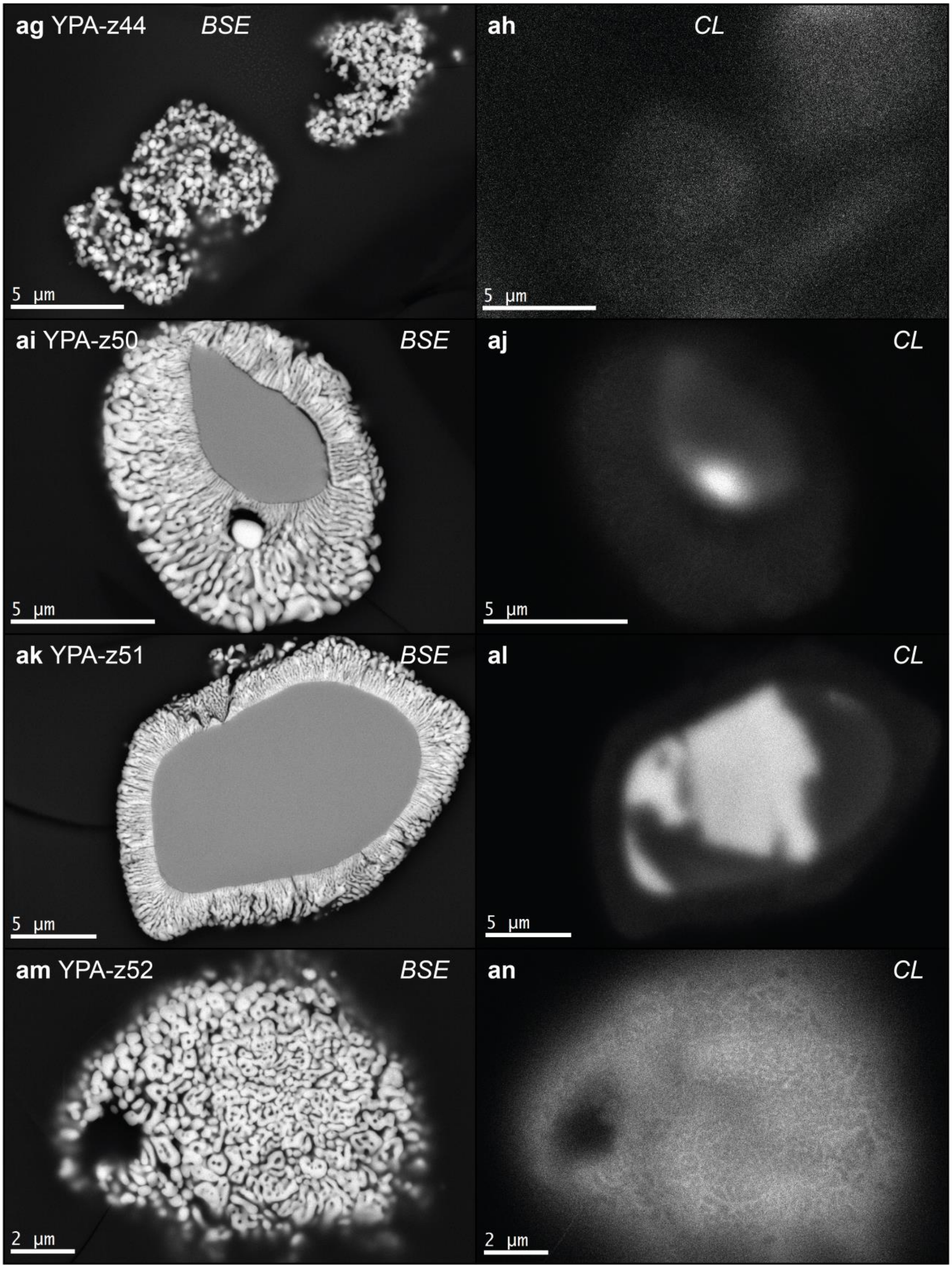

Supplementary Fig. 7 continued.

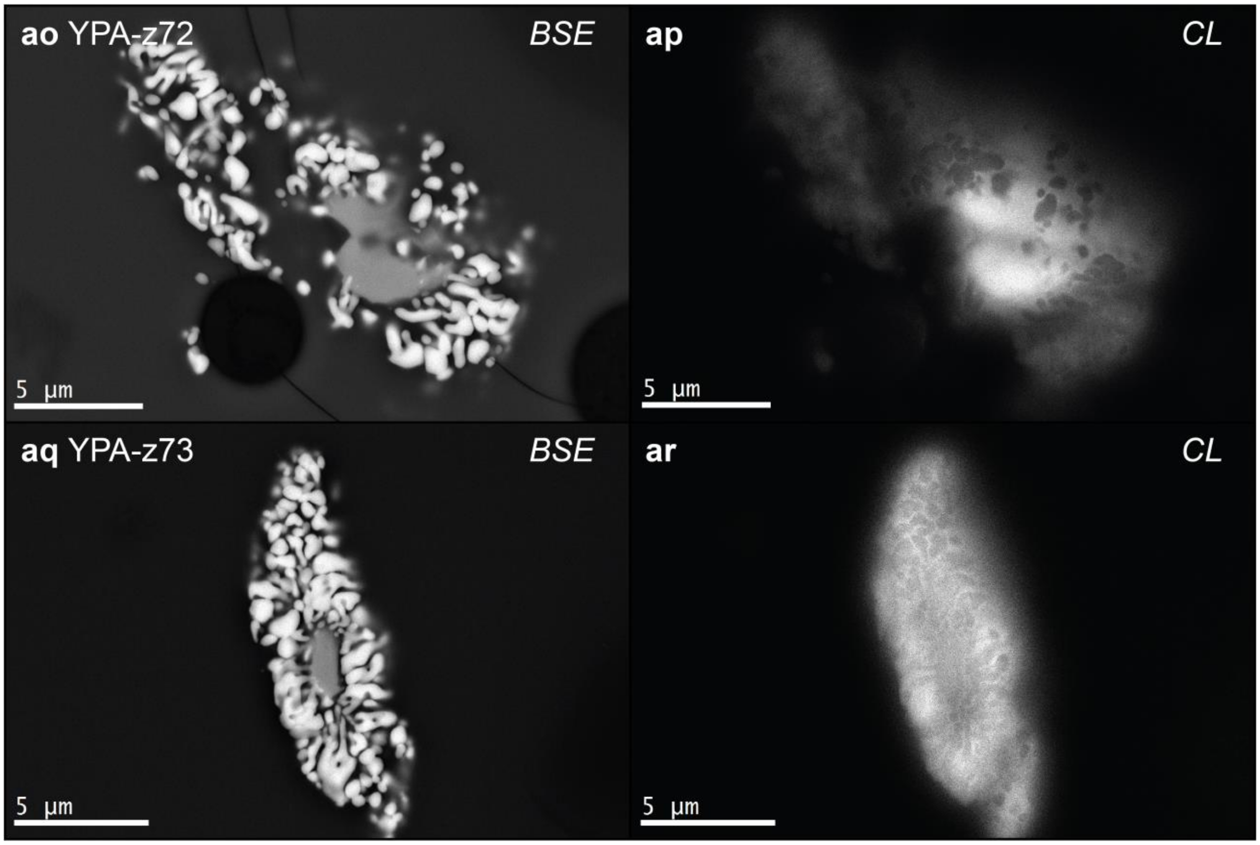

Supplementary Fig. 7 continued.

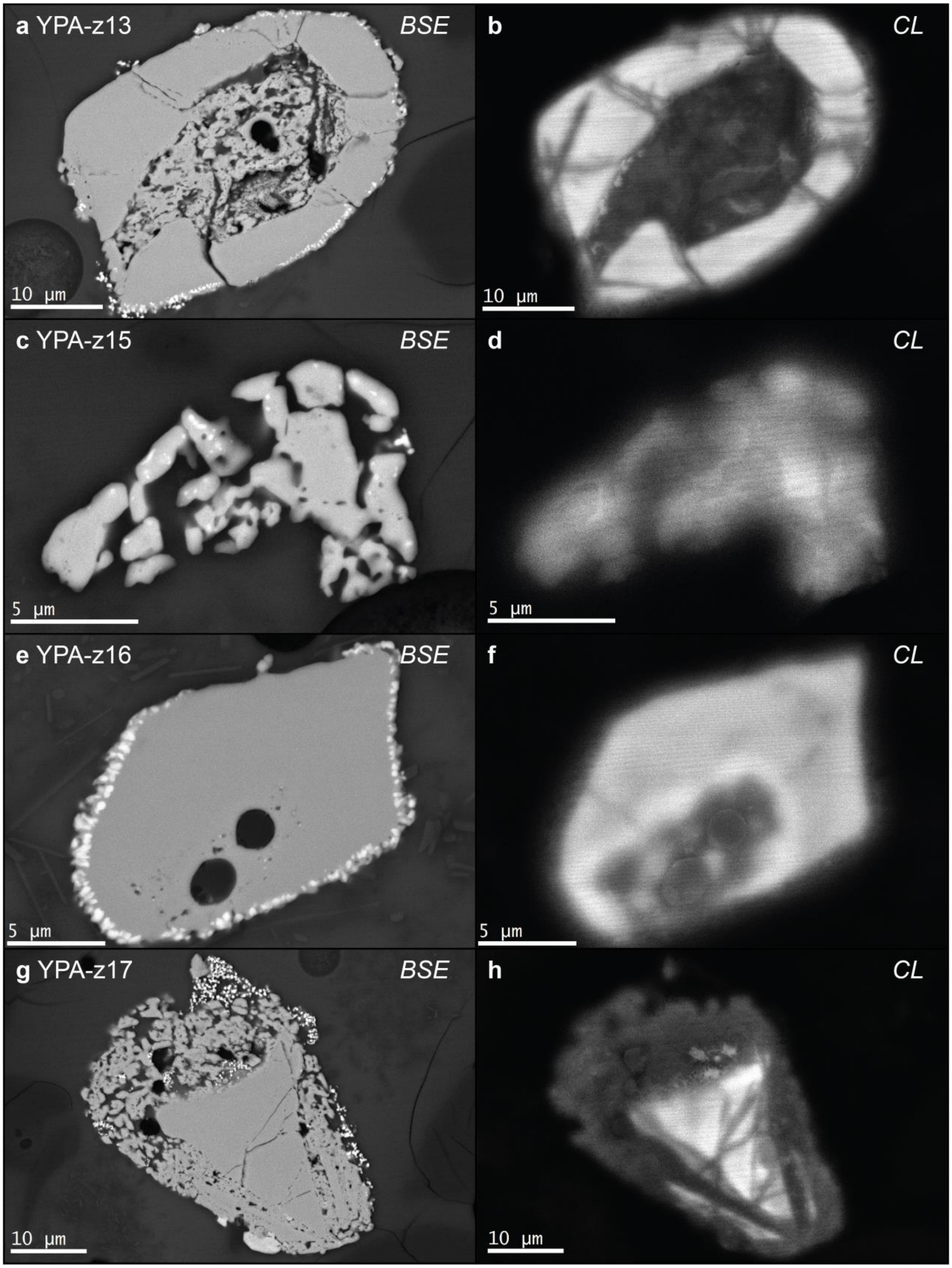

Supplementary Fig. 8. Backscattered electron (BSE), left panel, and cathodoluminescence (CL), right panel, imaging of all zircon grains from domain 2 (outer glass) of the York County fulgurite.

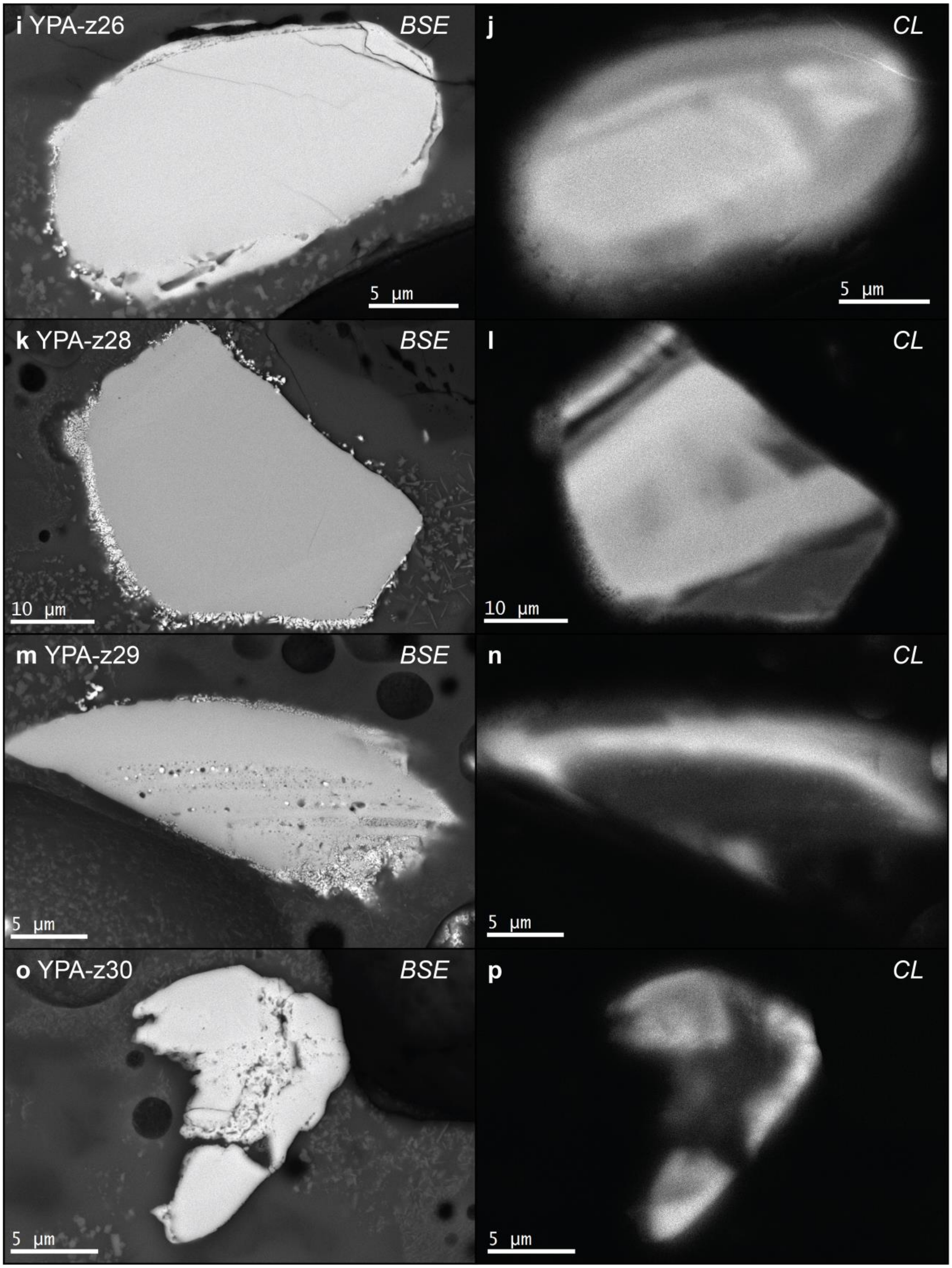

Supplementary Fig. 8 continued.

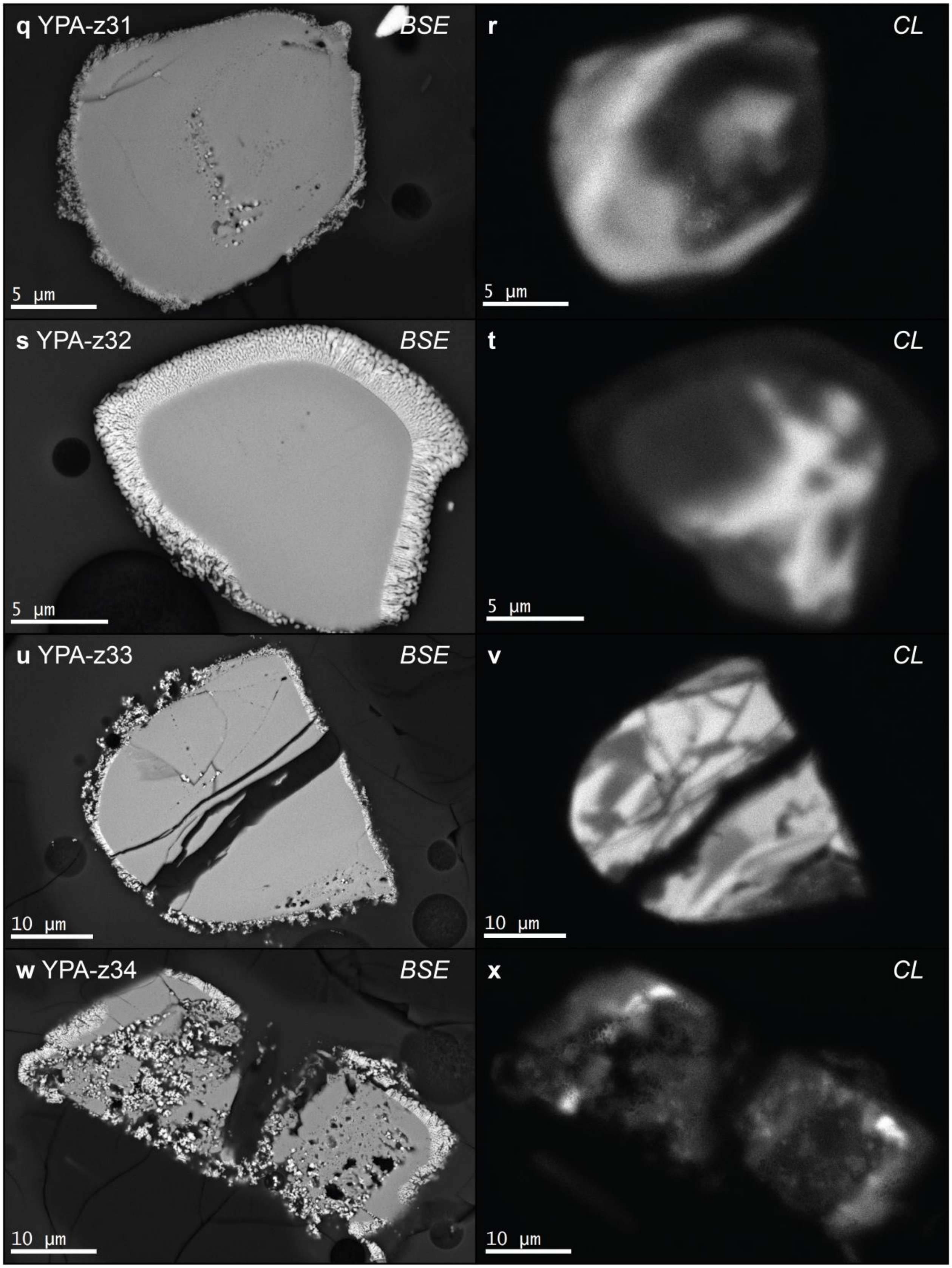

Supplementary Fig. 8 continued.

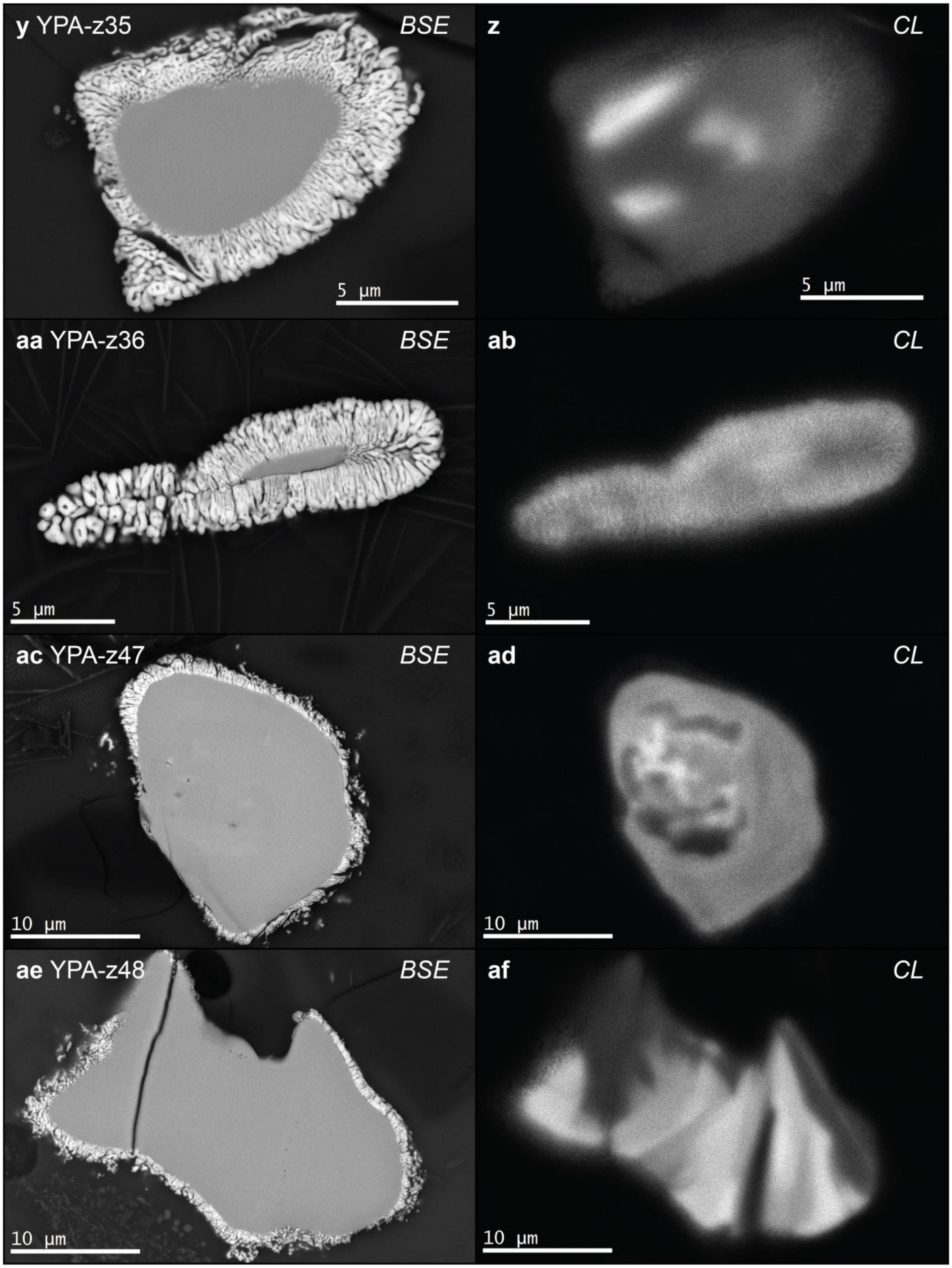

Supplementary Fig. 8 continued.

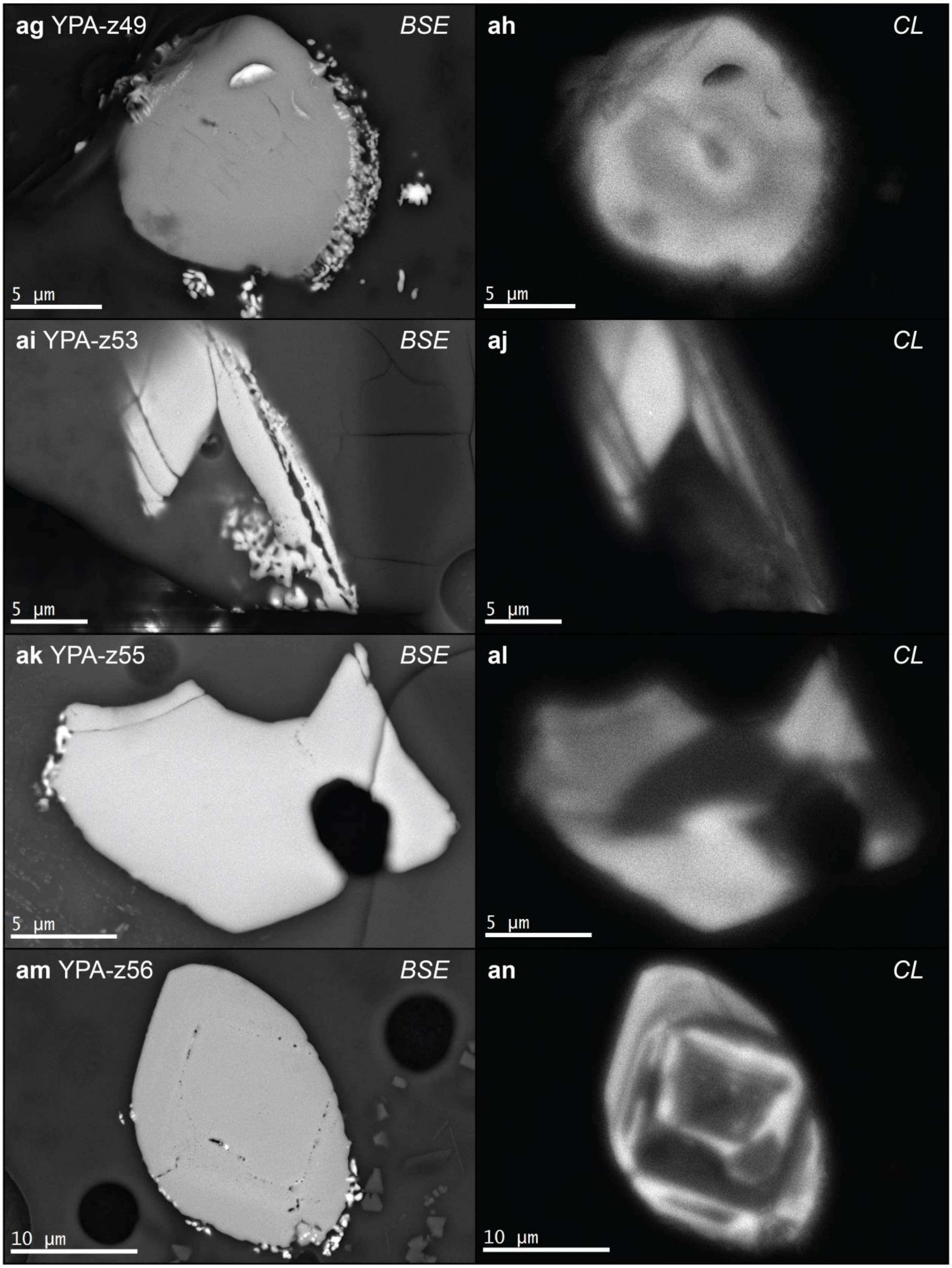

Supplementary Fig. 8 continued.

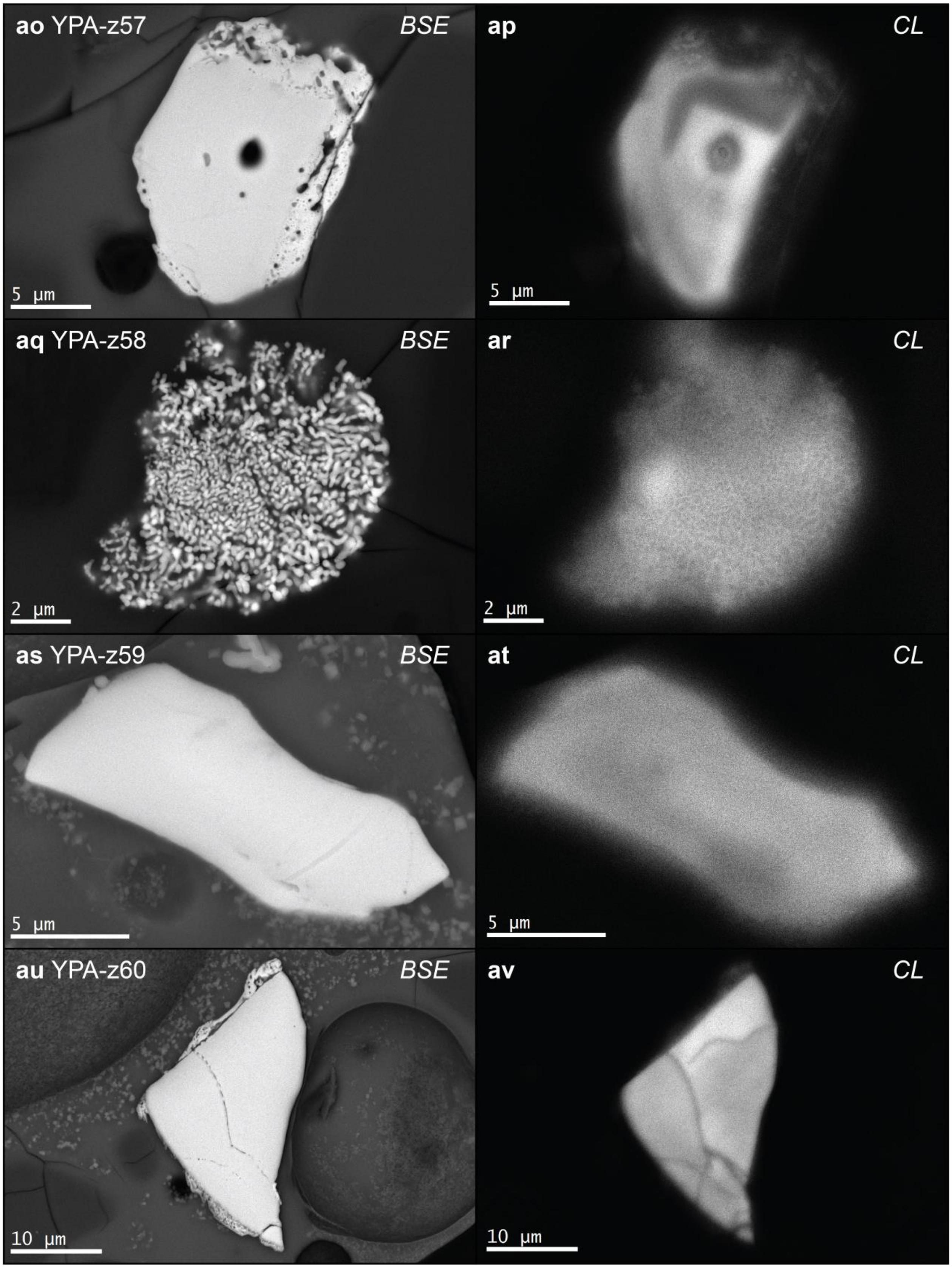

Supplementary Fig. 8 continued.

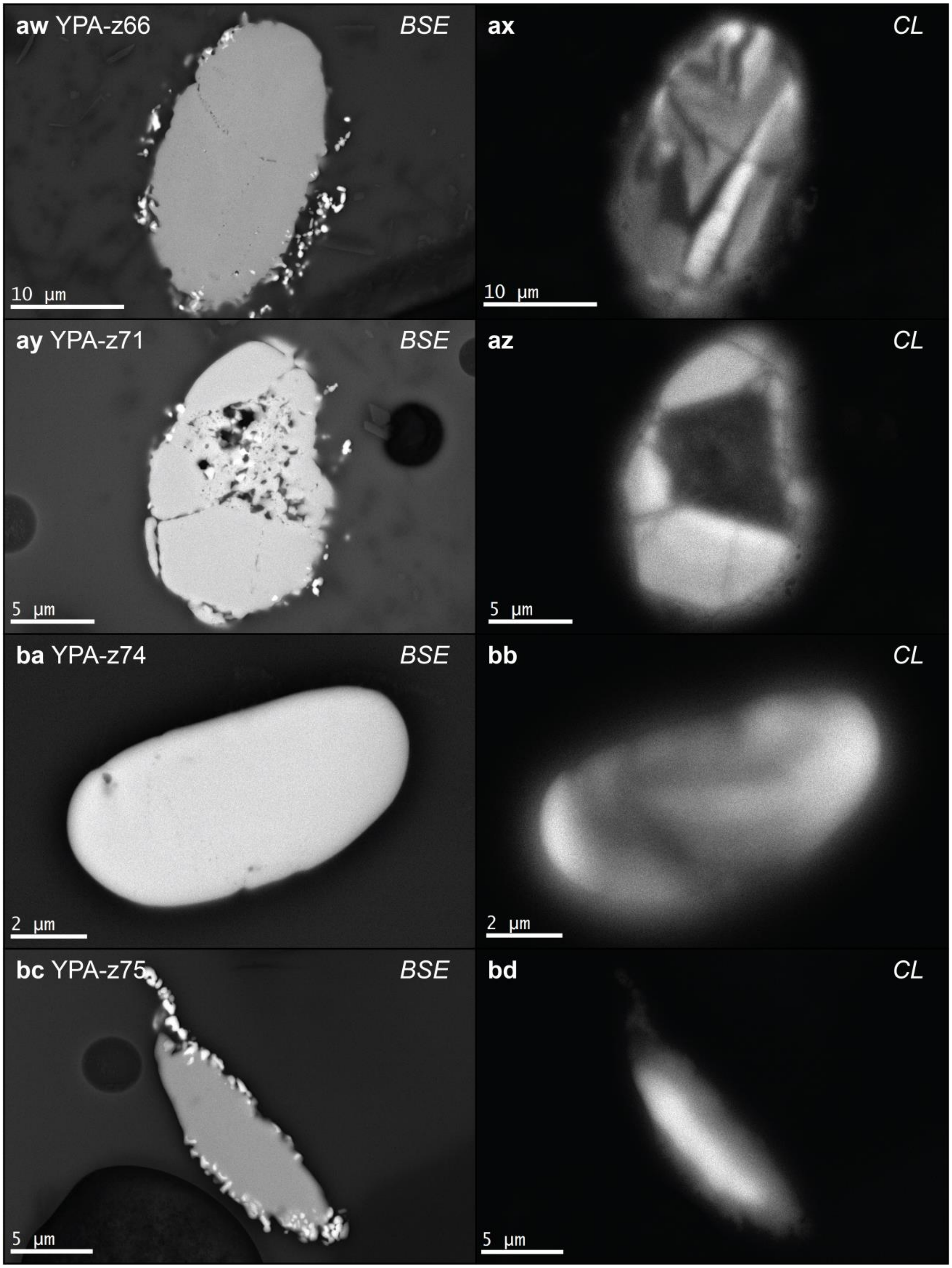

Supplementary Fig. 8 continued.

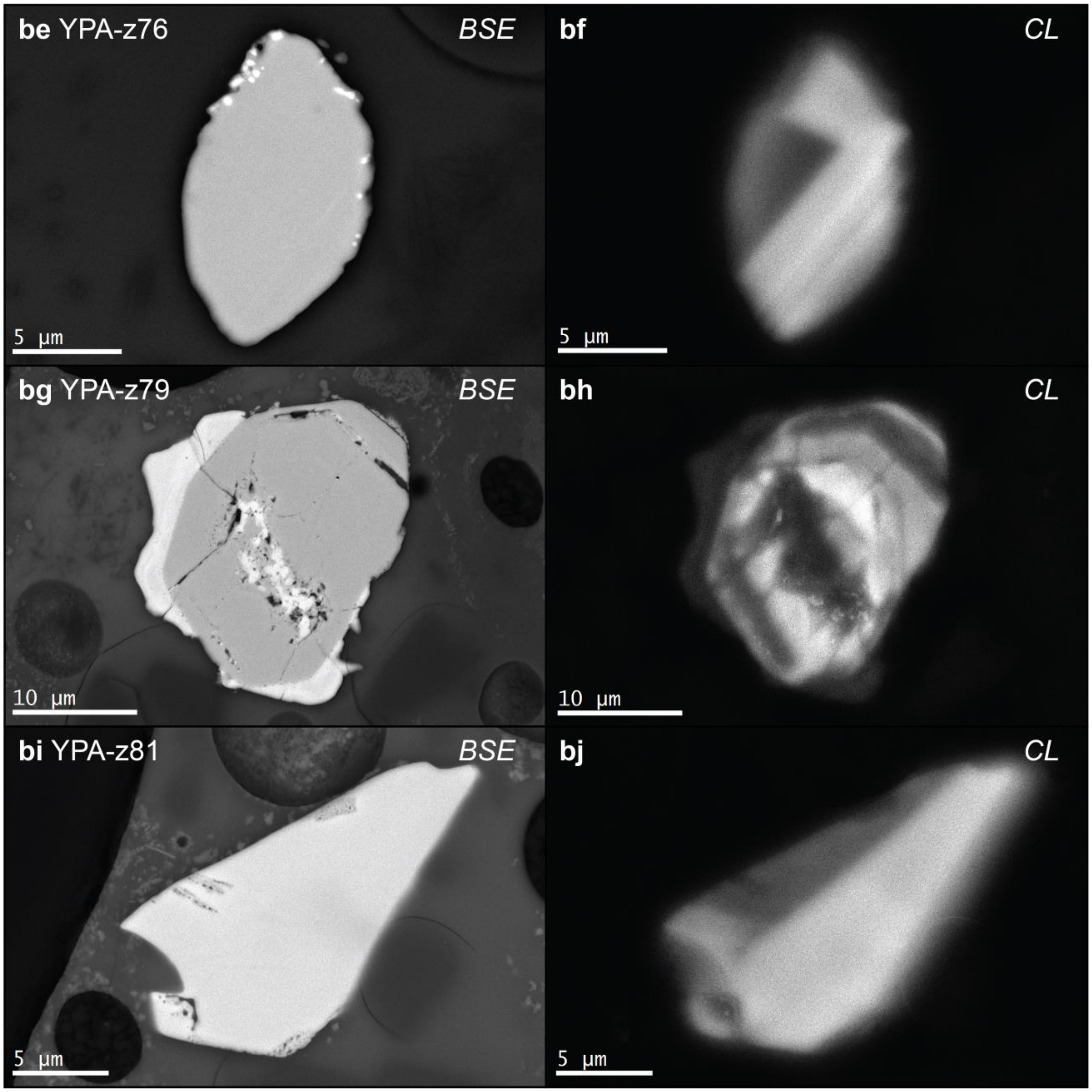

Supplementary Fig. 8 continued.

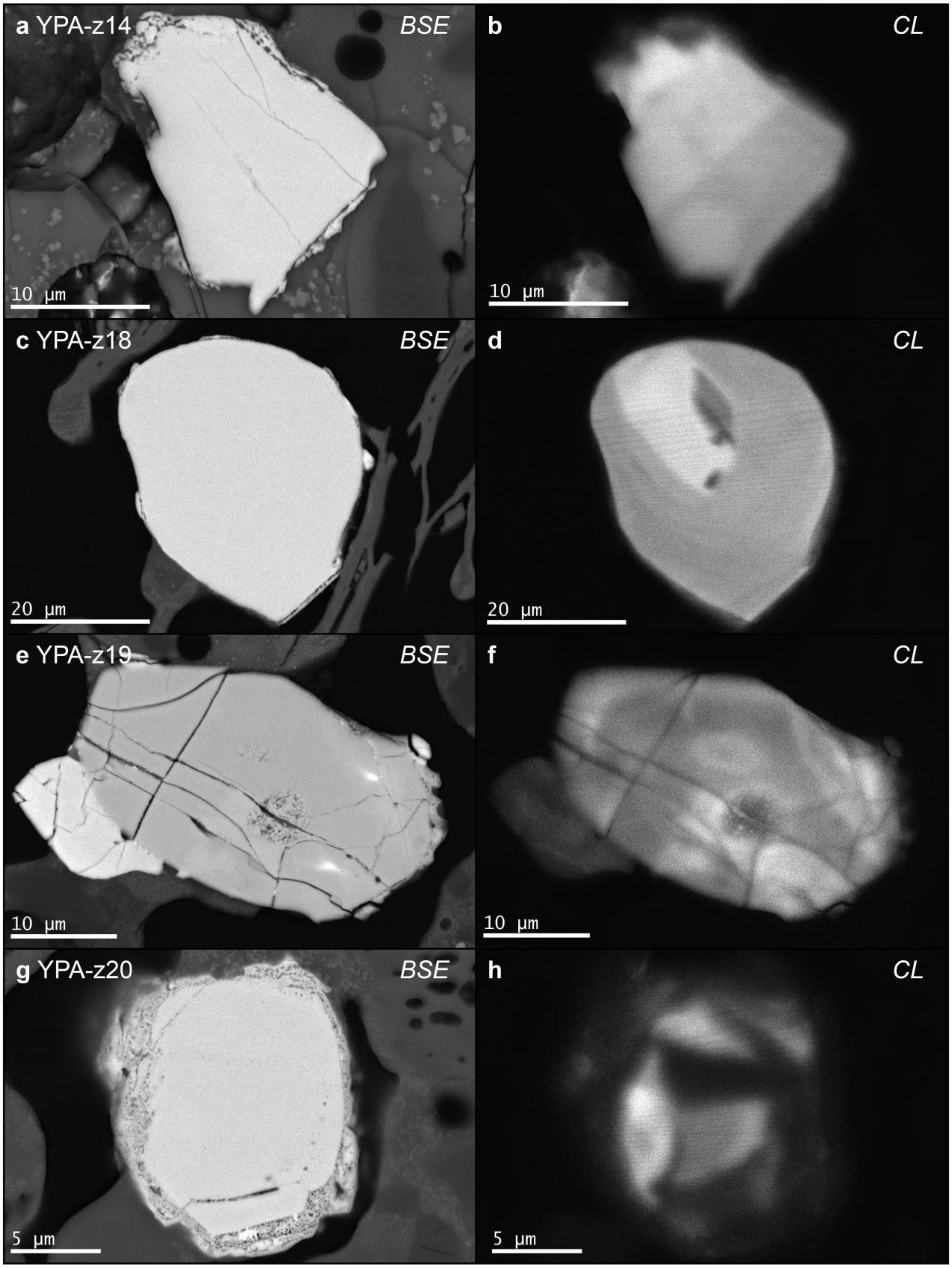

Supplementary Fig. 9. Backscattered electron (BSE), left panel, and cathodoluminescence (CL), right panel, imaging of all zircon grains from domain 3 (fused clasts) of the York County fulgurite.

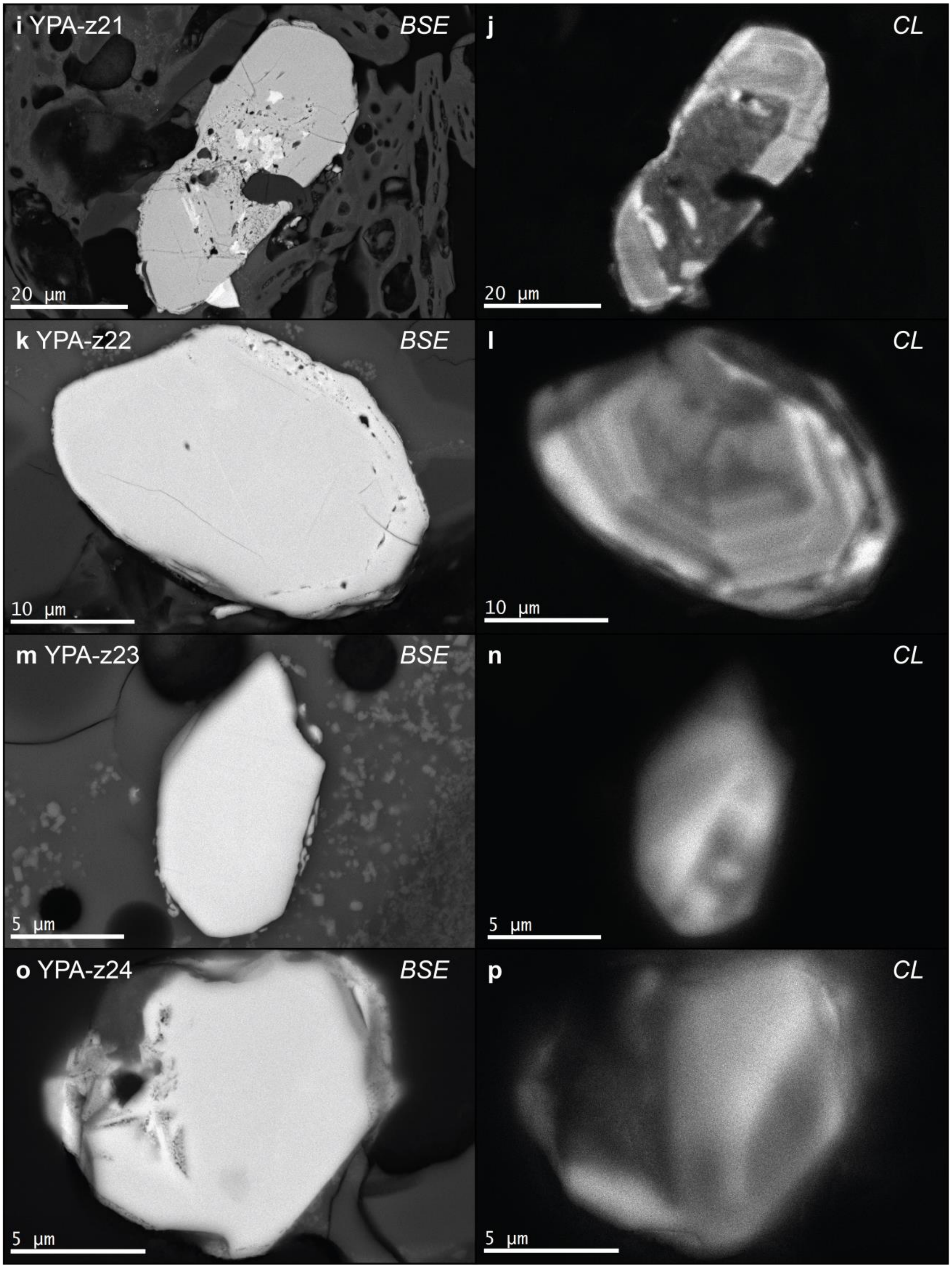

Supplementary Fig. 9 continued.

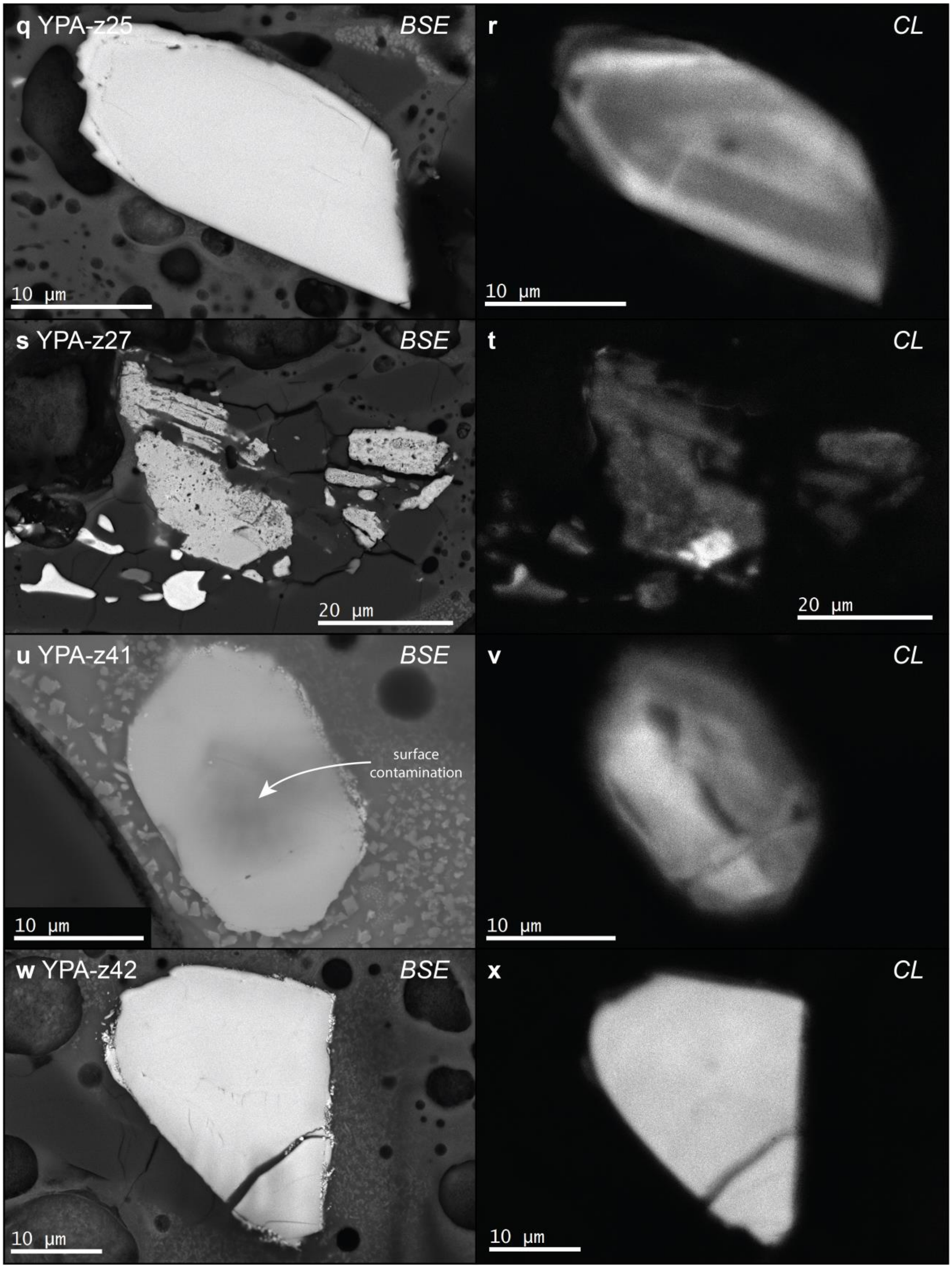

Supplementary Fig. 9 continued.

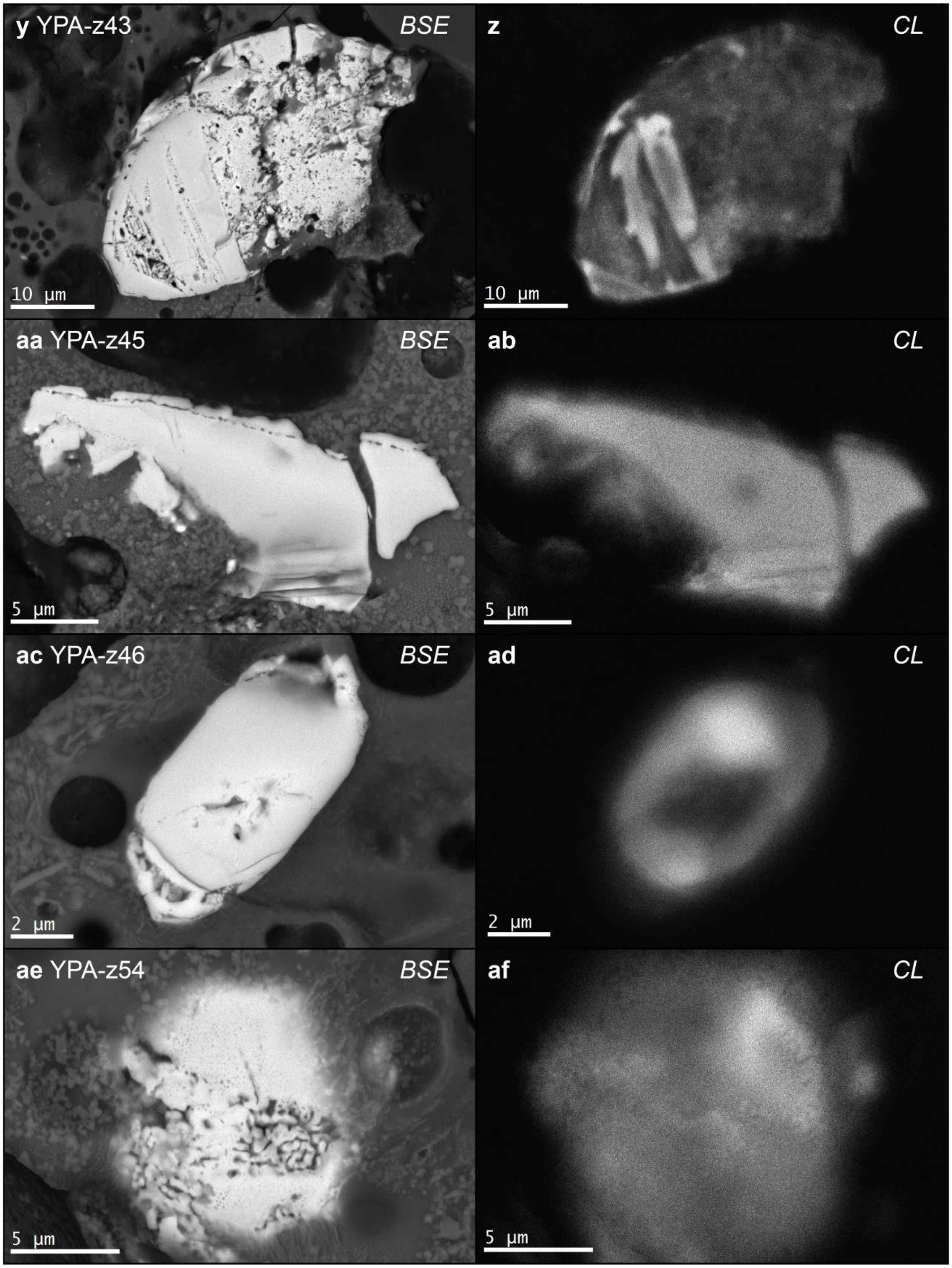

Supplementary Fig. 9 continued.

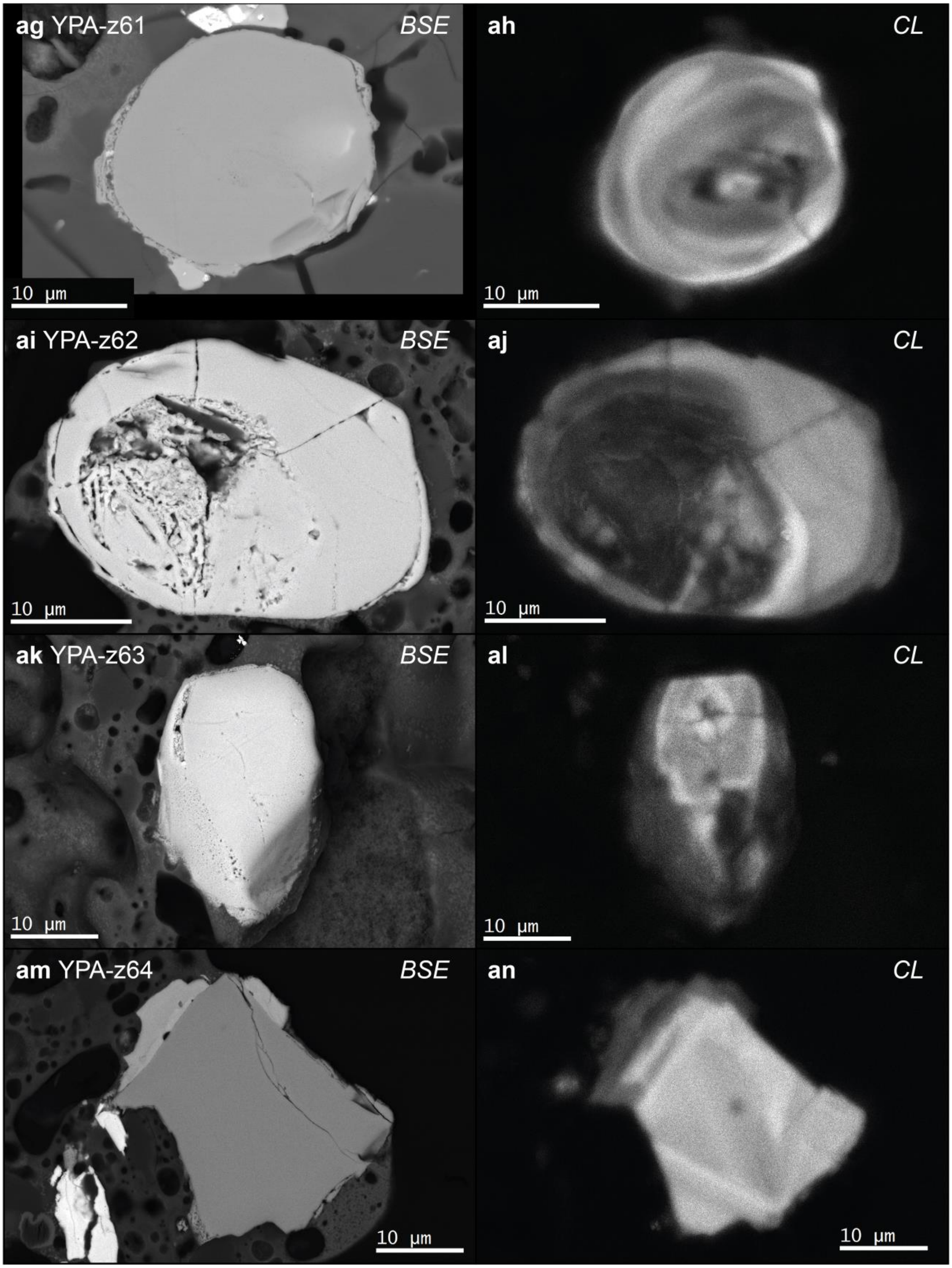

Supplementary Fig. 9 continued.

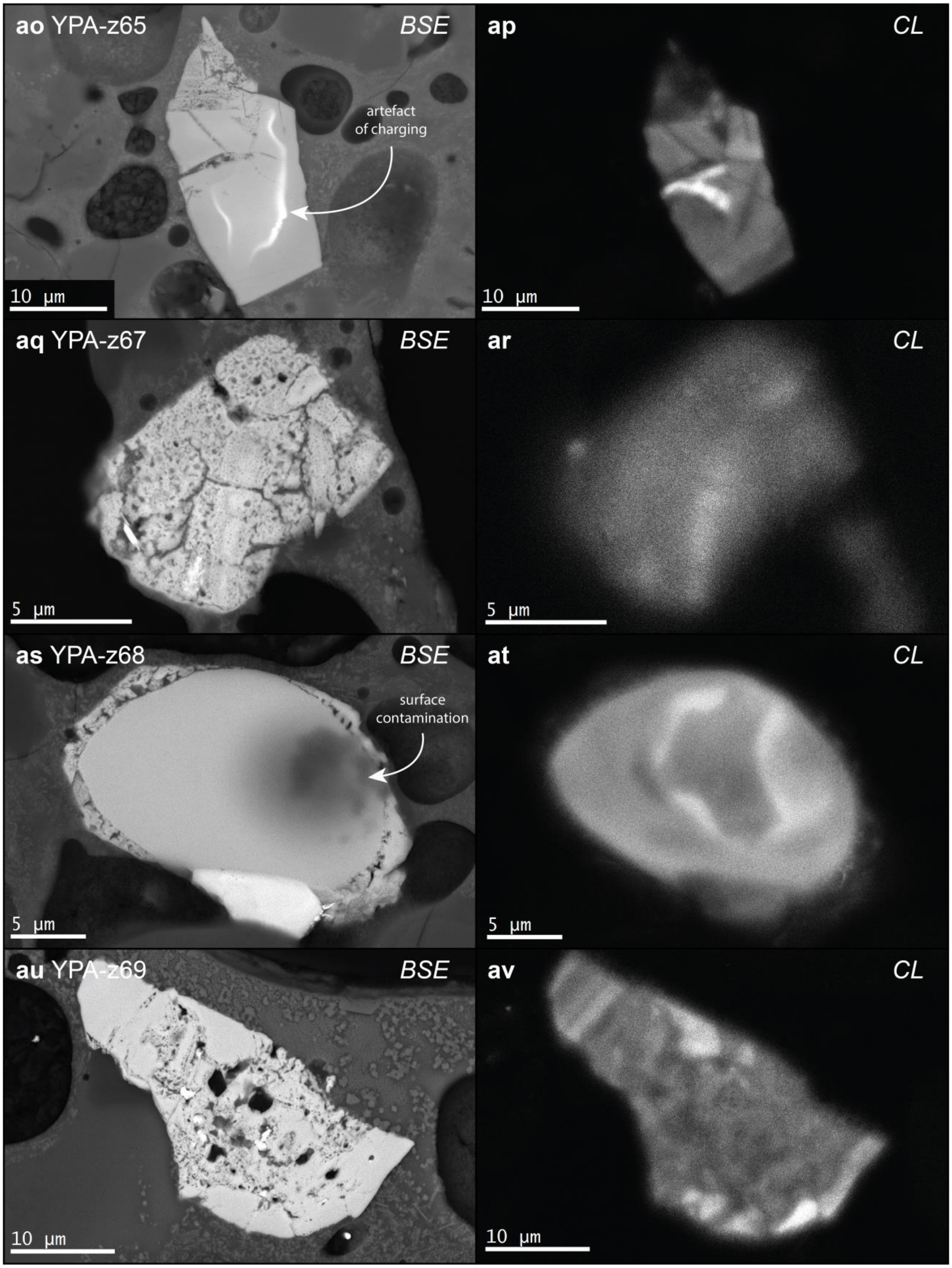

Supplementary Fig. 9 continued.

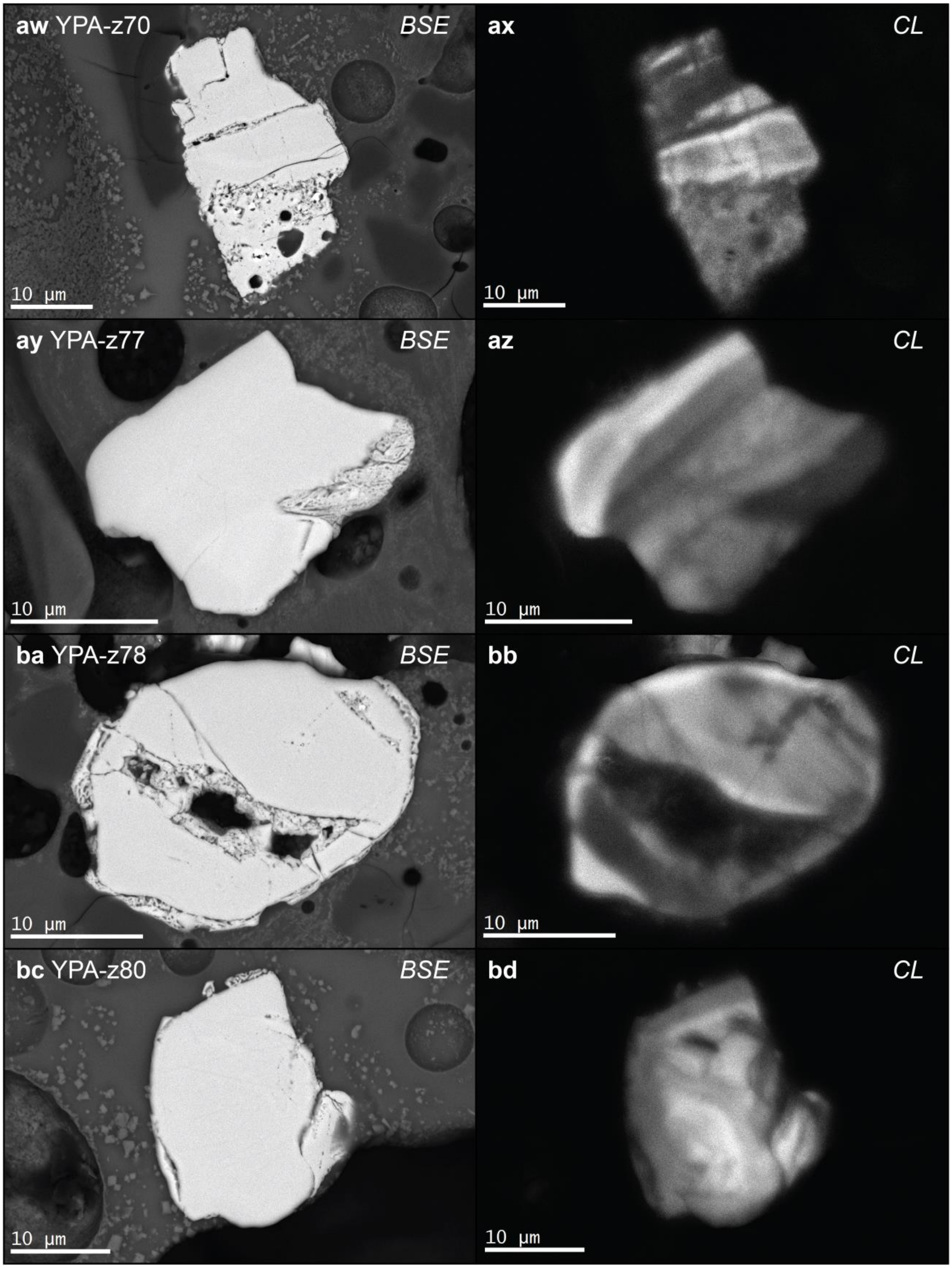

Supplementary Fig. 9 continued.

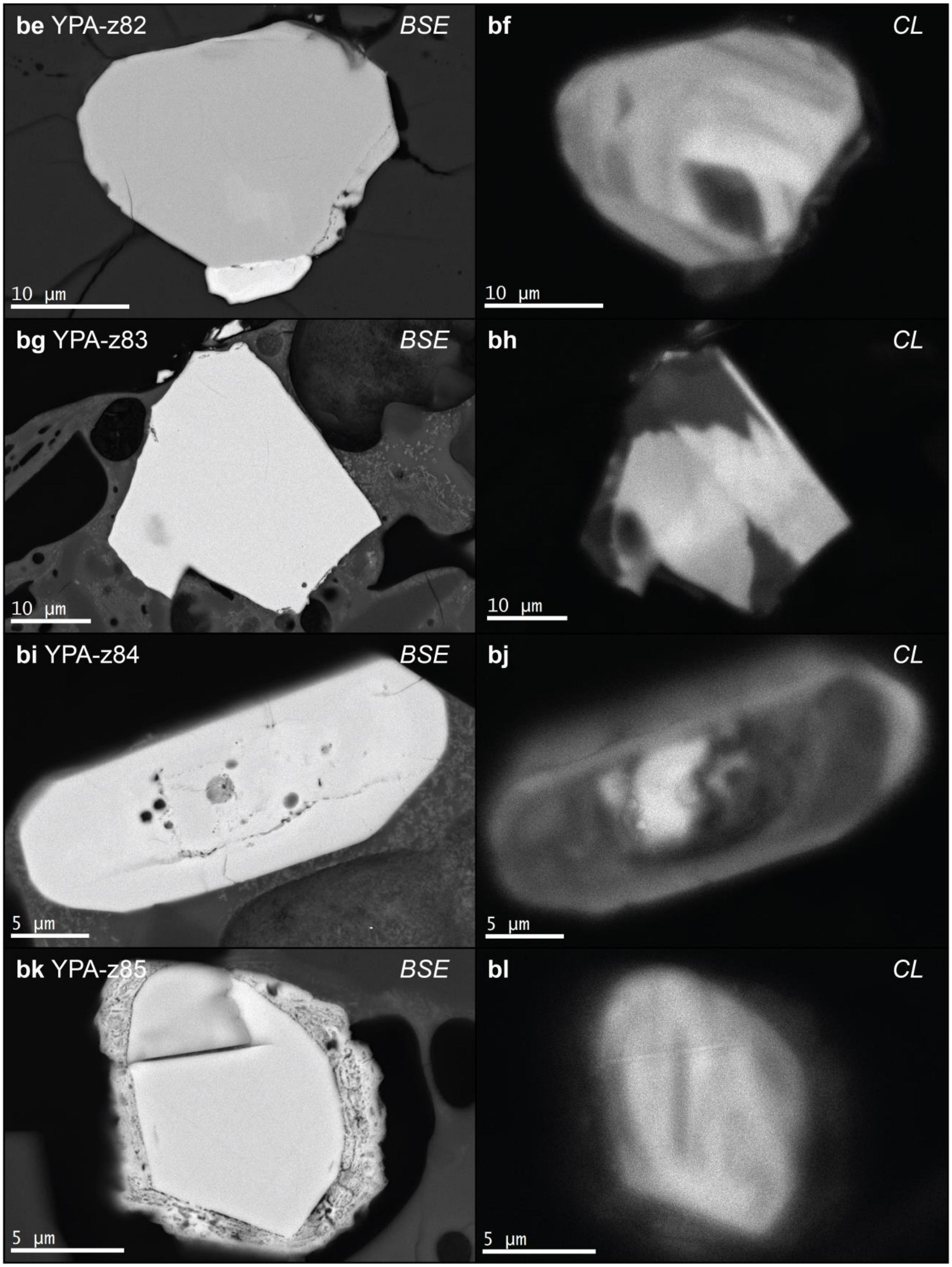

Supplementary Fig. 9 continued.

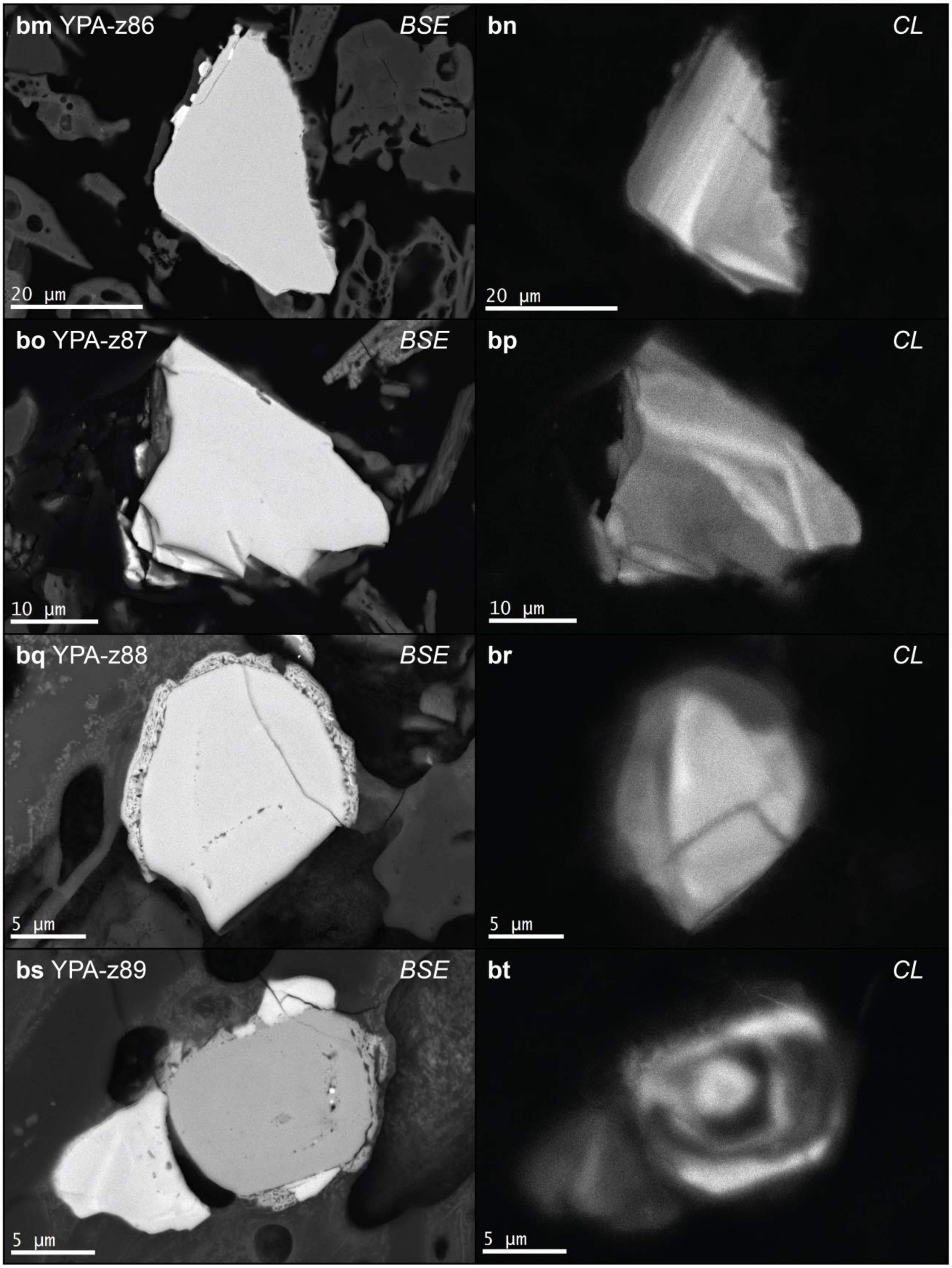

Supplementary Fig. 9 continued.

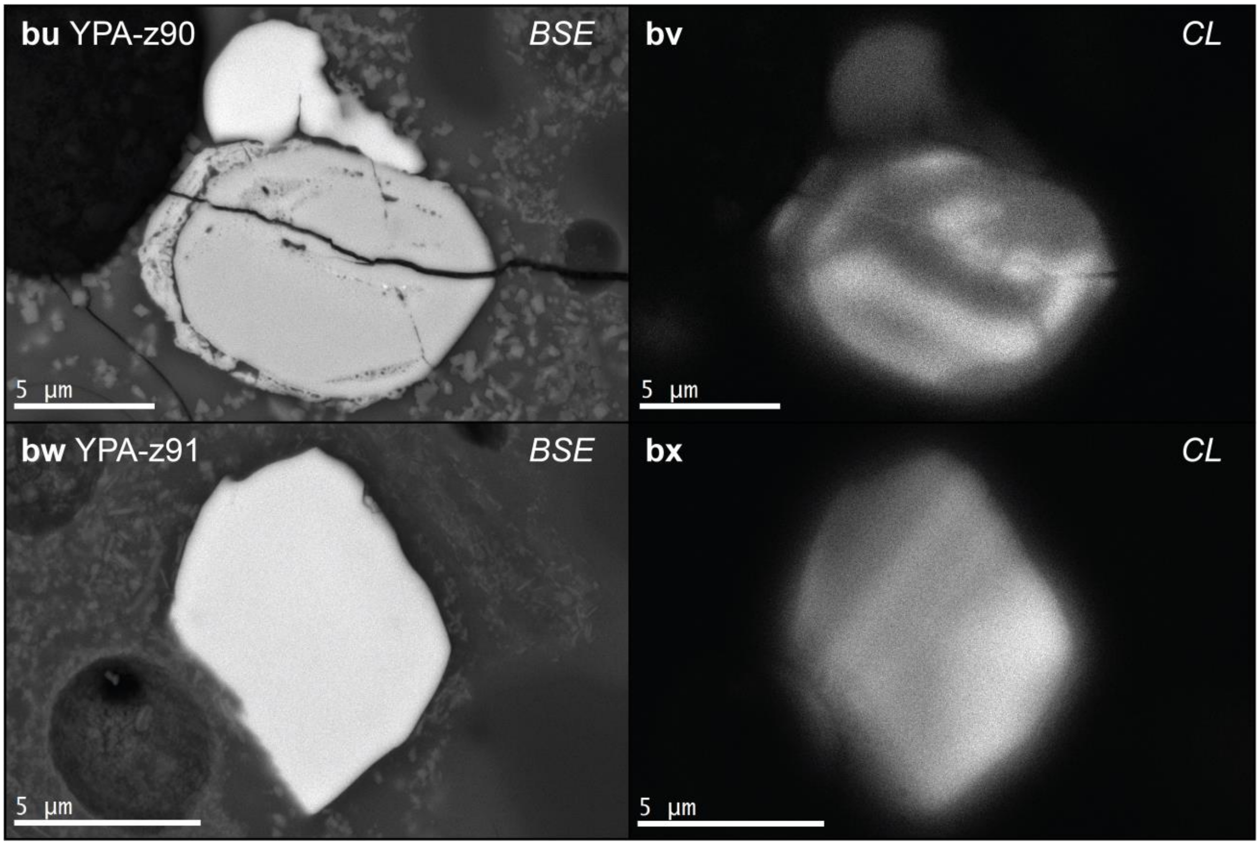

Supplementary Fig. 9 continued.

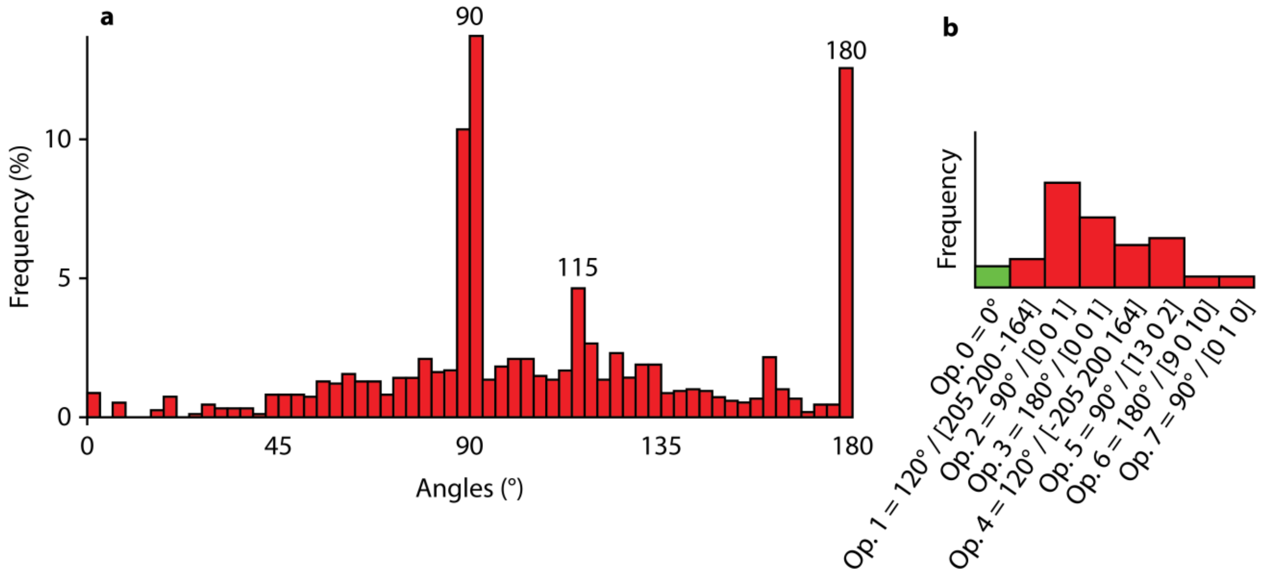

Supplementary Fig. 10. Results from orientation analysis of monoclinic zirconia, baddeleyite, in YPA-z5 (Fig. 5a-d) using the ARPGE software of ref. [1]. **a** Histogram of disorientation angles between 'daughter' grains of monoclinic zirconia. **b** Frequency of disorientation symmetry operators for adjacent 'daughter' grains for operators for the type 2 orientation relationship of ref. [2]. Op. – operator.

References

1. Cayron, C. ARPGE: a computer program to automatically reconstruct the parent grains from electron backscatter diffraction data. *J. Appl. Crystallogr.* **40**, 1183–1188 (2007).
2. Cayron, C., Douillard, T., Sibil, A., Fantozzi, G. & Sao-Jao, S. Reconstruction of the cubic and tetragonal parent grains from electron backscatter diffraction maps of monoclinic zirconia. *J. Am. Ceram. Soc.* **93**, 2541–2544 (2010).
